# Laying Waste to Mercury: Inexpensive Sorbents Made from Sulfur and Recycled Cooking Oils

**DOI:** 10.1002/chem.201702871

**Published:** 2017-08-30

**Authors:** Max J. H. Worthington, Renata L. Kucera, Inês S. Albuquerque, Christopher T. Gibson, Alexander Sibley, Ashley D. Slattery, Jonathan A. Campbell, Salah F. K. Alboaiji, Katherine A. Muller, Jason Young, Nick Adamson, Jason R. Gascooke, Deshetti Jampaiah, Ylias M. Sabri, Suresh K. Bhargava, Samuel J. Ippolito, David A. Lewis, Jamie S. Quinton, Amanda V. Ellis, Alexander Johs, Gonçalo J. L. Bernardes, Justin M. Chalker

**Affiliations:** ^1^ School of Chemical and Physical Sciences Flinders University Bedford Park South Australia Australia; ^2^ Centre for NanoScale Science and Technology Flinders University Bedford Park South Australia Australia; ^3^ Instituto de Medicina Molecular Faculdade de Medicina da Universidade de Lisboa Lisbon Portugal; ^4^ Environmental Sciences Division Oak Ridge National Laboratory Oak Ridge Tennessee USA; ^5^ Flinders Analytical, School of Chemical and Physical Sciences Flinders University Bedford Park South Australia Australia; ^6^ Centre for Advanced Materials & Industrial Chemistry (CAMIC), School of Science RMIT University Melbourne Victoria Australia; ^7^ School of Engineering RMIT University Melbourne Victoria Australia; ^8^ School of Chemical and Biomedical Engineering University of Melbourne Parkville Victoria Australia; ^9^ Department of Chemistry University of Cambridge Cambridge United Kingdom

**Keywords:** inverse vulcanisation, mercury, sulfur, sulfur polymer, waste valorisation

## Abstract

Mercury pollution threatens the environment and human health across the globe. This neurotoxic substance is encountered in artisanal gold mining, coal combustion, oil and gas refining, waste incineration, chloralkali plant operation, metallurgy, and areas of agriculture in which mercury‐rich fungicides are used. Thousands of tonnes of mercury are emitted annually through these activities. With the Minamata Convention on Mercury entering force this year, increasing regulation of mercury pollution is imminent. It is therefore critical to provide inexpensive and scalable mercury sorbents. The research herein addresses this need by introducing low‐cost mercury sorbents made solely from sulfur and unsaturated cooking oils. A porous version of the polymer was prepared by simply synthesising the polymer in the presence of a sodium chloride porogen. The resulting material is a rubber that captures liquid mercury metal, mercury vapour, inorganic mercury bound to organic matter, and highly toxic alkylmercury compounds. Mercury removal from air, water and soil was demonstrated. Because sulfur is a by‐product of petroleum refining and spent cooking oils from the food industry are suitable starting materials, these mercury‐capturing polymers can be synthesised entirely from waste and supplied on multi‐kilogram scales. This study is therefore an advance in waste valorisation and environmental chemistry.

## Introduction

Mercury pollution threatens the health and safety of millions of humans across the globe.^1^ This neurotoxic metal is encountered in many industrial activities including coal combustion, oil and natural gas refining, waste incineration, chloralkali plant operation and waste discharge, and various metallurgic processes.^2^ Mercury is used intentionally in artisanal and small‐scale gold mining (ASGM)1a and in agricultural practices that still rely on fungicides that contain highly toxic alkylmercury derivatives.^3^ ASGM is especially problematic, with widespread and increasing incidence in developing nations due to rising gold prices.^4^ In this practice, liquid mercury is mixed with crushed ore in order to extract gold as an amalgam. The amalgam is then isolated by hand and then heated with a torch to vaporise the mercury and separate it from the gold.^5^ About 12–15 % of the world's gold is generated in this way through the efforts of approximately 15 million miners, many of whom are children.4a It is estimated that, each year, up to 1400 tonnes of mercury are released to land and water due to ASGM alone,4a with devastating effects on the health of miners and children in these communities.^6^ Because mercury pollution from ASGM occurs primarily in low‐income nations, cost‐effective and technologically simple methods for remediation are urgently needed. These crises have been highlighted in news reports in recent years,^7^ and at least one national emergency has been declared in response to mercury pollution due to gold mining.7d

Increasing regulation of mercury emissions is on the horizon, with the Minamata Convention entering full force this year.^8^ In order to comply with these regulations, it is imperative that versatile and inexpensive mercury sorbents be introduced.2a, 9 Additionally, sorbents that can be deployed across large geographic areas are important in remediation efforts associated with practices such as ASGM that may result in the contamination of thousands of acres of land.7d Currently, high performance activated carbons and silver impregnated zeolites are widely used as mercury sorbents in the petroleum and waste sectors.2b While these sorbents are effective in continuous industrial processes, the cost is still too‐often prohibitive in non‐commercial efforts to remediate contaminated ecosystems of large area.^9^, ^10^ Additionally, activated carbon is highly flammable^11^ and often requires an oxidant additive (e.g. immobilised sulfur, bromine, or chlorine) to convert mercury metal to an immobilised mercury(II).^12^ And while the investigation of economical sorbents such as used vehicle tires,^13^ clays,^14^ and various forms of biomass^14^ is encouraging, these materials act primarily as a ligands for Hg^2+^. A general sorbent for mercury must accommodate the many forms commonly encountered in remediation including liquid mercury metal, matrix‐bound mercury metal, mercury vapour, organomercury compounds and inorganic mercury complexed to organic ligands such as humic matter.2a, 9 In an effort to address these problems, we herein introduce sulfur polymers, made through the co‐polymerisation of sulfur and cooking oils (including waste cooking oils), that capture diverse forms of mercury pollution in air, water and soil.

Elemental sulfur is a readily available and inexpensive material produced in excess of 50 million tonnes each year as a by‐product of petroleum refining.^15^ Elemental sulfur can capture and stabilise mercury,^16^ but it suffers from several chemical and physical limitations that make it inconvenient to use directly in remediation. For example, elemental sulfur is flammable with a low ignition temperature (190 °C), it readily sublimes, it is prone to caking and increases hydraulic resistance during filtration, it does not wet and mix well in batch processing of waste fluids, and it is difficult to prepare as durable particles of a desired size.15a, 17 Furthermore, sulfur may decompose in the environment to sulfate, which can increase the abundance of sulfate‐reducing bacteria that are the primary producers of the highly toxic methylmercury in soils and sediments.^18^ There is therefore an interest to discover new forms of sulfur that benefit from the high affinity of this chalcogen for mercury, but do not suffer from the limitations of elemental sulfur noted here.

Recently, the synthesis of polysulfides by inverse vulcanisation^19^ has ushered in a new class of materials with high sulfur content. Pioneered by Pyun, Char, and co‐workers,^19^, ^20^ this process involves melting elemental sulfur and then heating it above its floor temperature of 159 °C. Thermal homolysis of S−S bonds in S_8_ leads to radical ring‐opening polymerisation.^17^, ^19^ Subsequent trapping of the thiyl radical end groups of the sulfur polymers with a polyene provides a cross‐linked polysulfide.^19^ The polymers formed by inverse vulcanisation have been explored in a variety of contexts due to their interesting optical, electrochemical and self‐healing properties.^20^, ^21^ Our laboratory recently introduced a polysulfide prepared by the inverse vulcanisation of the renewable plant oil limonene, and explored its use in the remediation and sensing of Hg^2+^ in water.^22^ Further studies lead by Hasell^23^ and Theato^24^ revealed effective ways to increase the surface area of polymers prepared by inverse vulcanisation (by foaming or electrospinning, respectively) in order to increase performance in Hg^2+^ capture. While these studies motivate deployment of polysulfides for mercury remediation, the cost, scalability, and ease of use are issues that must be addressed before uptake is feasible.4b Additionally, these preliminary reports^22^, ^23^, ^24^ only studied the purification of water containing inorganic HgCl_2_, so it is not yet established whether these sulfur polymers are effective in capturing mercury metal, inorganic mercury bound to natural organic matter (Hg‐NOM),^25^ or organomercury compounds—forms of mercury pollution commonly encountered in the field. We therefore set out to identify polysulfides made from feedstocks that are highly abundant, very inexpensive and easy to handle, and then tested them on diverse forms of mercury pollution in air, water and soil.

**Figure 1:**
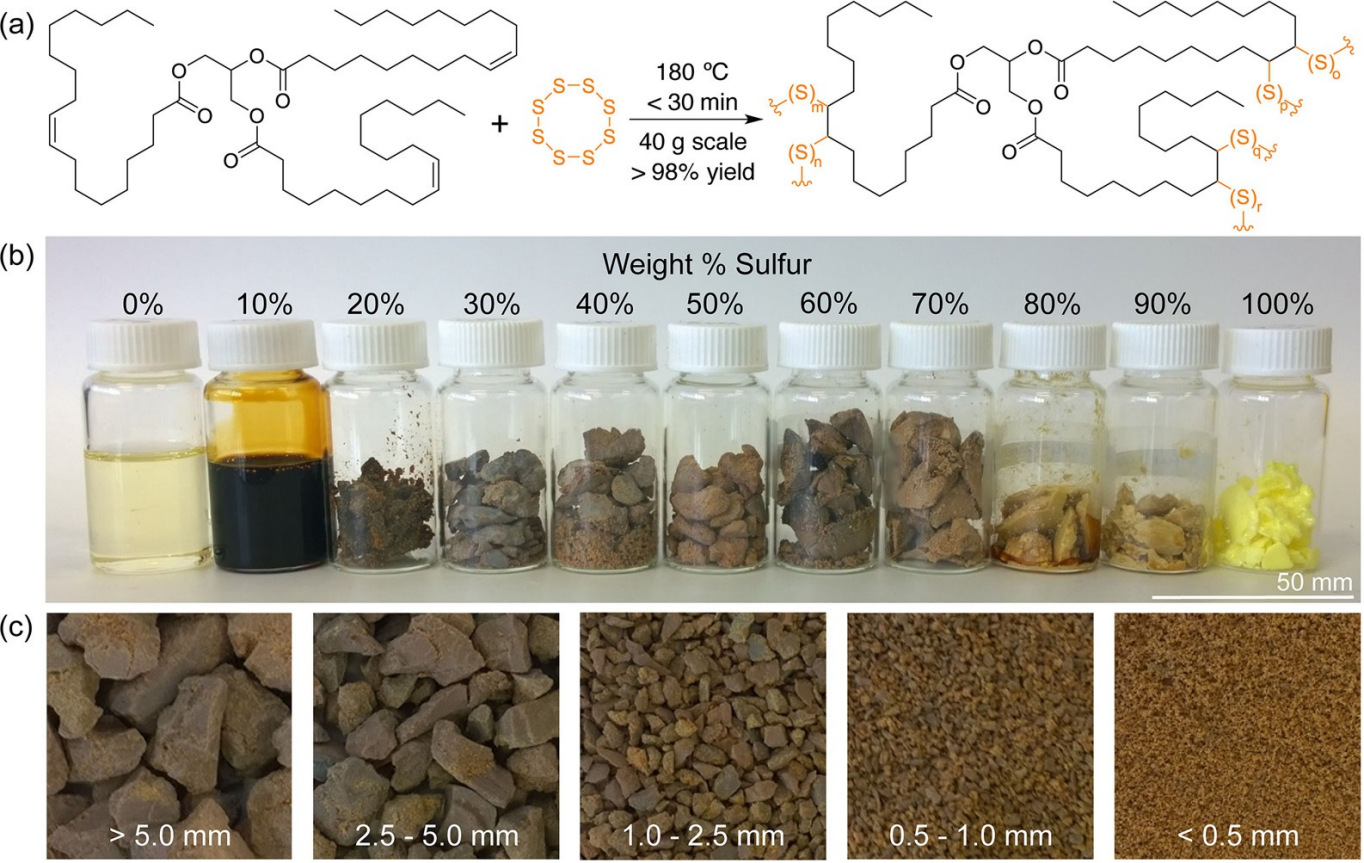
A polysulfide rubber with high sulfur content was formed by the reaction of elemental sulfur with canola oil, sunflower oil, or olive oil. (a) General structure of a plant oil triglyceride (oleic acid is shown here as the major fatty acid component) and the product formed by co‐polymerisation with sulfur. (b) Photograph of the product formed by the reaction of canola oil and sulfur, with varying weight percentages of sulfur. (c) Photographs of the canola oil polysulfide (50 % sulfur) after passing through sieves.

Unsaturated oils from rapeseed, sunflower, and olive plants are attractive as chemical building blocks because they are renewable and can be produced on all inhabited continents.^26^ The alkene functional groups in these triglycerides also provide the requisite points for cross‐linking during inverse vulcanisation. It was anticipated that the *Z* stereochemistry of these alkenes, imparting strain to the olefin, would facilitate rapid reaction with sulfur radicals produced in inverse vulcanisation (Figure 1 a). Historically, the reaction of sulfur and unsaturated plant oils has been used to make factice and ebonite. Factice is a gel‐like modifier used in the manufacture of various rubbers and pencil erasers, typically prepared with up to 25 % sulfur by weight.^27^ Ebonite is a hard and durable building material formed by the prolonged heating of sulfur (≈30–50 wt %) with natural rubber, often in the presence of unsaturated additives such as linseed oil.^28^ We reasoned that inverse vulcanisation of unsaturated plant oils would provide a variant of these materials with very high sulfur content (50 % or more sulfur by mass). Following similar logic, Theato and co‐workers also explored the inverse vulcanisation of linseed, sunflower, and olive oils, and used these polymers as cathode materials.^29^ Here we considered that *used* cooking oils (often comprised of canola and sunflower oils) could be recycled and employed as a starting material. Both sulfur and cooking oils are produced in multi‐million tonnes each year, so the large‐scale supply of raw materials would be addressed at the outset.^26^, ^30^ Additionally, the high levels of sulfur in the proposed co‐polymer were anticipated to impart high affinity for various forms of mercury. Finally, because sulfur is a by‐product of petroleum refining15b and used cooking oils are a by‐product of the food industry,^31^ there is the intriguing prospect of making a mercury‐binding polymer, in a single, solvent‐free step, in which every atom in the product is derived from industrial waste.21d

## Results and Discussion

### Polymer synthesis

As a starting point, the reaction between sulfur and food grade canola oil was investigated. In the event, sulfur was first melted and then heated further to 180 °C to initiate ring‐opening polymerisation. An equal mass of canola oil was then added slowly to maintain an internal temperature of approximately 180 °C. The reaction was initially two phases, so rapid stirring was used to ensure efficient mixing (Figure S1). After 10 minutes the mixture appeared to form one phase and within 20 minutes of total reaction time, a solid brown rubber formed (Figure 1). Essentially quantitative yields were obtained and no solvents or exogenous reagents were required in the synthesis. A similar material was produced using both sunflower and olive oil (Figure S2), though sunflower oil typically reached its gel point within 10 minutes of total reaction time at 180 °C. We attributed this difference in time required to reach the gel point to the variation in unsaturation between the plant oils. These differences were determined by conversion of the vegetable oils to their fatty acid methyl esters by treatment with sodium methoxide in methanol (Figure S3). Analysis of these esters by GC‐MS revealed a far higher percentage of polyunsaturated linoleic acid in sunflower oil (50 %) compared to canola oil (14 %) and olive oil (9 %). Oleic acid was the major fatty acid component in the canola oil and olive oil triglyceride, making up about 78 % of the fatty acids in both oils (Figure S4–S5).

Subsequent experiments focused on canola oil because of its widespread use in the food industry.26b, 31 The amount of sulfur that could be incorporated into the polymer was therefore investigated (Figure 1 b). At 10 % sulfur by weight, a viscous oil was obtained. From 20 % to 70 % sulfur by weight, a rubber was obtained. With increasing sulfur content, the product became more brittle (Figure S6). The polymer prepared at 50 % sulfur by weight and 50 % canola by weight was selected for subsequent experiments in mercury binding. At this composition, substantial sulfur would be available to capture mercury, and the particles would not be too brittle for use in applications that require filtration or sieving. This composition also ensured that a substantial amount of both sulfur and cooking oil were used to synthesise the polymer—an important consideration in waste valorisation.

The inverse vulcanisation reaction using canola oil was easily scaled to 40 g total polymer without incident. Larger batches are likely possible, but this scale allowed for relatively uniform mixing and temperature control. Running these reactions in parallel batch reactors allowed us to make more than 10 kg of this polymer to date. To prepare the polymer as particles, the rubber was milled in a blender to give particles less than 12 mm in diameter. These particles could be further partitioned according to size by passing through sieves (Figure 1 c). Finally, when waste cooking oil obtained from a local café was used in the synthesis, there was no substantial difference in the polymerisation when compared to pure canola oil purchased from a supermarket (Figure S7). In this way, the polysulfide polymer was derived entirely from industrial waste.

### Polymer characterisation

Reaction of sulfur at the alkenes in the canola oil was consistent with the disappearance of the C=C stretch at 1613 cm^−1^ and the alkene C−H stretch at 3035 cm^−1^ in the IR spectrum of the polymer (Figure S8). While the product had limited solubility in CDCl_3_, ^1^H NMR of the soluble fraction indicated that alkenes were consumed in the reaction, though the gel point was reached before all alkenes were consumed (Figure S9). The ability of sulfur to react efficiently at the alkene of the fatty acid esters was also inferred by ^1^H NMR spectroscopic analysis of the product formed when the methyl ester derived from each of the plant oils was treated with sulfur under the polymerisation conditions (Figure S10). Notably, the products obtained from the inverse vulcanisation of the fatty acid methyl esters were viscous oils rather than solid polymers, indicating the key structural role the triglycerides play in cross‐linking.

**Figure 2:**
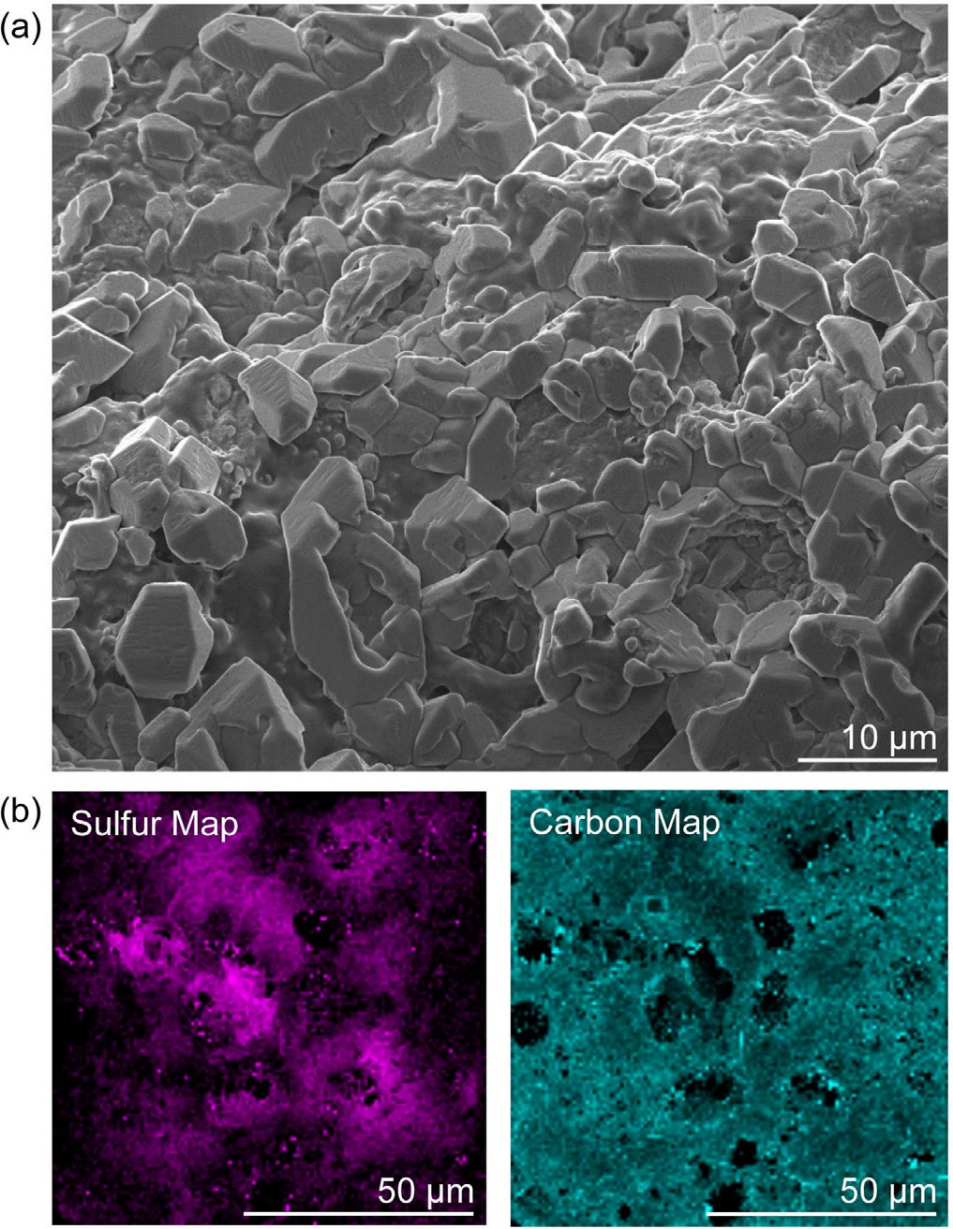
Surface analysis of the canola oil polysulfide. (a) Scanning electron microscopy revealed a locally smooth surface and microscale features. (b) Auger spectroscopic imaging revealed high carbon and sulfur content on the polymer surface, consistent with the canola oil and sulfur monomers used in the synthesis. Representative images are shown.

Analysis of the milled polymer by SEM revealed a locally smooth surface yet a high level of microscale features that imparted high surface area (Figure 2 a and Figure S11). The surface was rich in sulfur and carbon, as indicated by elemental mapping via EDS (Figure S12) and Auger spectroscopy (Figure 2 b and Figures S13–14) and fully consistent with the sulfur and canola oil building blocks. The presence of polysulfides was inferred by confocal Raman microscopy with S−S stretching detected at 432 and 470 cm^−1^ (Figure S15).^22^, ^32^ Interestingly, confocal Raman microscopy also revealed domains of very high sulfur, some of which appeared as sulfur particles embedded in the polymer and on the surface of the polymer (Figure S16). EDS of these domains also indicated very high levels of sulfur (Figure S12). No thiols were detected on the surface, as inferred by the lack of reactivity with thiol‐specific Ellman's reagent (Figure S17).

**Figure 3:**
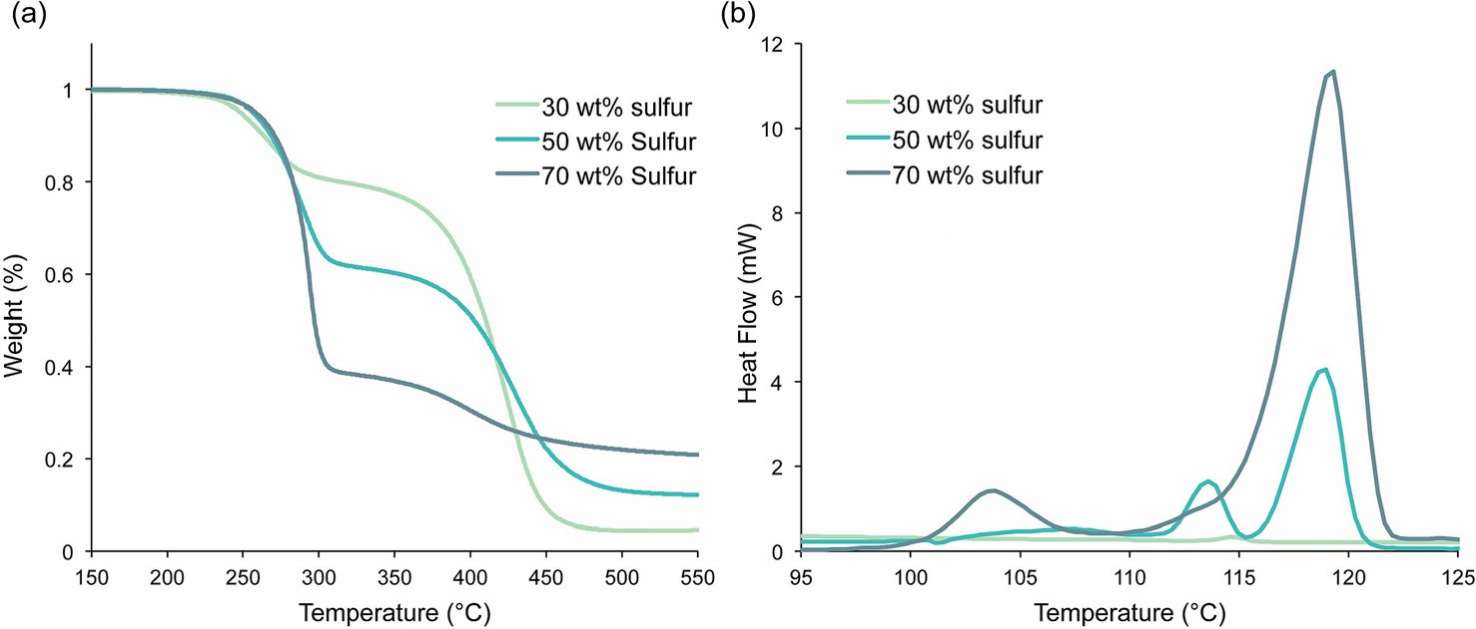
Thermal analysis of the canola oil polysulfide. (a) Thermogravimetric analysis (TGA) of the canola oil polysulfide prepared by inverse vulcanisation at 30, 50, and 70 % sulfur by mass. (b) Differential scanning calorimetry (DSC) of the canola oil polysulfide between 100 and 125 °C revealed that when more than 30 % sulfur was used in the synthesis, free sulfur was detected. For full thermal analysis of the polymers, including comparison to unreacted vegetable oils and elemental sulfur, see pages S24–S31.

Thermal analysis (TGA and DSC) of the canola oil polysulfide revealed several important properties of the polymer. First, thermal degradation featured two major mass losses, with the first onset at 230 °C and the second at 340 °C (Figure 3 a and Figure S18). The first mass loss was due to decomposition of polysulfide domains, as increasing sulfur content was correlated with greater mass loss in the first decomposition at 230 °C (Figure 3 a). The second mass loss was therefore the thermal decomposition of the canola oil domain of the polymer. (Thermal analyses of the unmodified cooking oils and elemental sulfur were also carried out for comparison, Figure S19–S20). DSC revealed that above 30 % sulfur by mass, there was an endotherm between 100 and 150 °C (Figure 3 b). This transition was attributed to the melting range of free sulfur. By integrating each area of these endotherms, an estimate of free sulfur was made (Figure S20–S23). The polysulfide made from 50 % canola oil and 50 % sulfur, for instance, was estimated to contain about 9 % free sulfur by mass. The polysulfides made from 60 and 70 % sulfur, in comparison, were estimated to contain 23 % and 38 % free sulfur, respectively. Considered with the SEM, EDS and Raman data, these results suggested that sulfur reacted with canola oil up to a composition of 30 % sulfur by mass. Above this level, the excess sulfur is trapped in the polymer matrix as microparticles. Similar thermal analyses were observed for polysulfides prepared from sunflower oil, olive oil and used cooking oil (Figures S24–S26). The interpretation of these results was consistent with the characterisation of related polymer composites formed from vegetable oils and sulfur, as reported by Theato and co‐workers.^29^

It was noteworthy that while the IR and Raman spectra of the canola oil polysulfide and commercial factice were similar (Figure S27–S28), the TGA profiles were slightly different. For instance, commercial factice with the highest percentage of sulfur (25 %) had a higher onset of degradation of the sulfur domain (280 °C) compared to the polysulfide prepared by inverse vulcanisation (230 °C) (Figure S29–S30). We therefore wondered if there was a difference in the material formed by inverse vulcanisation (where canola oil is added to a sulfur pre‐polymer at 180 °C) and classic vulcanisation (where sulfur is added portionwise to canola oil at 180 °C—a method of factice production). Executing both protocols with equal masses of canola oil and sulfur on a 40 g reaction scale provided essentially the same rubber material, as indicated by physical appearance, TGA and DSC (Figure S31). Only a very minor difference in endotherm of free sulfur was observed (Figure S32). Therefore, the order of addition of the sulfur and canola oil did not appear to make a major difference in the product obtained on this time scale and temperature. We suspect that the reaction mixture equilibrated to a similar composition of sulfur and polysulfide polymers in both reactions before reaching the gel point. With that said, there may be subtle differences in the products of inverse and classic vulcanisation (such as the number and length of sulfur chains), that are not revealed by the TGA and DSC experiments.

Dynamic mechanical analysis (DMA, Figure S33) was carried out at variable temperature to estimate the glass transition temperature (*T_g_*) of the canola oil polysulfide. To accomplish this, the polymer was synthesised as previously described, except a beaker was used as the reaction vessel. After the synthesis, the rubber was carefully cut into a bar (1.4 cm×0.8 cm×0.2 cm) suitable for DMA. Subsequent DMA analysis revealed the peak of the tangent delta (*T_t_*), an estimate of the *T*_g_, at −9 °C. Independently, a *T*_g_, of −12.2 °C was inferred by DSC (Figure S34).

### Mercury capture from water

**Figure 4:**
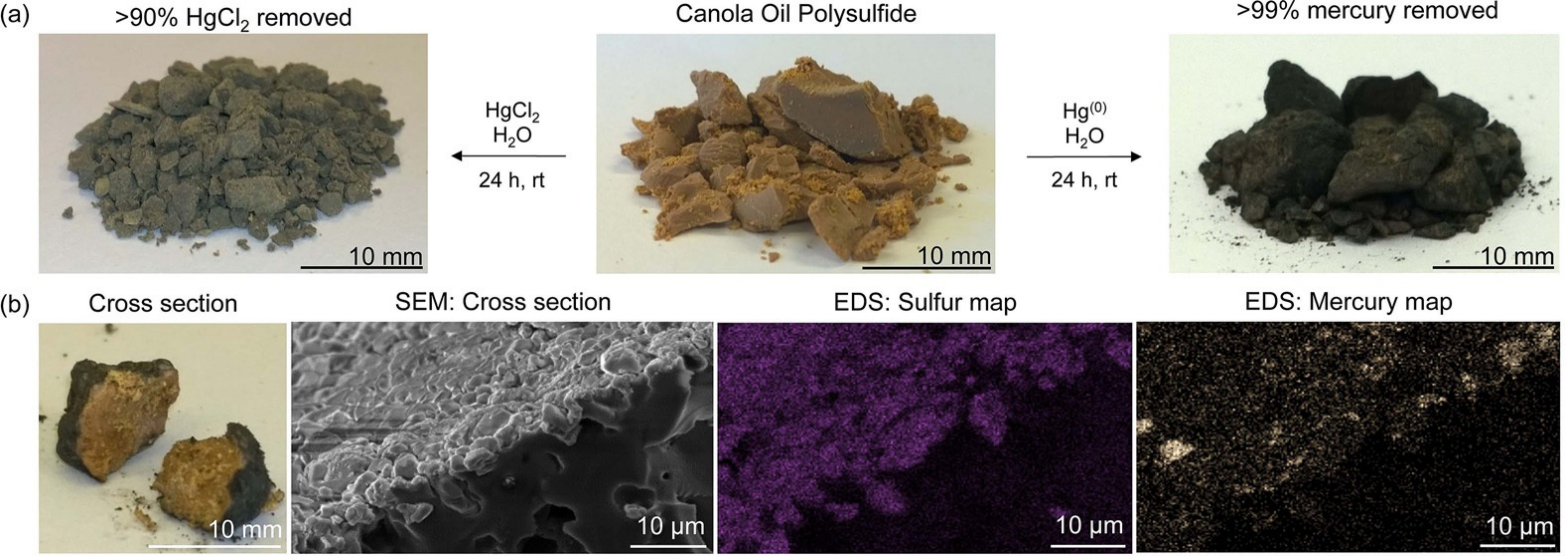
Mercury capture from water. (a) The canola oil polysulfide was effective in capturing both Hg^2+^ and Hg^0^ from water. The polymer changes colour to grey when it binds to Hg^2+^ and to black when it reacts with liquid Hg^0^. (b) EDS analysis confirmed mercury was bound to the surface of the polymer.

Because the polysulfide surfaces were rich in sulfur, affinity for mercury was anticipated. Indeed inorganic polysulfides have been explored to some extent for mercury capture in water, though these materials have limited shelf‐life and need to be prepared as needed.^33^ Before the canola oil polysulfide was tested, the polymer was briefly washed with aqueous NaOH (0.1 m) to ensure no small molecule thiols such as trace H_2_S were present that might confound the mercury binding experiments. This control measure was taken in light of a report by Char, Pyun and co‐workers that H_2_S may be produced during some inverse vulcanisation reactions.^34^ After washing further with water and drying in air, the polymer was then tested for mercury binding. In an initial test, 2.0 g of the canola oil polysulfide (50 % sulfur by weight) was simply incubated, without stirring, in a 5.0 mL aqueous solution of HgCl_2_ (3.5 ppm in Hg^2+^). After 24 hours, the polymer was removed by filtration and the concentration of mercury in the water was quantified by ICP‐MS. Typically 90 % of the soluble mercury was captured after this single treatment, with the treated water containing 0.35±0.1 ppm Hg^2+^ (the average of triplicate experiments). At higher concentrations of HgCl_2_, the polymer performed similarly, with a single treatment of 8.0 g of the polysulfide removing 91 % of Hg^2+^ from a 5.0 mL sample of 74 mm HgCl_2_ after 24 hours (Figures S35–S37). Surprisingly, the polysulfide changed colour in this experiment, from brown to grey (Figure 4 a). This result suggested that the polysulfide might self‐indicate when bound to a specific amount of Hg^2+^. Because this chromogenic response was only obvious above 5 mm HgCl_2_, it is unlikely to be useful in sensing low levels of Hg^2+^. However, it might be useful in monitoring the lifetime of a filter or other remediation device containing the polymer, where the colour change is observable after binding sufficient mercury.

After washing the Hg^2+^‐treated polymer extensively with water, SEM and EDS analysis of the surface indicated the presence of mercury‐rich nanoparticles (Figure S38–S39)—a result consistent with our previous studies on the interaction of Hg^2+^ with polysulfides.^22^ It was also encouraging to note that the mercury was strongly bound to the polymer and minimal leaching was observed when the polymer‐bound mercury was incubated in pure water. For example, after 1.0 g of the polysulfide captured 79 mg of HgCl_2_, the polymer was transferred to a 10 mL sample of milliQ‐purified water and incubated for 24 hours. The concentration of mercury in the water was measured by ICP‐MS to be 0.57 ppb, a level that is within regulatory limits for drinking water (Figure S40).^35^ Because Hg^2+^ is highly soluble in water, these low levels of leaching are a testament to the high affinity of the polymer to inorganic mercury.

The most prevalent form of mercury encountered in ASGM is mercury metal. It was therefore critical to assess how the polysulfides interacted with liquid mercury. In the first instance, 1.00 g of the canola oil polysulfide (50 % sulfur by weight) was added to a vial of water containing 100 mg of elemental mercury. The three‐phase mixture was stirred vigorously at room temperature. After 4 hours, no mercury was visible and the polymer had undergone a dramatic colour change from brown to black (Figure 4 a and Figure S41). After 24 hours of total treatment, the polymer was isolated by filtration, washed thoroughly with water and then dried to a constant mass of 1.099 g. By mass balance, this result indicated that 99 % of the mercury metal was captured by the polymer. EDS imaging (Figure 4 b and Figure S42) confirmed the surface of the polymer to be rich in mercury, as did Auger and XPS spectroscopic analysis (Figures S43–S44).

Characterisation by XRD revealed that the major product was metacinnabar, a form of mercury sulfide (Figure S45). Importantly, because metacinnabar is non‐toxic and insoluble in water, it has been proposed as a form in which mercury could be immobilised safely.^16^, ^36^ Additionally, the oxidation of mercury metal to metacinnabar provides an essentially non‐volatile form of mercury, thereby lowering the risk of inhalation and transmission of the pollution through air.^16^ Gratifyingly, the polysulfide prepared from used cooking oil behaved similarly in the capture of mercury metal, so there is no requirement to use pristine vegetable oils in the polysulfide synthesis (Figure S46).

It is important to note that the mechanism of mercury metal capture was distinct from that of HgCl_2_. In the case of liquid mercury metal (Hg^0^), the metal was oxidised by the polysulfide. The oxidant (S−S) could be derived either from free sulfur embedded in the polymer or the polysulfide cross‐links, as the amount of total mercury captured was correlated with total sulfur content (Figure S46). Because of this, factice containing as little as 1 % free sulfur by mass was also effective in capturing mercury metal, though a higher mass of total factice was required because of its lower total sulfur content (17 % total sulfur, Figure S46). For Hg^2+^, the sulfur of the polysulfide acted as a ligand to sequester the salt. In both cases, the final oxidation state of the mercury bound to the polysulfide was mercury(II). This result was consistent with XPS analysis in which the 4*f* photoelectron peak after capture of either HgCl_2_ or Hg^0^ had a binding energy consistent with that of a mercury(II) sulfide (Figure S44). At the same time, the structure of the mercury(II) product was different, as the HgCl_2_ presented as surface‐bound nanoparticles and the mercury metal was converted to metacinnabar. The greater sensitivity in the chromogenic response for mercury metal perhaps owed its origins to this structural difference. For instance, when 20 g of the polysulfide was exposed to 72 mg of mercury metal, the entire surface polymer sample appeared black (Figure S47). This result encourages future exploration of the canola oil polysulfide as a sensor for metallic mercury.

### Mercury capture from soil

Arguably the most challenging pollution to remedy in ASGM communities is mercury‐contaminated soil. When mercury metal is mixed with ore to form gold amalgams, the mercury is dispersed as microbeads that are covered with particles of soil and other debris. This soil‐bound mercury does not coalesce and, despite the high density of mercury, it can float on water. This so‐called “mercury flour” can be carried by waterways and threaten the environment and human health beyond the location of the mine.5a A simple and cost‐effective method for treating floured mercury is currently an outstanding problem for ASGM communities.^5^ We therefore turned to mercury‐contaminated soil and studied how the canola oil polysulfide might be used in its remediation.

**Figure 5:**
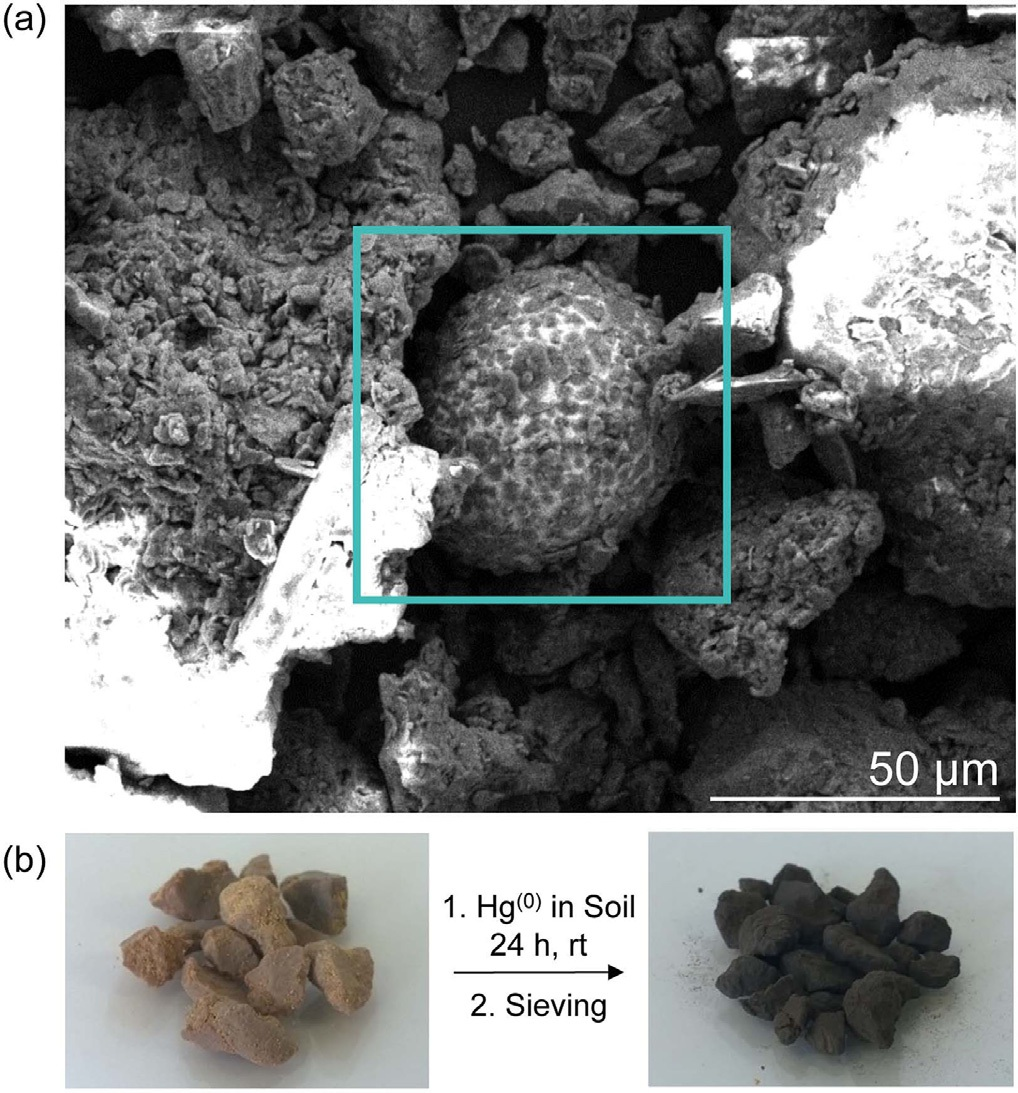
Remediation of simulated mercury flour. (a) SEM analysis of mercury flour showing a microbead of elemental mercury with soil particles bound to the surface. (b) Milling the simulated mercury flour with the canola oil polysulfide led to capture of the mercury. The polymer particles, bound to mercury, could be separated from the soil with sieves.

We first prepared mercury flour by using an end‐over‐end mixer to mill liquid mercury (200 mg) and 5 g fine loam comprised of soil particles less than 0.5 mm. While the characteristic silver coloured mercury was visible to the naked eye at the start of the mixing, it gradually dispersed into the soil as very fine beads over the course of several hours. After 24 hours, the mercury‐soil mixture was indistinguishable from the untreated soil (Figure S48). The floured mercury was analysed by SEM and EDS (Figure S49–S51), revealing microscale beads of mercury, with smaller soil particles adhered to the surface (Figure S50–S51). Figure 5 a shows a representative mercury bead, about 50 μm in diameter. To determine if the canola oil polysulfide could capture this floured mercury, the soil (5.0 g) was then treated with the canola oil polysulfide (5.0 g) containing 50 % sulfur by weight. Polymer particles of 2.5–5.0 mm were used so that they could be separated from the soil using a sieve. The solid mixture was milled using an end‐over‐end mixer. After 24 hours of treatment the polymer had clearly turned black (Figure 5 b), as observed in previous reactions with mercury metal. Separating the polymer from the soil using a sieve allowed analysis by EDS that verified mercury bound to the polymer (Figure S52–S53). Notably, the ability to isolate the polymer particles from soil provided a distinct advantage of the canola oil polysulfide over elemental sulfur. Additionally, while the amount of milling time and mass of polymer required for full remediation will need to be optimised for each type of soil and sediment, this initial demonstration of mercury removal from contaminated soil was an encouraging advance in dealing with mercury flour.

### Toxicity studies and prospects for in situ mercury remediation

In any remediation effort, the lifetime of the mercury‐binding material must be considered. Because of our interest in mercury pollution relevant to ASGM, we realised that the limited resources in these regions might prohibit separation of the polymer from soil and tailings post‐treatment. Furthermore, areas of contaminated soil can span several thousand acres,7d so complicated remediation protocols are simply not practical. We therefore considered whether in situ remediation or immobilisation would be appropriate—a practice where the polymer would be milled into the contaminated area and left in the environment after treatment.^9^ Decreased mobility of mercury and low‐toxicity would be required for this to be a viable strategy. The formation of metacinnabar in the reaction of mercury metal with the polymer was therefore encouraging, given its low propensity for leaching and low toxicity.^16^, ^36^ These properties notwithstanding, we thought it would be useful to carry out our own assessment of toxicity of the polymer and the polymer‐bound mercury.

**Figure 6:**
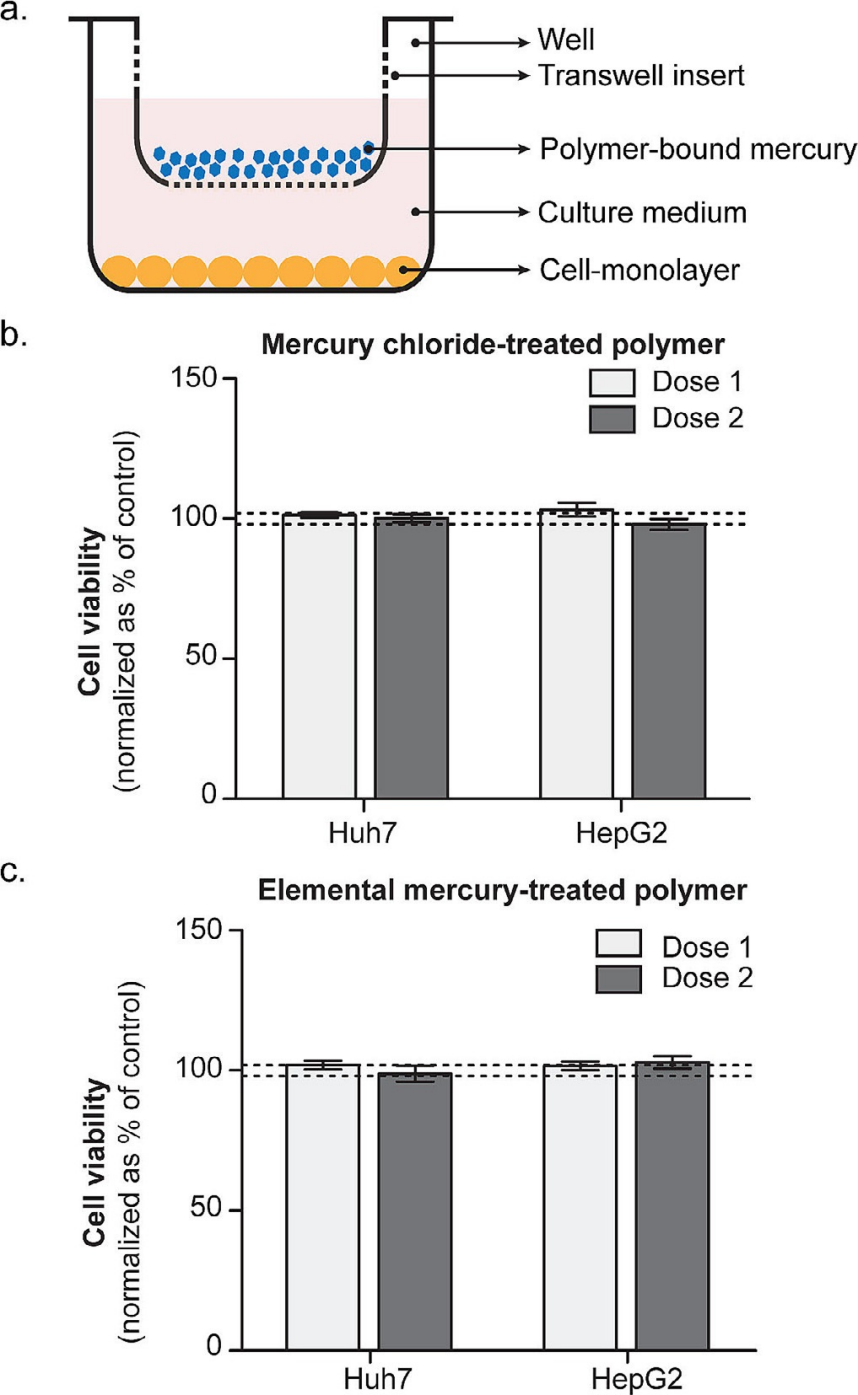
Toxicity assays of polysulfide after capturing mercury chloride or mercury metal. Cell viability was assessed using the CellTiter‐Blue Cell Viability Assay, and values obtained for cells exposed to mercury‐treated polymers were compared to values obtained for untreated polymers. (a) Cells were seeded in a 24‐well plate and the polymers were added to the bottom of a Transwell insert, submerged in the cell culture medium. (b) Cytotoxicity analysis for the mercury chloride‐treated polymer, in Huh7 and HepG2 cells. The polymer treated with HgCl_2_ contained 2.2 mg HgCl_2_ per gram of polymer. (c) Cytotoxicity for the elemental mercury‐treated polymer, in Huh7 and HepG2 cells. The polymer treated with Hg^0^ contained 79 mg mercury per gram of polymer. Bars represent average of biological triplicates, and error bars represent standard error of the mean. “Dose 1”: 3.75 mg polymer/ 300 μL of culture medium. “Dose 2”: 37.5 mg polymer/ 300 μL of culture medium. Under these conditions, no evidence of toxicity was revealed for any sample of the polymer‐bound mercury.

To assess toxicity, HepG2 and Huh7 human liver cells were cultured in the presence of both the unmodified canola oil polysulfide and the mercury treated polysulfide. In these experiments, the polymer samples were added to the permeable insert of Transwell cell culture plates. The insert effectively acted like a “teabag” where any mercury or other toxic materials leached into the growth media would be available to the cells (Figure 6 a). There was no difference in cell viability between the untreated cells and the cells treated with polymer, so the canola polysulfide itself exhibited no cytotoxicity in this assay (Figure S54). More impressively, neither the polysulfide used to capture HgCl_2_ nor the polysulfide used to capture mercury metal exhibited cytotoxicity in this experiment, as measured by cell viability (Figure 6 b–c and Figure S55). The polymer used to capture mercury chloride contained 2.2 mg of mercury per gram of polymer. The polymer used to capture mercury metal contained 79 mg of mercury per gram of polymer. Neither sample leached sufficient mercury to affect liver cell viability when 37.5 mg of polymer was added to the 300 μL well in the culture medium. In contrast, the addition of an aqueous solution of mercury chloride to the cells, in the absence of polymer, resulted in rapid cell death with and IC_50_ of 34 and 40 μm for Huh7 and HepG2 cells, respectively (Figure S56). For the polymer bearing captured mercury chloride, if all mercury were released into the growth medium, the concentration of mercury would be 1 mm Hg^2+^, more than 30 times the measured IC_50_ for HgCl_2_. For the polymer that oxidised and captured mercury metal, if all of this mercury were released into the growth medium, the concentration of mercury would be approximately 50 mm. Therefore, both mercury chloride and the oxidised mercury metal adhered to the polymer and were non‐toxic to the cells.

These results encourage consideration of the canola oil polysulfide as a material for in situ remediation where the polymer is mixed into mine tailings and contaminated soil to capture mercury and render it far less toxic, less volatile, and insoluble in water. We propose, in the first instance, that the product of this process could be left at the site of contamination. While ultimately mercury will need to be phased out in ASGM practice, and it is ideal to remove all mercury from the site of contamination, in situ remediation using the canola oil polysulfide is a relatively simple measure to address the extensive mercury pollution these communities face in the short‐term.

### Synthesis of a porous canola oil polysulfide

**Figure 7:**
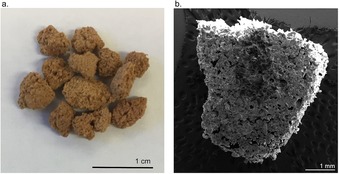
A porous version of the canola oil polysulfide. (a) Canola oil and sulfur were co‐polymerised in the presence of a sodium chloride porogen. Removing the sodium chloride was achieved by soaking the milled polymer in water. The product is a sponge‐like material. (b) SEM analysis of a cross‐section of a particle revealed the presence of pores and channels on the order of 100–200 microns in diameter.

The reaction of elemental mercury with the canola oil polysulfide was relatively slow, taking several hours in the experiments described in Figure 4 and Figure 5. For mercury vapour capture after coal combustion or during oil and natural gas refining, the process must be very rapid and continuous. We reasoned that increasing the surface area of the canola oil polysulfide would help the rate of mercury binding and reaction by increasing the amount of available sulfur. A porous version of the polysulfide was therefore prepared by synthesising the polymer in the presence of a sodium chloride porogen—a tactic inspired by a salt templating protocol recently reported by Hasell.^37^ In the synthesis, sulfur and canola oil were reacted directly as before and then sodium chloride (previously ground in a mortar and pestle) was added slowly to the reaction mixture. After reaching the gel point, the polymer–salt mixture was removed from the reaction vessel and milled into particles approximately 0.1–1.0 cm in diameter (Figure S57). These particles were then washed twice in water to leach the sodium chloride from the polymer. The resulting polymer—obtained in quantitative yield—was sponge‐like and contained micron‐scale pores and channels, as revealed by SEM analysis (Figure 7 and Figure S58). During the optimisation of this protocol, it was found that a large excess of sodium chloride was required (70 % of the total mass of the reaction mixture was sodium chloride). If less sodium chloride were used, substantial amounts of salt particles remain trapped in the polymer matrix. At the higher levels of sodium chloride, >99 % of the porogen can be leached from the polymer. The Raman spectrum (Figure S59) of the porous polysulfide was similar to the non‐porous polymer, as was the thermal stability and *T*_g_ (−12.9 °C) (see TGA and DSC analysis, Figure S60–S61). ^1^H NMR analysis of the CDCl_3_ soluble fraction of the polymer was also similar to the non‐porous variant (Figure S62). One notable difference in the porous polysulfide was absence of sulfur microparticles that were prominent in the non‐porous version. Though free sulfur was detected in the DSC analysis of the porous polymer (13 % by mass, Figure S60), the sodium chloride porogen apparently restricted the formation of larger sulfur particles.

### Removal of mercury from gas streams

**Figure 8:**
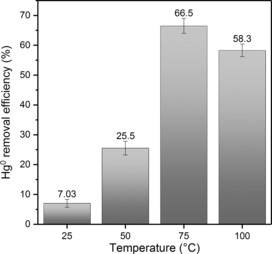
Mercury vapour capture using the porous canola oil polysulfide. 75 °C was found to be an optimal temperature for capturing mercury in a continuous process, with 67 % of the mercury removed from the gas stream over a residence time of approximately 0.24 seconds. The higher temperature increases the rate at which the polymer oxidises the mercury gas.

With a porous version of the canola oil polysulfide in hand, its ability to react with and capture elemental mercury gas was assessed. A 300 mg sample of the polymer was loaded in a quartz glass reactor, with the polymer occupying a volume of approximately 0.4 cm^3^. A stream of nitrogen containing mercury vapour was passed through the reactor, with the flow rate (0.1 L min^−1^) and level of mercury (586.4 μg Nm^−3^) precisely maintained using a mass flow controller (Figure S63). Mercury capture was determined by measuring the difference in the amount of mercury delivered to the reactor and that detected in downstream KMnO_4_ traps (Figure S63). At 25 °C, the polymer removed 7 % of the mercury from the gas stream. Reasoning that the reaction between the polysulfide and mercury would increase by heating the reactor, the experiment was repeated at 50, 75 and 100 °C (Figure 8 and Figure S64). Of these temperatures, 75 °C resulted in the highest mercury capture, enabling the canola oil polysulfide to react with and sequester 67 % of the mercury. This unoptimised mercury removal efficiency is quite remarkable considering the residence time for this experimental setup is a mere 0.24 seconds, a timeframe compatible for typical waste incineration and fossil fuel processing. This feasibility study should therefore encourage consideration of these polysulfides as inexpensive mercury sorbents for gas streams contaminated with mercury.^38^

### Removing mercury bound to organic matter (Hg‐NOM) from water

Mercury bound to natural organic matter (NOM) is often considered a recalcitrant form of pollution because humic matter, regularly containing thiols and sulfides, binds tightly to mercury. In natural and contaminated aquatic systems, mercury predominantly has an oxidation state of +2, but Hg^2+^ does not occur as a free, monatomic ion complexed only by water molecules. In freshwater streams and sediments, Hg^2+^ is typically bound by nucleophilic functional groups, which are present at high abundance in NOM. This complexation of mercury and methylmercury with NOM is known to affect its mobility, as well as chemical and biological transformation in aquatic environments.^25^

For the polysulfide polymer to capture this mercury, a ligand exchange would need to occur. In addition to testing the non‐porous and porous polysulfide for its ability to displace NOM, some of the porous polymer was partially reduced with sodium borohydride to install thiols that could perhaps facilitate this process and bind mercury (Figure S65). Testing this hypothesis, sorption isotherms for Hg(NO_3_)_2_ and a Hg‐NOM complex were determined at environmentally relevant mercury concentrations between 0.2 and 16 μg L^−1^. Over this concentration range, sorption of Hg(NO_3_)_2_ was found to follow a linear isotherm, confirming that in the absence of NOM all three forms of the polysulfide removed >90 % of the mercury in solution and the sorbent did not approach saturation or Hg binding capacity (Figure S66). By comparison, when mercury is associated with NOM (i.e., Hg‐NOM), functional groups on NOM compete with the polysulfide for mercury binding. Nevertheless, the removal efficiency at low Hg‐NOM concentrations for the porous and the reduced porous polysulfide reached 79 and 81 %, respectively (Figure S66). The removal efficiency of the non‐porous polysulfide, in contrast, was only 36 %.

As Hg‐NOM concentrations increased, the removal efficiency decreased, as indicated by a fit of the equilibrium data to the Langmuir sorption isotherm. The sorption capacity for the porous polysulfide reached a value of 1.11 μg‐Hg/g‐sorbent under the experimental conditions (Figure S66). The results clearly show that the porous polysulfide material can effectively outcompete NOM, particularly at concentrations typically encountered in mercury contaminated freshwater systems. Partial reduction of the polymer surface to install thiols had only a small impact on removal efficiency in the presence of Hg‐NOM and resulted in a lower sorption capacity compared to the porous polysulfide.

Additionally, we investigated whether sulfates were released from the porous polysulfide and its partially reduced derivative. Sulfate release from sulfur‐based sorbents may enhance mercury methylation by promoting sulfate‐reducing bacteria, which are considered the primary methylators in marine and estuarine environments.18b, 18c The assessment of sulfate release was accomplished in batch experiments by combining 30 mL of phosphate‐buffered Hg(NO_3_)_2_ or Hg‐NOM complex with 100 mg of the porous canola oil polysulfides followed by equilibration over 48 hours. The sulfate concentration in the filtered sample was then analysed by ion chromatography and normalised to the mass of the sample. The results indicated that sulfate release was typically below 100 μg g^−1^ and did not significantly elevate sulfate naturally present in the NOM used in the experiments (Figure S67). Therefore, the deployment of the polysulfide sorbent is not expected to enhance mercury methylation by stimulating sulfate reducing bacteria in the system.

### Sequestering an organomercury fungicide

Organomercury compounds have long been used as fungicides to protect grain seeds, sugarcane setts and other crops.^3^ While some of these fungicides have been restricted or banned, their continued use in both industrialised and developing nations is cause for concern.2a These mercury derivatives are highly toxic because they can be absorbed through the skin and enter and damage the central nervous system.1b These fungicides are known to compromise the health of marine life^39^ and accidental ingestion by humans has led to death, with the most infamous episode occurring in Iraq in 1971, where wheat seeds coated with mercury‐based fungicides were mistakenly consumed as food by thousands of people.^40^ Sorbents that are effective at capturing these fungicides could find use in preventing harmful runoff from fields to which they are applied. Accordingly, the porous canola oil polysulfide was tested in its ability to capture a representative mercury‐derived fungicide, 2‐methoxyethylmercury chloride (MEMC)‐a fungicide that is still used by sugarcane, rice and potato growers in several countries.^39^

**Figure 9:**
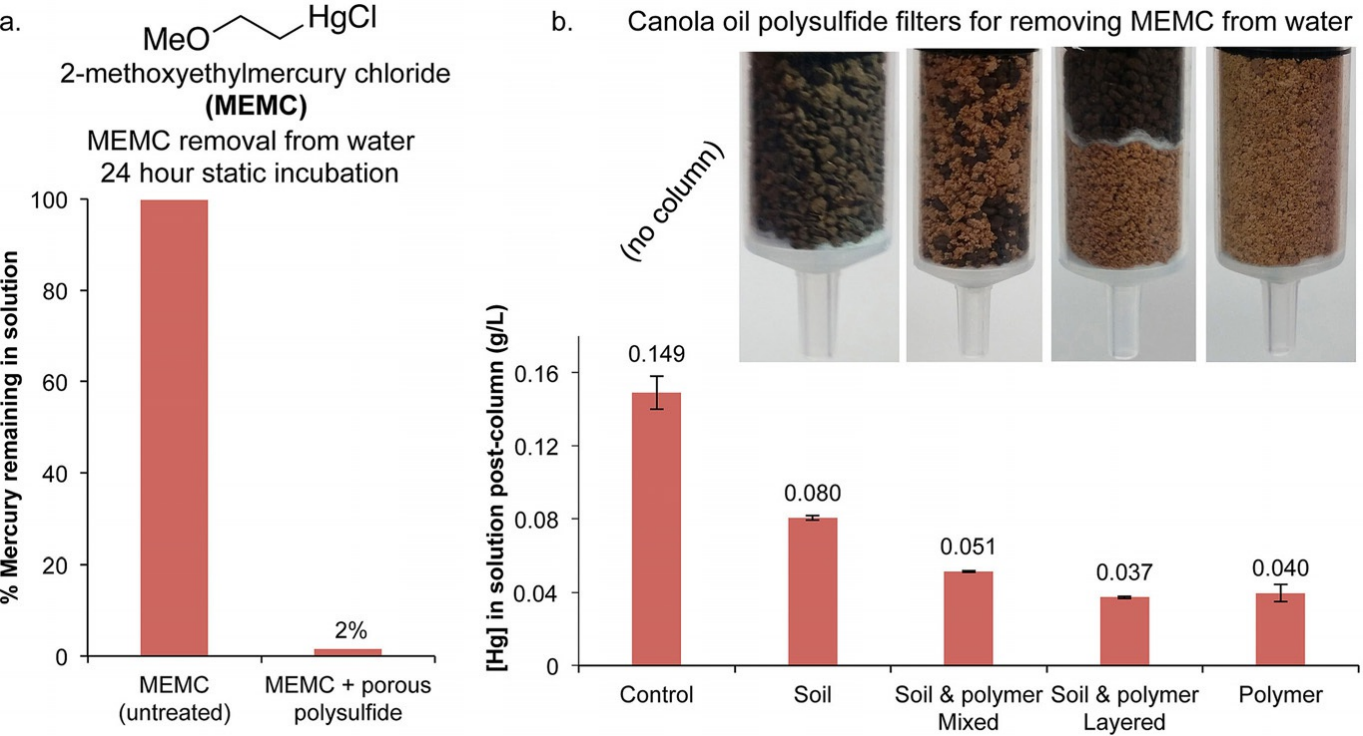
Trapping an organomercury fungicide, (2‐methoxyethylmercury chloride, MEMC), using the porous canola oil polysulfide. (a) Incubating a 0.15 g L^−1^ aqueous solution of MEMC with 2.0 g of the porous canola oil polysulfide for 24 hours resulted in the removal of 98 % of the mercury in solution. (b) Filters were constructed in the barrel of 10 mL syringes using soil (3.0 g), a random mixture of soil (1.5 g) and porous polysulfide (1.5 g), layers of soil (1.5 g) and polymer (1.5 g) separated by cotton, and solely porous polysulfide (3.0 g). Cotton plugs were used at the base of each column. Passing 3 mL of the MEMC solution (0.15 g L^−1^) resulted in reduction of mercury in the flowthrough. The soil layered on the polymer and the polymer alone were most effective, removing 75 and 73 % of the mercury, respectively.

To test whether the porous canola oil polysulfide could remove this compound from water, an aqueous solution of MEMC was prepared at 0.15 g L^−1^ (a typical operating concentration for the fungicide) and then 10 mL of this solution was incubated with 2.00 g of the porous polymer for 24 hours. After this time, the concentration of mercury was determined by ICP‐MS. Remarkably, 98 % of the mercury was removed from solution, whereas the mercury concentration did not change in solutions not treated with the polymer (Figure 9 and Figure S68). To determine if this remediation could be translated to a continuous process, a series of columns were prepared in which the porous polysulfide and soil were used as filtration media (Figure 9 and Figure S69). Next, 3 mL of the 0.15 g L^−1^ MEMC solution was passed through each column and the mercury concentration of the flowthrough was determined by ICP‐MS. Soil alone (3.0 g) retained 46 % of the mercury; soil and polymer (1.5 g each) mixed randomly together retained 66 % of the mercury; soil (1.5 g) layered on top of the polymer (1.5 g) retained 75 % of the mercury; and polymer alone (3.0 g) retained 73 % of the mercury. The total elution time for each column was approximately 2.5 minutes, so the mercury retention process is relatively fast. These results suggest the porous polysulfide might be useful as a soil additive that can reduce the levels of mercury‐based fungicides that leach into agricultural wastewater.

## Conclusion

Sulfur and unsaturated cooking oils were co‐polymerised to form a polysulfide rubber that captured mercury from air, water, and soil. Because sulfur is a by‐product of the petroleum industry and recycled cooking oil was a suitable starting material, the novel polymer can be made entirely from repurposed waste. This research is therefore an addition to the growing body of literature dedicated to preparing sulfur polymers with sustainable and low‐cost cross‐linkers.21d, 22, 29, 37, 41 The synthesis required a single, operationally simple chemical reaction. No purification was required and the transformation featured complete atom economy. A porous version of the material was also prepared using a sodium chloride porogen. The materials were demonstrated to be effective in capturing common forms of mercury pollution including liquid mercury metal, mercury vapour, inorganic mercury and organomercury compounds. The rapid reaction between the porous version of the polymer and mercury bode well for multiple industrial applications. The low‐cost will also motivate uptake in developing nations struggling to control mercury pollution associated with gold mining. Neither the polymer nor the mercury‐bound polymer were toxic to human cells, which prompts consideration of the polysulfide for in situ remediation of mine tailings, soil and agricultural wastewater. Currently, we are working with a variety of industrial partners, environmental agencies, and other non‐profit firms to deploy this technology at sites plagued with mercury pollution.

## Conflict of interest

Two authors (J.M.C. and M.J.H.W.) are inventors on a provisional patent application filed in Australia on April 20, 2016 (Application number 2016901470). Among the claims in this patent application is the preparation of polysulfides from sulfur and vegetable oils and their use in mercury capture. The canola oil polysulfide featured in this study is licensed for sale by Kerafast.

## Supporting information

As a service to our authors and readers, this journal provides supporting information supplied by the authors. Such materials are peer reviewed and may be re‐organized for online delivery, but are not copy‐edited or typeset. Technical support issues arising from supporting information (other than missing files) should be addressed to the authors.

Supplementary
