# Supplementary material for: Laying Waste to Mercury: Inexpensive Sorbents Made from Sulfur and Recycled Cooking Oils
Source: Chemistry. 2017 Aug 30;23(64):16219–30. doi: 10.1002/chem.201702871 (PMC5724514; doi:10.1002/chem.201702871)
Supplement: Supplementary file 1 — Supplementary [file CHEM-23-16219-s001.pdf]

CHEMISTRY
A European Journal

Supporting Information

Laying Waste to Mercury: Inexpensive Sorbents Made from Sulfur and Recycled Cooking Oils

Max J. H. Worthington,[a, b] Renata L. Kucera,[a] Inês S. Albuquerque,[c] Christopher T. Gibson,[a, b] Alexander Sibley,[a, b] Ashley D. Slattery,[a, b] Jonathan A. Campbell,[a, b] Salah F. K. Alboaiji,[a, b] Katherine A. Muller,[d] Jason Young,[a, e] Nick Adamson,[a, b, h] Jason R. Gascooke,[a, b] Deshetti Jampaiah,[f] Ylias M. Sabri,[f] Suresh K. Bhargava,[f] Samuel J. Ippolito,[f, g] David A. Lewis,[a, b] Jamie S. Quinton,[a, b] Amanda V. Ellis,[a, b, h] Alexander Johs,[d] Gonçalo J. L. Bernardes,[c, i] and Justin M. Chalker*[a, b]

chem_201702871_sm_miscellaneous_information.pdf

Laying Waste to Mercury: Inexpensive Sorbents Made from Sulfur and Recycled Cooking Oils

Max J. H. Worthington,[1,2] Renata L. Kucera,[1] Inês S. Albuquerque,[3] Christopher T. Gibson,[1,2] Alexander Sibley,[1,2] Ashley D. Slattery,[1,2] Jonathan A. Campbell,[1,2] Salah F. K. Alboaiji,[1,2] Katherine A. Muller,[4] Jason Young,[1,5] Nick Adamson,[1] Jason R. Gascooke,[1,2] Deshetti Jampaiah,[6] Ylias M. Sabri,[6] Suresh K. Bhargava,[6] Samuel J. Ippolito,[6,7] David A. Lewis,[1,2] Jamie S. Quinton,[1,2] Amanda V. Ellis,[1,2,8] Alexander Johs,[4] Gonçalo J. L. Bernardes,[3,9] Justin M. Chalker[1,2]*

To whom correspondence should be addressed: justin.chalker@flinders.edu.au

Affiliations:

1. School of Chemical and Physical Sciences, Flinders University, Bedford Park, South Australia, Australia

2. Centre for NanoScale Science and Technology, Flinders University, Bedford Park, South Australia, Australia

3. Instituto de Medicina Molecular, Faculdade de Medicina da Universidade de Lisboa, Lisbon, Portugal

4. Environmental Sciences Division, Oak Ridge National Laboratory, Oak Ridge, Tennessee, USA

5. Flinders Analytical, School of Chemical and Physical Sciences, Flinders University Bedford Park, South Australia, Australia

6. Centre for Advanced materials & Industrial chemistry (CAMIC), School of Applied Sciences, RMIT University, Melbourne, Victoria, Australia

7. School of Electrical and Computer Engineering, RMIT University, Melbourne, Victoria, Australia

8. School of Chemical and Biomedical Engineering, University of Melbourne, Parkville, Victoria, Australia

9. Department of Chemistry, University of Cambridge, Cambridge, United Kingdom

Table of Contents **Page Number**

General Experimental Considerations	S3
Synthesis of canola oil polysulfide (50% sulfur)	S5
Synthesis of polysulfide from olive oil and sunflower oil	S6
Lipid analysis of oils	S7
Synthesis of canola oil polysulfide with varying sulfur content	S12
Synthesis of canola oil polysulfide using recycled cooking oil	S13
IR analysis of canola oil polysulfide	S14
NMR analysis of $CDCl_3$ soluble fraction of non-porous canola oil polysulfide (50% sulfur)	S15
Reaction of sulfur and fatty acid methyl esters derived from plant oils	S16
SEM analysis of canola oil polysulfide (50% sulfur)	S18
EDS analysis of canola oil polysulfide (50% sulfur)	S19
Auger analysis of canola oil polysulfide (50% sulfur)	S20
Raman spectroscopic analysis of canola oil polysulfide (50% sulfur)	S21
Ellman's test on the canola oil polysulfide	S23
TGA of canola oil polysulfide (30, 50, 60 and 70% sulfur)	S24
Simultaneous thermal analysis (DSC and TGA) of unreacted vegetable oils	S25
DSC of non-porous canola oil polysulfides and free sulfur analysis	S27
Comparison of polysulfides prepared from canola oil, olive oil, and sunflower oil (TGA and DSC)	S30
Comparison of canola oil polysulfide to commercial factice (IR, Raman, TGA, DSC)	S32
Comparison of canola oil polysulfide prepared by inverse and classical vulcanisation (TGA and DSC)	S35
Dynamic mechanical analysis of canola oil polysulfide	S37
Glass transition temperature by DSC for non-porous canola oil polysulfide (50% sulfur)	S38
Mercury chloride capture from water (including SEM and EDS analysis of polymer)	S39
Leaching of mercury(II) from canola oil polysulfide	S43
Mercury metal capture from water (including SEM, EDS, and Auger analysis of polymer)	S44
XPS analysis of canola oil polysulfide before and after mercury capture	S47
XRD analysis of canola oil polysulfide before and after mercury metal capture	S48
Mercury capture using a polysulfide prepared from recycled cooking oil	S49
Mercury capture using commercial factice F17	S49
Chromogenic response of canola oil polysulfide after reaction with mercury metal	S50
Mercury flour preparation and SEM and EDS analysis	S51
Capturing mercury flour with the non-porous canola oil polysulfide	S55
Toxicity studies	S57
Synthesis of a porous canola oil polysulfide (50% sulfur) using a sodium chloride porogen	S59
SEM analysis of porous canola oil polysulfide (50% sulfur)	S60
Raman spectroscopic analysis of porous canola oil polysulfide (50% sulfur)	S61
TGA and DSC of porous canola oil polysulfide (50% sulfur)	S62
Glass transition temperature by DSC for porous canola oil polysulfide (50% sulfur)	S63
NMR analysis of $CDCl_3$ soluble fraction of porous canola oil polysulfide (50% sulfur)	S64
Mercury vapour experiments using the porous canola oil polysulfide	S65
Installation of thiols on the porous canola oil polysulfide by partial reduction with $NaBH_4$	S66
Experiments on mercury bound to natural organic matter (NOM)	S67
Sulfate release from the porous canola oil polysulfide	S69
Experiments using an organomercury fungicide [2-methoxyethylmercury chloride (MEMC)]	S70
References	S72

General Experimental Considerations

IR Spectroscopy: Infrared (IR) spectra were recorded on a Fourier Transform spectrophotometer using the ATR method. Absorption maxima (υ_{max}) are reported in wavenumbers (cm^{-1}).

NMR Spectroscopy: Proton nuclear magnetic resonance (^1H NMR) were recorded on a 600 MHz spectrometer. All chemical shifts are quoted on the δ scale in ppm using residual solvent as the internal standard (^1H NMR: $CDCl_3$ δ = 7.26).

GC-MS: Gas chromatography-mass spectrometry (GC-MS) was carried out on a Varian CP-3800 using a Phenomonex Zebron ZB5MS, 5 %-phenyl-Arylene-95 %-dimethylpolysiloxane column (30 m long × 25 mm film thickness × 0.25 mm ID). The injection temperature was set at 220 ºC, the column temperature at 190 ºC, and the gas flow rate 1.2 mL/min. Electron ionization was used to obtained nominal masses.

Raman Spectroscopy and Microscopy: Raman spectra were acquired using a Witec alpha300R Raman microscope at an excitation laser wavelength of 532 nm with a 40X objective (numerical aperture 0.60). Typical integration times for single Raman spectra were between 20 to 60 s and averaged from 1 to 3 repetitions. Confocal Raman images were also acquired with integrations between 1 to 6 seconds per pixel. Each pixel in the Raman images represents a Raman spectrum with the number of pixels in a typical Raman image representing hundreds to thousands of spectra. Confocal Raman images are generated by plotting the intensity of a specified region of each Raman spectrum that corresponds to a material, versus the X-Y position of the excitation laser as it scans the sample surface.

Raman data were also obtained using an XplorRA Horiba Scientific Confocal Raman microscope. Spectra were acquired using a 50X objective (numerical aperture 0.6) at an excitation wavelength of 532 nm. Typical integrations times for the spectra were 20 to 60 s and averaged from 1 to 3 repetitions.

SEM and EDS: Scanning Electron Microscopy (SEM) images were obtained using an FEI F50 Inspect system, while corresponding EDS spectra were obtained using an EDAX Octane Pro detector.

XPS: X-Ray Photoelectron Spectroscopy was performed on a Leybold Heraeus LHS-10 with a SPECS XR-50 dual anode source operating at 250 W. Base vacuum pressure in the analysis chamber was better than $5x10^{-9}$ torr. All spectra were taken with the 1253.6 eV Mg-Kα anode with the analyser pass energy set to 20 eV. Survey spectra were taken 'constant retarding ratio mode', while high resolution spectra were taken in fixed analyser transmission mode.

Auger Spectroscopy: Scanning Auger Electron Spectromicroscopy was performed on a PHI710 Scanning Auger Nanoprobe. Samples were sputter coated with 2 nm of Platinum prior to analysis. The vacuum pressure in the analysis chamber during analysis was maintained below 10^{-9} Torr. Electron beam energies used for analysis ranged between 3 kV and 10 kV, with a beam current of between 3 and 10 nA.

Dynamic Mechanical Analysis: Dynamic Mechanical Analysis was performed on a TA Q800 DMA in tension mode. Samples were prepared as short bars with dimensions of 1.4 cm × 0.8 cm × 0.2 cm. The sample was cooled to -100 ºC and then heated to 170 ºC at 3.0 ºC/min

Differential Scanning Calorimetry (DSC): Differential Scanning Calorimetry (DSC) was carried out using a Perkin Elmer DSC 8000 with nitrogen furnace purged at 20 mL/min. Samples were approximately 7 mg and sealed in aluminium sample pans. The sample was cooled to -80 ºC, held for 5 minutes, and then heated to 300 ºC at 10 ºC/min.

Thermogravimetric Analysis (TGA): Simultaneous Thermal Analysis (STA) was carried out on a Perkin Elmer STA8000 simultaneous thermal analyzer (STA). A sample size between 11 and 15 mg was used in each run. The furnace was purged at 20 mL/min with nitrogen, and equilibrated for 1 minute at 30 ºC before each run. Heating was carried out up to 700 ºC using a 20 ºC/min heating rate. The temperature was held isothermally at 700 ºC at the end of each experiment to oxidize remaining organic matter.

X-ray diffraction: Powder X-ray diffraction (XRD) patterns were recorded on a Bruker D8 Advance Eco diffractometer (Bragg-Brentano geometry) using Co-Kα radiation (λ = 1.78897 Å). The Bragg angle (2θ) was varied from 15° to 90° with a step size of 0.019°, measurement time of 0.45 s per step and sample rotation at 15 rpm. The XRD patterns were collected on a silicon low background sample holder, where powder samples were deposited onto the surface of the holder and spread evenly using a drop of acetone.

ICP-MS: ICPMS data was acquired on a Perkin Elmer NexION 350D ICPMS with a quartz cyclonic spraychamber controller at 2 °C. The sample introduction system consisted of a 0.76 mm ID tube, meeting at a mixing junction with a 0.19 mm ID tube carrying an Indium internal standard. Sample injection rate 0.45 mL/min sample and 0.03 mL/min internal standard. RF power 1600 W, plasma flow 16 L/min, auxiliary flow 1.2 L/min, nebuliser flow 0.9 L/min, dwell time 100 ms, CeO+/Ce+ <2%. The instrument was run in KED mode (He flow 4.7 mL/min) to minimise interference from polyatomic molecules. Before use the ICPMS was tuned with a 1 ppb tuning solution (Be, Ce, Fe, In, Mg, Pb, U - part number N8145051) to within Perkin Elmer specifications. Calibration curves for mercury were carried out for each measurement using dilutions from a stock solution of 1,000 ppm Hg in 2% HNO_3 (Chem-Supply, South Australia). Internal standards and recovery spikes were used for quality control in all measurements.

Canola Oil Polysulfide Synthesis (50% sulfur, 50% canola oil, 40 g scale)
Sulfur (technical grade, 20.0 g) was added to a 250 mL round bottom flask and then melted, with stirring, before heating further to 180 °C. Canola oil (20.0 g) was then added dropwise over 3-5 minutes, resulting in a two-phase mixture. The reaction was stirred vigorously to ensure efficient mixing of the two phases. The mixture appeared to form one phase after approximately 10 minutes. Heating was continued for an additional 10 minutes at 180 °C. Over this time, the product formed a rubbery solid. The material was then removed from the flask and then blended for 3 minutes (8.5 cm rotating blade) to provide rubber particles ranging in size from 0.2 to 12 mm in diameter with an average diameter of 2 mm. The particles were then transferred to a 250 mL beaker and treated with enough 0.1 M NaOH to cover the particles entirely (~60 mL). This mixture was stirred for 90 minutes at room temperature to remove residual hydrogen sulfide. After this time, the particles were isolated by filtration and then washed on the filter with deionised water (3 × ~50 mL). The particles were then collected from the filter and air dried at room temperature and pressure for 24 hours. Typically, this procedure provided a final mass between 39.2 and 40.0 g of the washed and dried canola oil polysulfide particles (>98% yield). The small loss in yield is attributed to unrecovered solid polymer during removal from the reaction vessel and filtration units.

Simplified structure of the canola oil and the canola oil polysulfide. Note that the polysulfide can potentially crosslink inter- and intramolecularly.

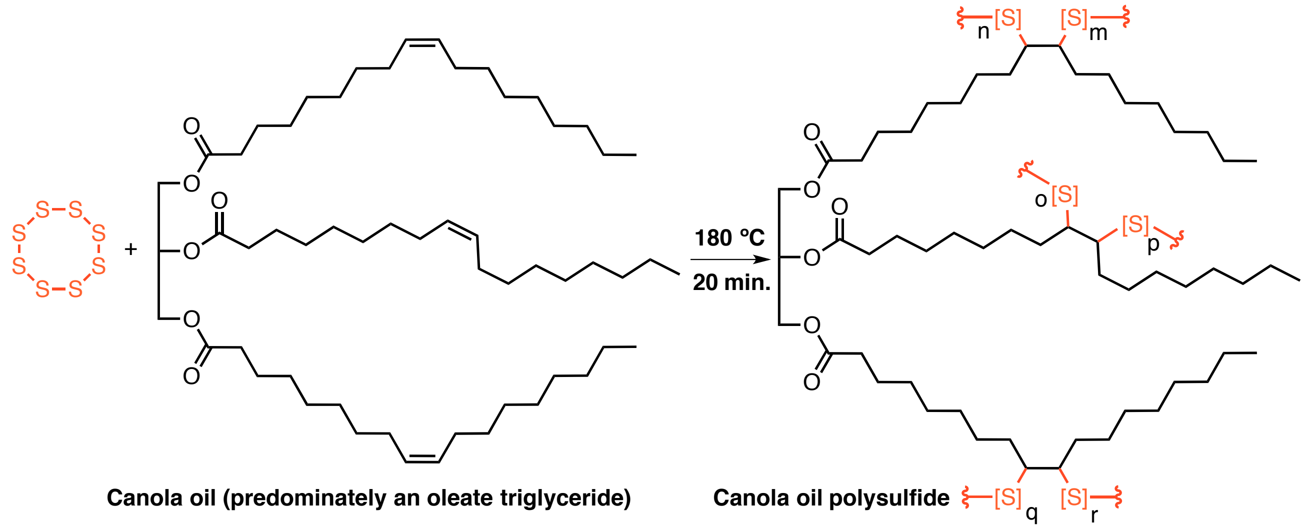

Fig. S1 | Synthesis of the canola oil polysulfide

Polysulfide prepared from sunflower oil and from olive oil

Sunflower and olive oil polysulfides were prepared using the same procedure as to prepare canola oil polysulfide. Sulfur (20.0 g) was added to a 250 mL round bottom flask and heated, with stirring, to 180 °C. After 5 minutes of heating at this temperature the sulfur turned from a yellow to an orange liquid. At this point, the sunflower or olive oil (20.0 g) was added dropwise over 5 minutes. After 12 minutes, the reaction with sunflower oil reached its gel point and formed a rubber. The reaction with the olive oil reached its gel point after 21 minutes of reaction time. Both samples were left to cool for 15 minutes before removing from their flasks. A third reaction prepared with canola oil was carried out for comparison. All samples were independently washed by submerging in 0.1 M aqueous NaOH for 90 minutes followed by washing with DI water and drying in open air. The samples have the same physical appearance:

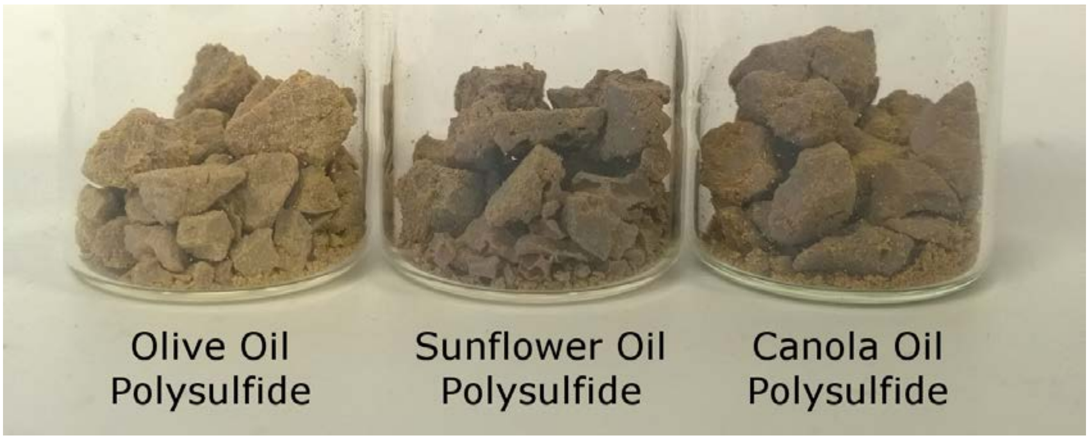

Fig S2 | A polysulfide rubber is obtained by the reaction of an equal mass of sulfur and olive oil, sunflower oil, or canola oil. The time to reach the gel point is shorter for sunflower oil, likely because of its higher polyunsaturated linoleic acid content in the triglyceride.

Lipid analysis of vegetable oils

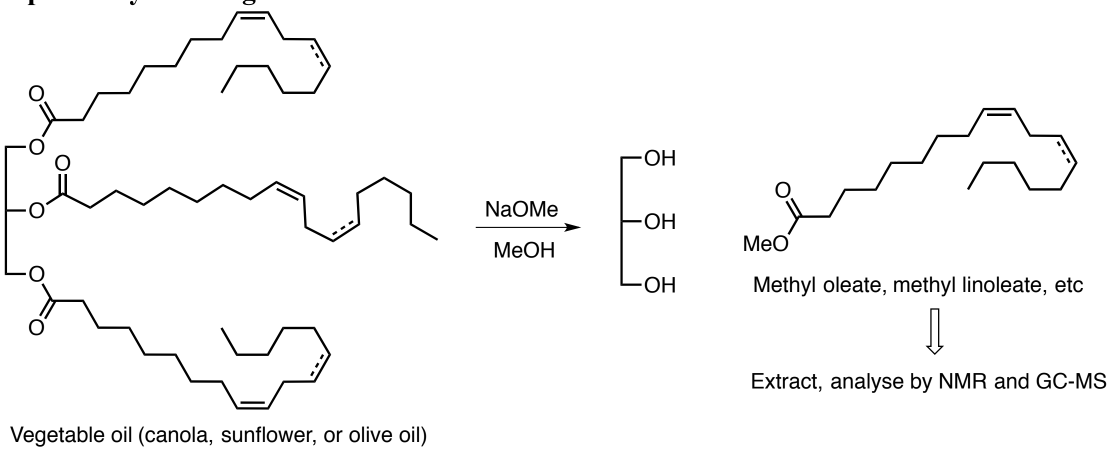

Vegetable oil (1.00 g) was mixed with methanol (100 mL) in a 250 mL round bottom flask and cooled to 0 °C. Sodium methoxide (100 mg) was then added to the stirred mixture. The reaction mixture was stoppered and stirred vigorously at room temperature for 24 hours. Vigorous stirring is important to ensure effective mixing of the two phases present at the start of the reaction. After 24 hours, the reaction was cooled to 0 °C and quenched with 0.1 M HCl (10 mL). The mixture was transferred to a separatory funnel and then diluted with ethyl acetate (150 mL) and water (150 mL). The organic layer was isolated and then washed with water (3 x 50 mL) and brine (3 x 50 mL) before drying (sodium sulfate), filtering and concentrating under reduced pressure. Analysis by ^{1}H NMR and GC-MS revealed clean conversion to the fatty acid methyl esters.
Typical yields for fatty acid methyl esters from 1.00 g vegetable oil:
Canola oil: 800 mg   Sunflower oil: 800 mg   Olive oil: 780 mg

General assignments for the ^{1}H NMR for the mixture of fatty acid methyl esters. Note that the relative integration will depend on the degree of unsaturation.

(600 MHz, CDCl3): 0.85-0.88 (t, J = 7.0, $CH_2C\underline{H}_3$), 1.25-1.35 (m, -($C\underline{H}_2$)-, non-allylic/non-alpha), 1.60 (m, $COCH_2C\underline{H}_2$) 1.99-2.05 (m, $C\underline{H}_2CH{=}CHC\underline{H}_2$) 2.28-2.31 (t, J = 7.6, $COC\underline{H}_2$), 3.65 (s, $CO_2C\underline{H}_3$), 5.32-5.36 (m, $C\underline{H}{=}C\underline{H}$)

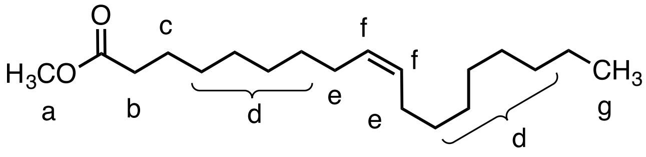

methyl oleate

^{1}H NMR Spectrum of fatty acid methyl ester obtained from canola oil (primarily methyl oleate):

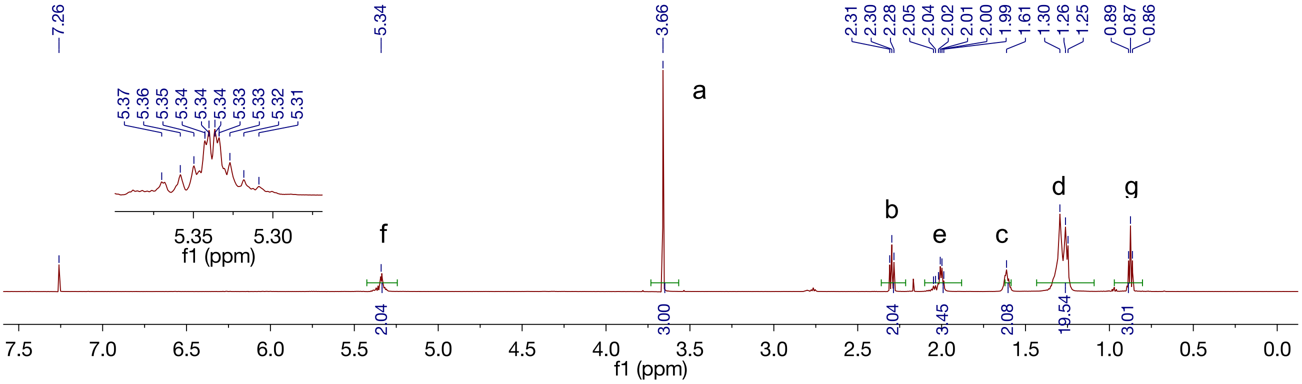

^{1}H NMR Spectrum of fatty acid methyl ester obtained from sunflower oil (primarily a mixture of methyl linoleate and methyl oleate):

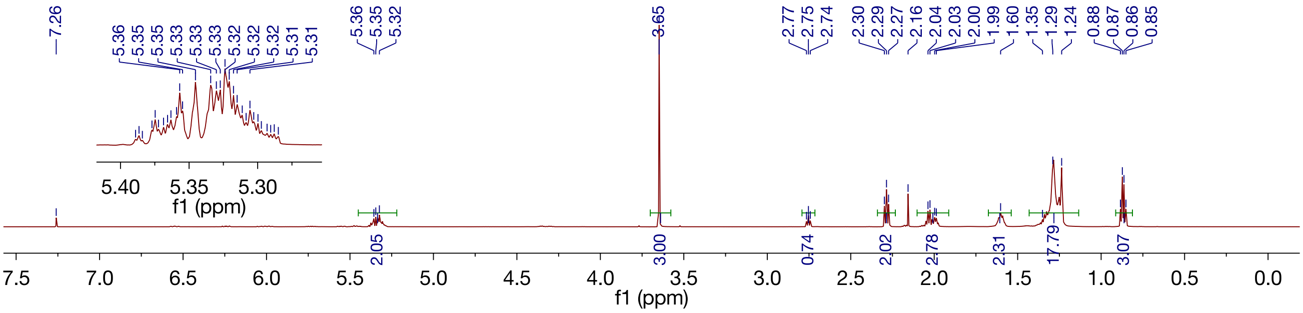

^{1}H NMR Spectrum fatty acid methyl ester obtained from olive oil (primarily methyl oleate):

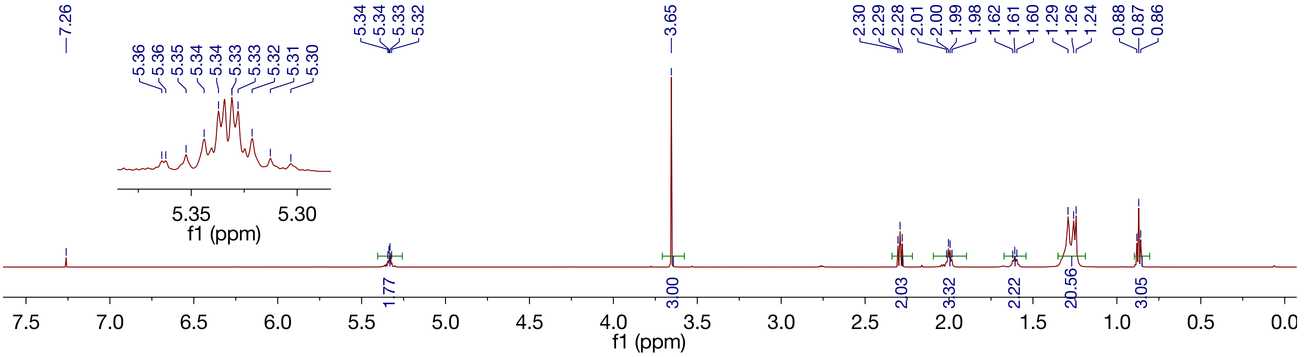

Fig. S3 | ^{1}H NMR (600 MHz, $CDCl_3$) of fatty acid methyl esters derived from canola oil (top), sunflower oil (middle) and olive oil (bottom). The alkene region is expanded, showing differences in unsaturation. Assignments are provided for methyl oleate derived from canola oil for reference.

Method for GC-MS analysis

Fatty acid methyl esters prepared from the oils as described in the previous experiment were prepared as a solution in chloroform (~5 mg/mL) and then analysed by GC-MS using the following method on a Varian CP3800: Hold at 50 ºC for 1 min, ramp from 50 to 200 ºC at a rate of 25 ºC /min. Slow ramp to 3 ºC/min rate from 200 to 230 ºC and hold at 230 ºC for 25 min. Next ramp from 230 ºC to 280 ºC at 25 ºC /min and hold at 280 ºC for 10 min. The total run time: 54 minutes. Injection temp: 250 ºC, carrier gas flow rate 1.2 mL/min. Representative GC traces are shown below with the major fatty acid methyl esters labelled. Methyl ester molecular ions were identified by comparison to the major fragmentation product, $[M-31]^+$, due to a loss of the methoxy group.

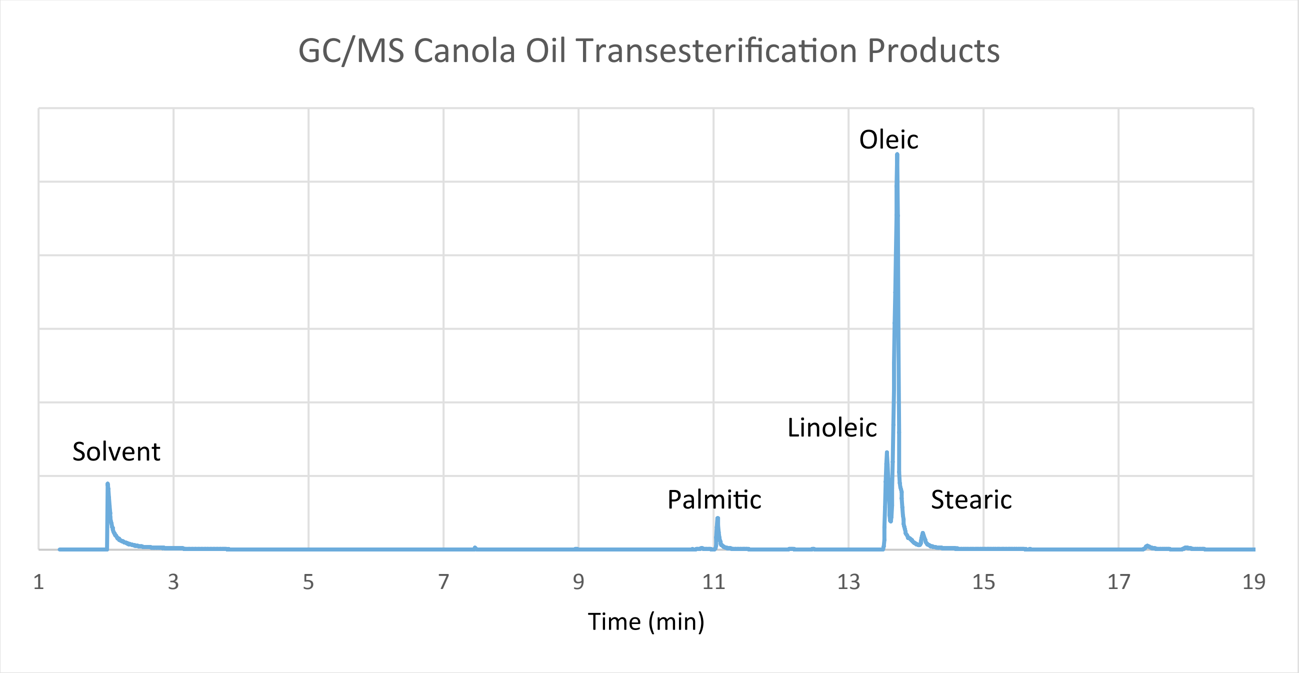

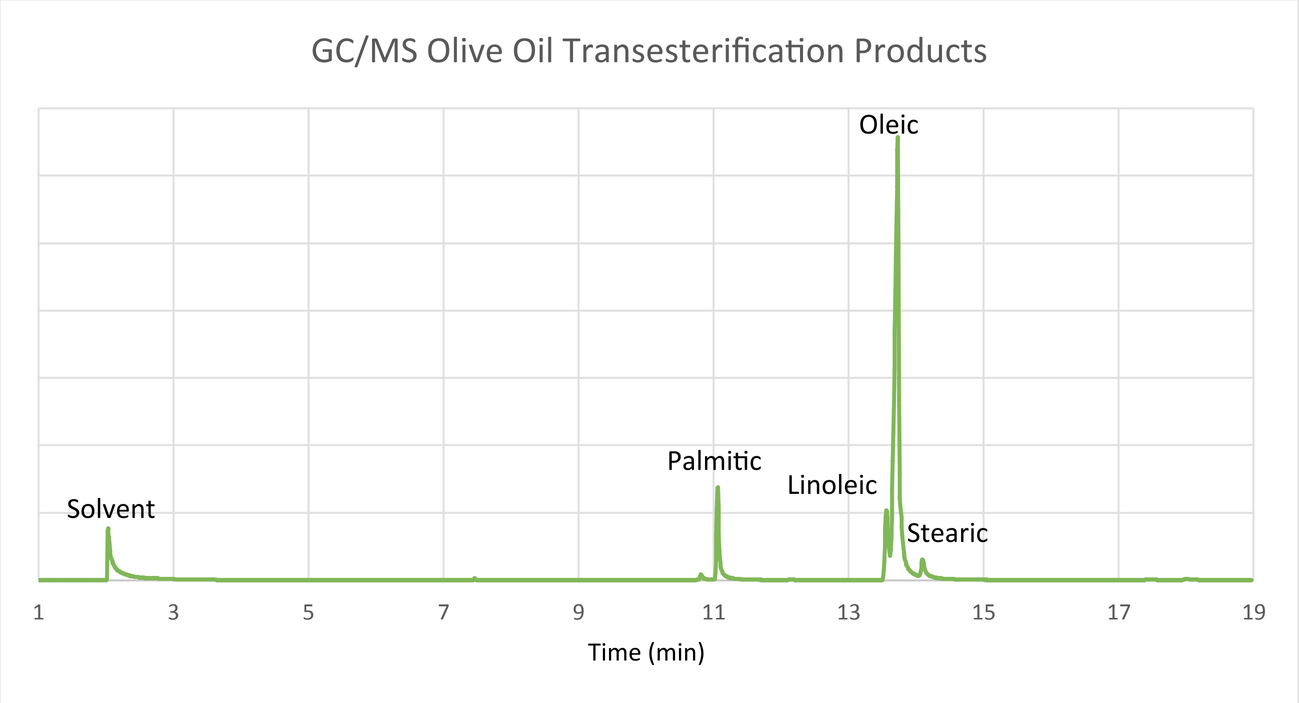

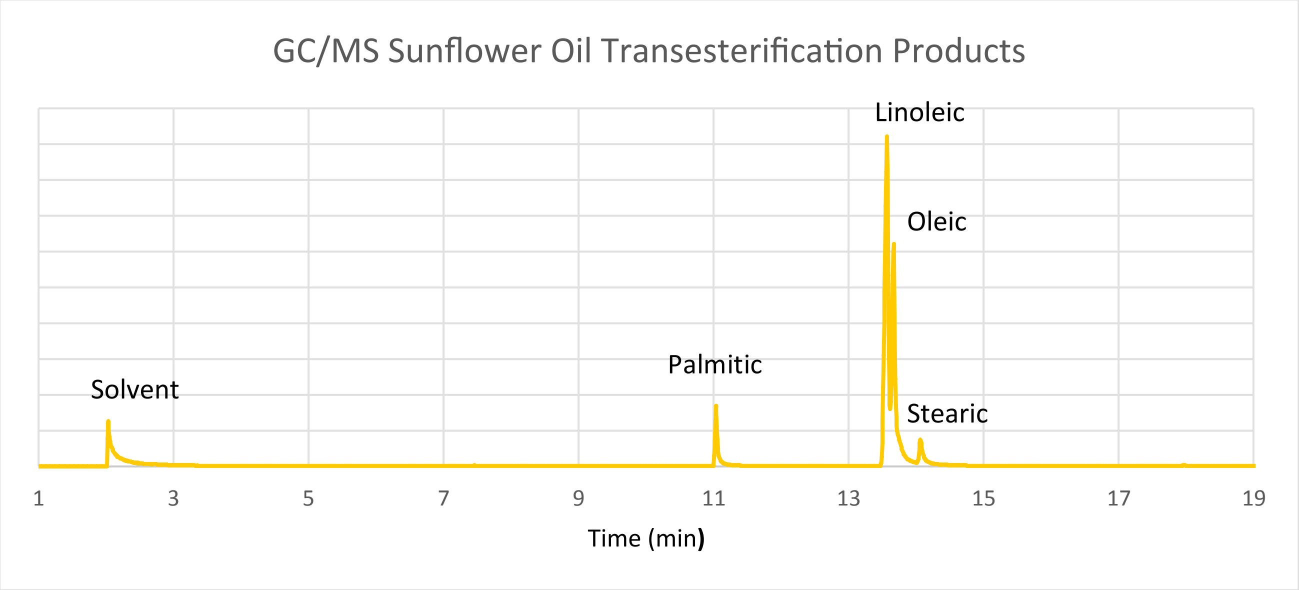

Fig. S4 | Representative GC-MS data for lipid analysis of canola oil, olive oil, and sunflower oil, with tabulated data shown below.

Compound (Fatty acid methyl ester)	Canola Oil (% content)	Olive Oil (% content)	Sunflower Oil (% content)
oleic	78.7	77.7	37.3
linoleic	14.2	8.91	50.0
palmitic	4.01	9.89	0.064
stearic	1.82	2.26	5.40
paullinic	0.66	0	0
palmitoleic	0	0.63	0
arachidic	0	0.31	0.14
linolenic	0	0.16	0
myristic	0.036	0	0.040
margaric	0.028	0	0
Not identified	0.546	0.14	7.056
Total	100%	100%	100%

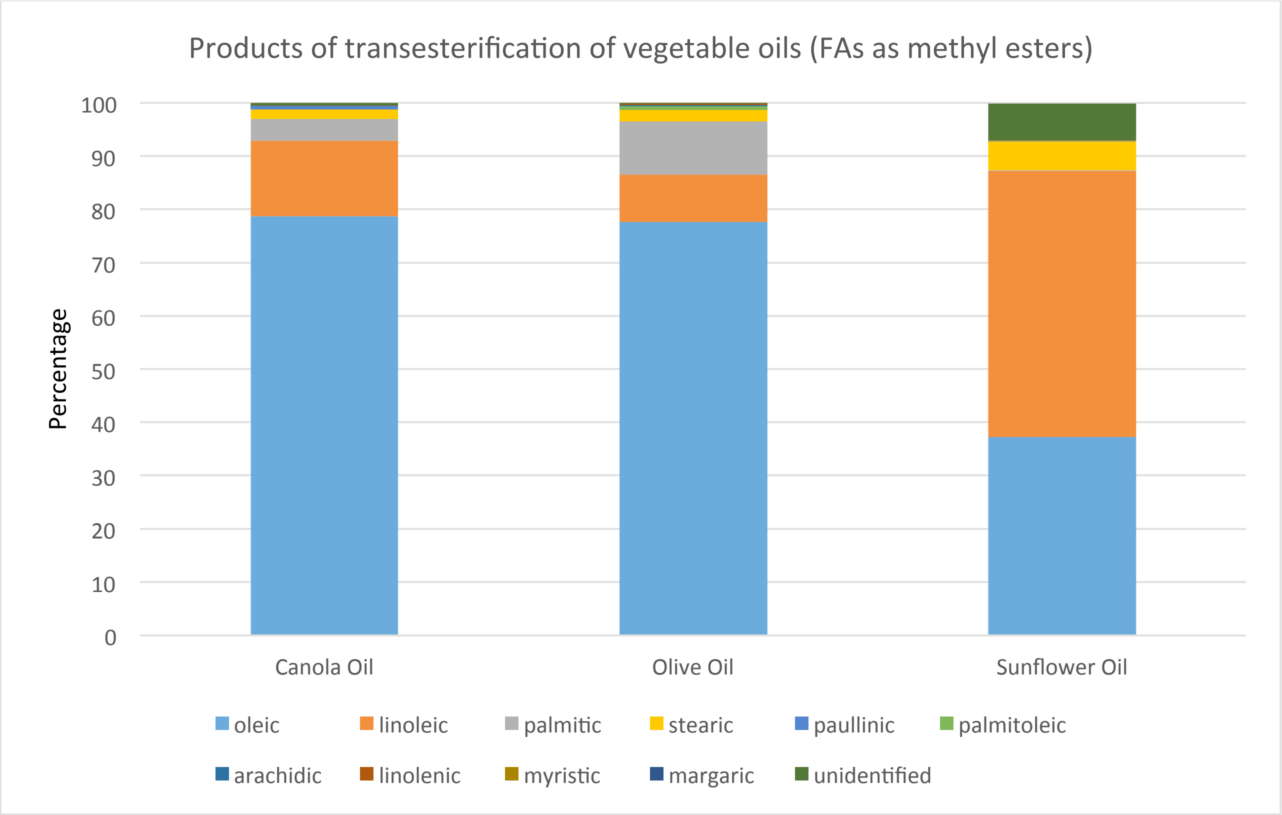

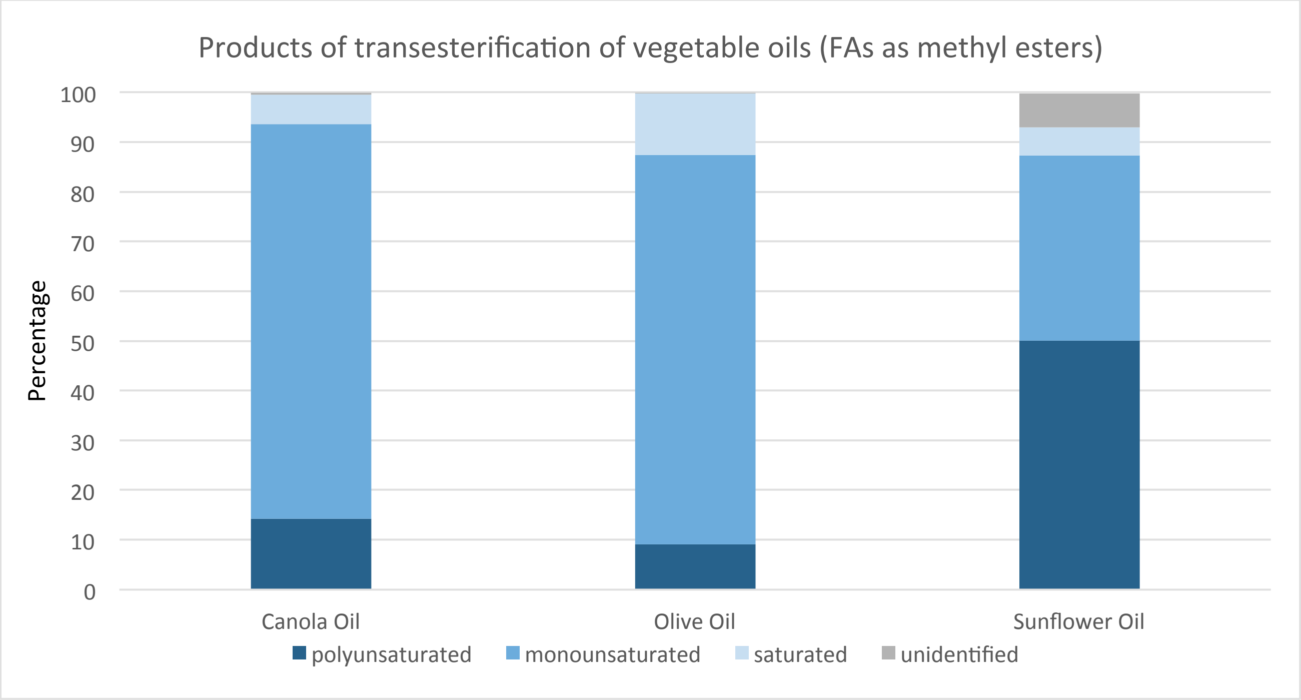

Fig. S5 | Summary of lipid analysis: The major conclusion is that the polysulfide is predominately formed by reaction with the alkenes from oleate and linoleate esters in the triglyceride.

Preparing the Canola Oil Polysulfide at Different Sulfur Compositions

Canola oil polysulfides were prepared with different sulfur content by varying the ratio of canola oil to sulfur used in the synthesis. In a typical synthesis, sulfur was heated to 180 ºC and the corresponding mass of sulfur was added slowly to maintain a constant internal temperature. All reactions were carried out on a 40 g scale. The two phase mixture was stirred rapidly to ensure efficient mixing. Typically, all samples reached the gel point within 20 minutes. Even prolonged heating (50 minutes) of the reaction mixture containing 10% sulfur did not result in a rubber.

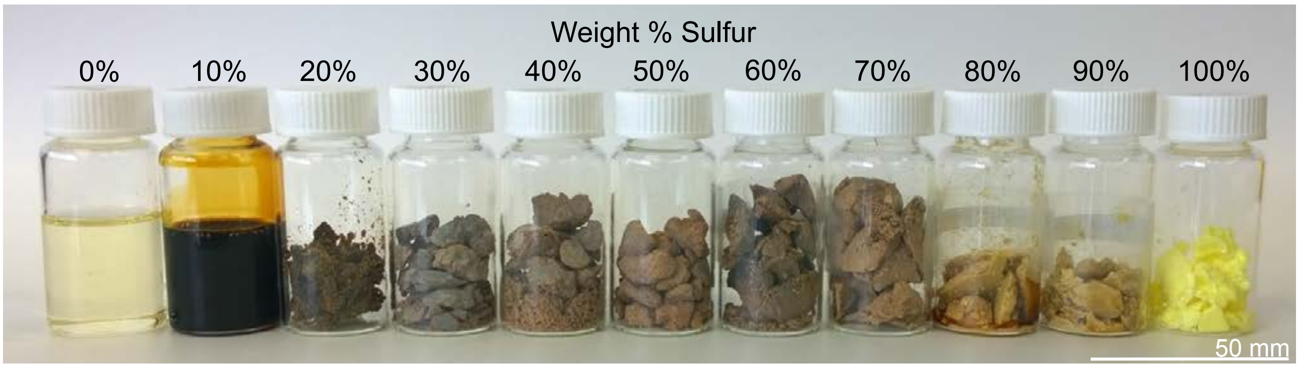

Fig. S6 | The reaction of canola oil with sulfur at different mass ratios. At 10% sulfur, a liquid was obtained. Between 20% and 70% sulfur, the product was a rubber. At 80% sulfur and higher, the product was brittle.

Synthesis of polysulfide using recycled cooking oil

Used cooking oil was obtained from a local café after it had been used to fry various foods for one day. The oil was used as received and was not purified in any way. In the synthesis of the polysulfide, sulfur (10.0 g) was added to a 250 mL round bottom flask and heated, with stirring, to 180 ºC. 5 minutes after reaching this temperature, the sulfur turned from a yellow to orange liquid. At this point, the crude, recycled cooking oil (10.0 g) was added dropwise to the sulfur over a period of 5 minutes. After 22 minutes of additional reaction time, the mixture reached its gel point and formed a brown rubber. The polymer was removed from the flask with a metal spatula. The product was washed by submerging the polymer chunks in 0.1 M NaOH for 90 minutes, followed by washing with water. Air drying provided the final product.

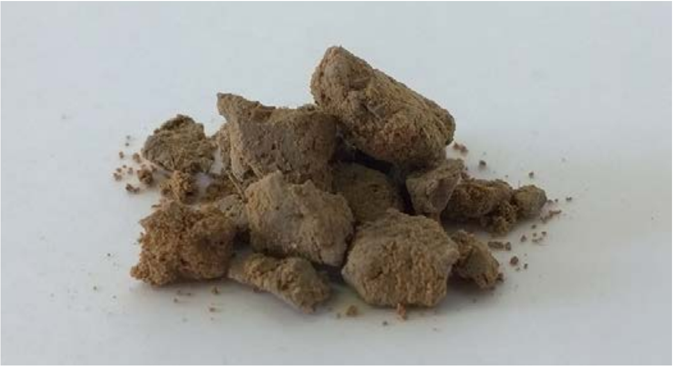

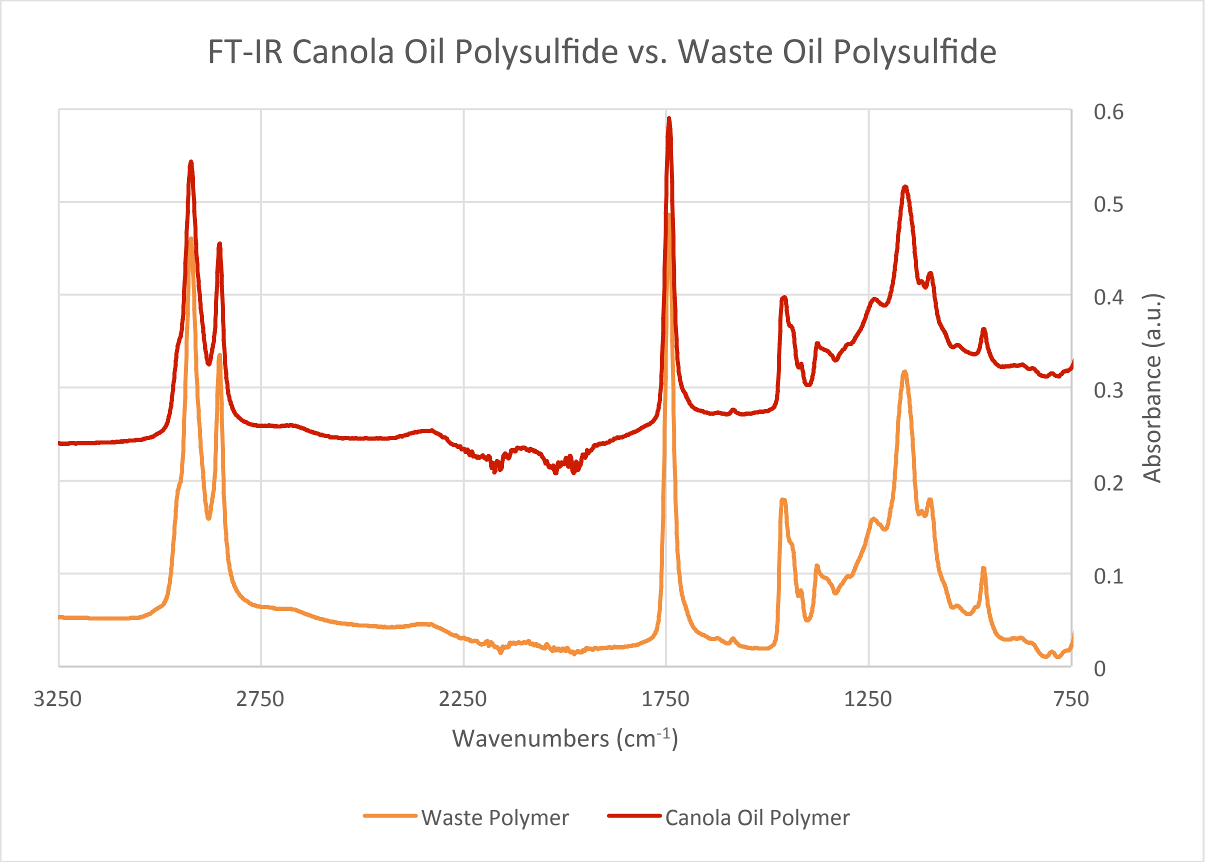

Fig. S7 | Photograph of a polysulfide prepared from used cooking oil (top). IR spectra of the polysulfide prepared from unused canola oil and the polysulfide prepared from waste cooking oil (both 50% sulfur).

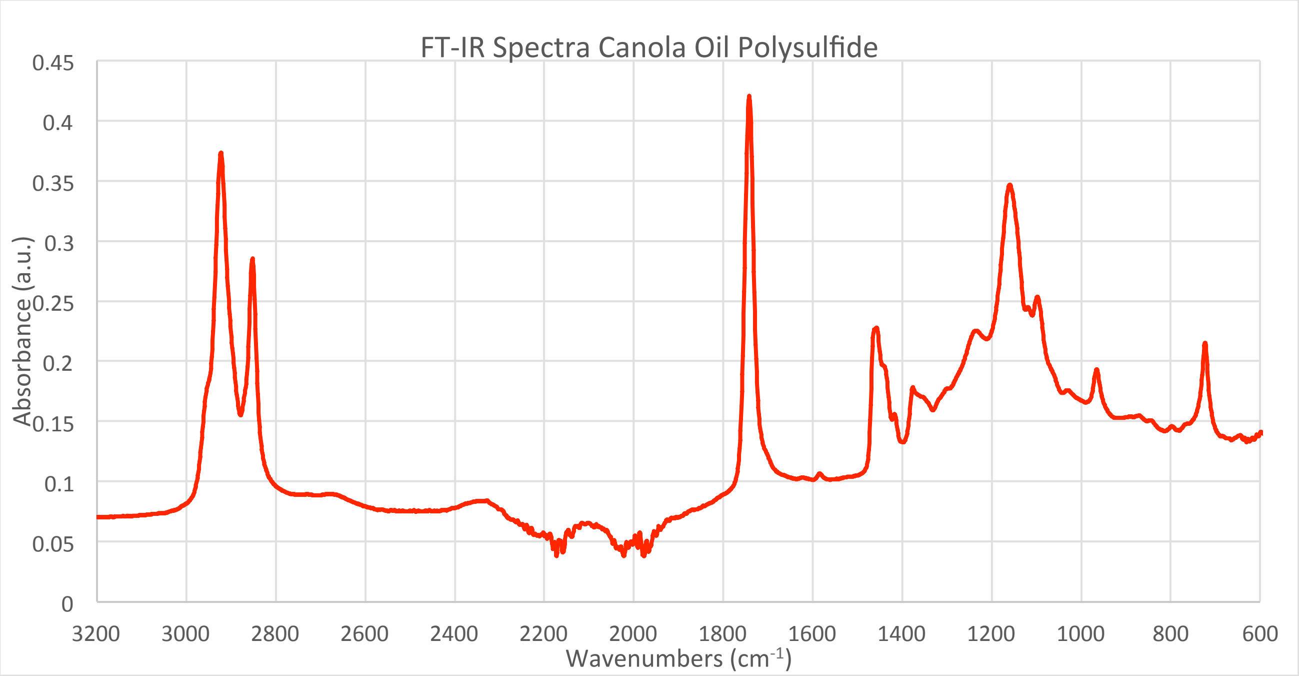

FT-IR spectra overlay of Canola Oil Polysulfide (red) and Canola Oil (blue)

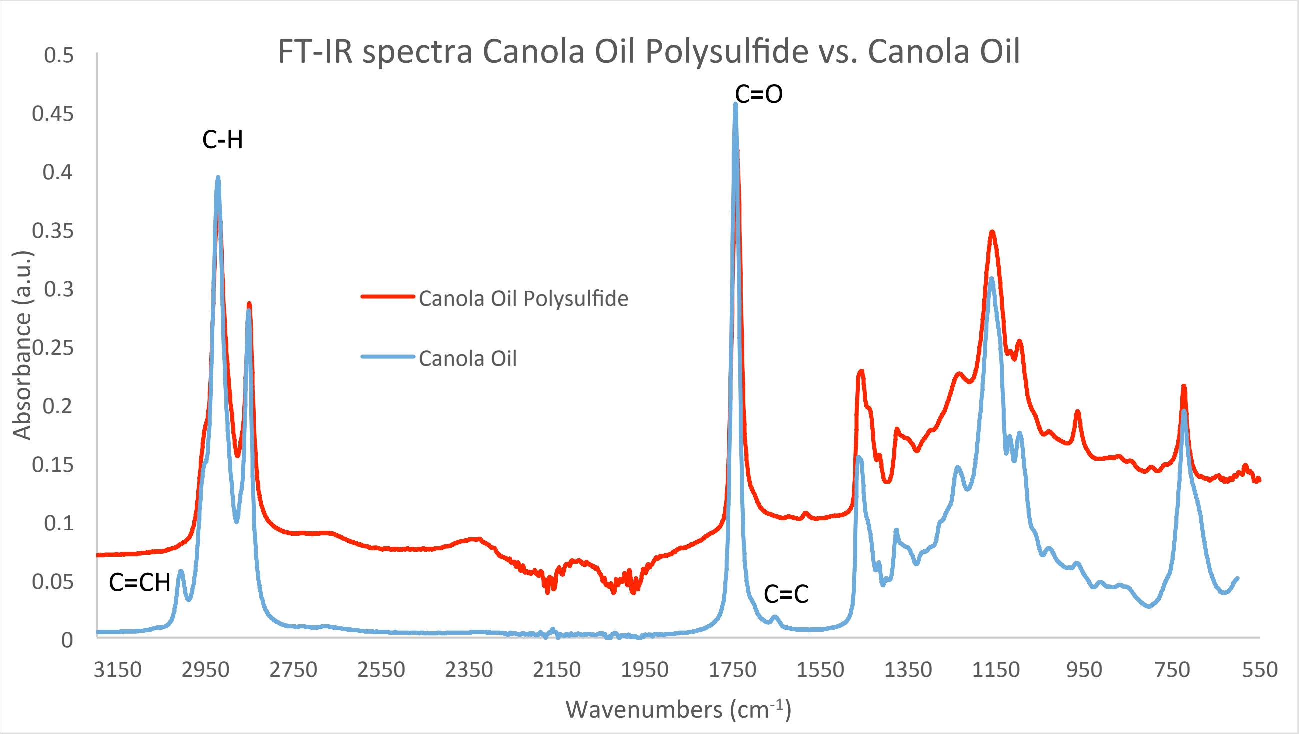

Fig. S8 | FT-IR spectra of Canola Oil Polysulfide (ATR): Key signals include the C=O stretch from the canola oil. The absence of alkene C-H and C=C stretches are consistent with the reaction of sulfur with the alkene.

NMR analysis of $CDCl_3$ soluble fraction of non-porous canola oil polysulfide (50% sulfur).

$CDCl_3$ (10 mL) was added to a sample of non-porous canola oil polysulfide (500 mg). The mixture was stirred vigorously at room temperature for 15 minutes to extract the soluble fraction of the polymer. The resulting solution was filtered and then analysed by ^1H NMR. The spectra are shown below with unreacted canola oil as a reference. The key findings from this experiment are that the soluble fraction of the polymer contains unreacted alkene peaks. However, even in this soluble fraction the ratio of the terminal methyl groups of the fatty acid ester (0.87 ppm) and the alkene peaks (5.25-5.37 ppm) have changed. In the canola oil this ratio is 1.0 : 1.0. and in the soluble fractions of the polymer it is ~3:1, indicated alkene reaction. After NMR analysis, the solvent was evaporated to determine the amount of polymer dissolved. For the non-porous canola oil polysulfide, 61 mg dissolved (12% of the polymer mass). From this result, it can be concluded that in the polymerisation of canola oil and sulfur at a mass ratio of 1:1, the gel point is reached before all alkenes are consumed.

^1H NMR of canola oil (before polymerisation with sulfur)

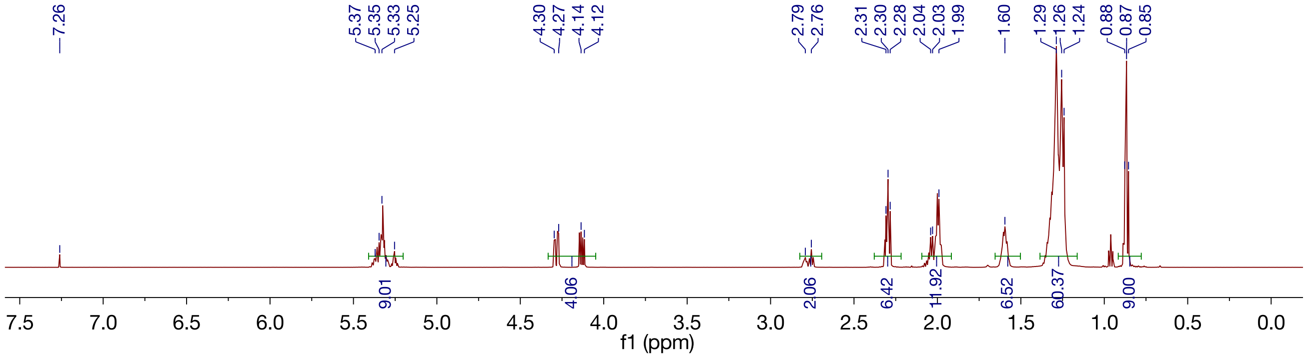

^1H NMR of $CDCl_3$ soluble fraction of non-porous canola oil polysulfide

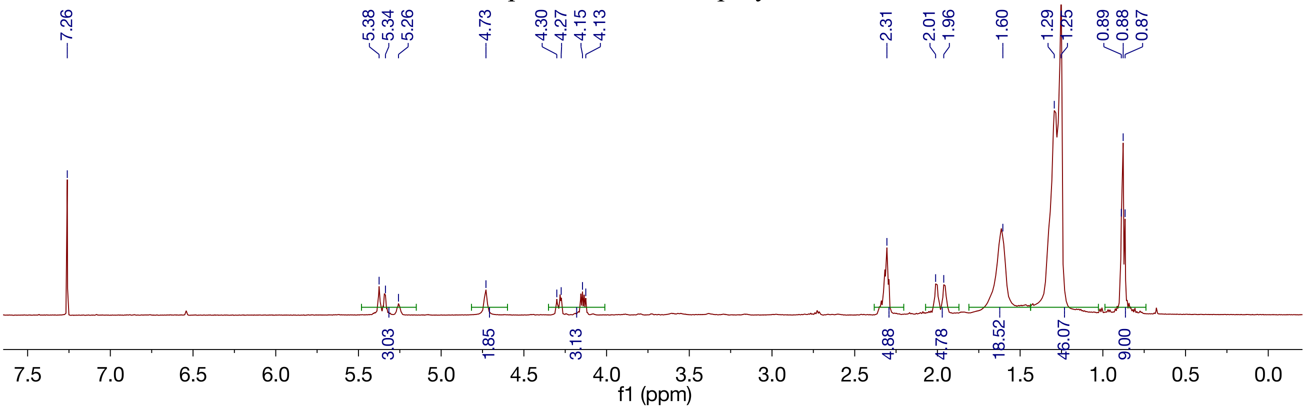

Fig. S9 | ^1H NMR of canola oil, and the soluble fractions of the non-porous canola oil polysulfide

Reaction of sulfur and fatty acid methyl esters derived directly from vegetable oils

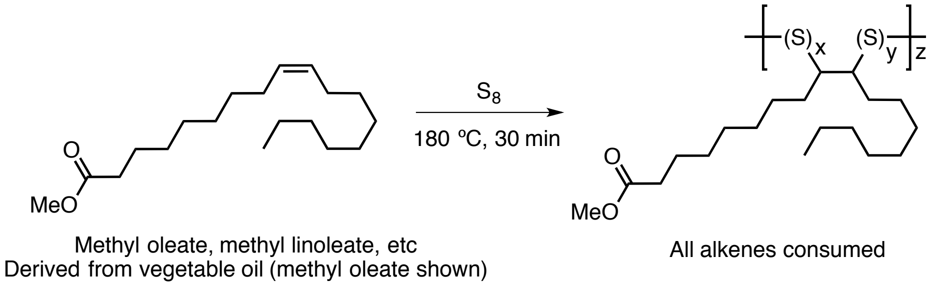

Sulfur and fatty acid methyl ester obtained from canola oil. Sulfur (87 mg, 0.34 mmol S_8) was added to a 100 mL round bottom flask and then heated to 180 ºC with stirring. The methyl ester prepared from transesterification of canola oil with sodium methoxide (Fig S3) (100 mg) was then added to the sulfur. The reaction was stirred at 180 ºC for 30 minutes and then cooled to room temperature to provide a viscous black oil. The mixture was analysed directly by ^{1}H NMR. *All alkene peaks (5.0-5.5 ppm) were consumed in the reaction*:

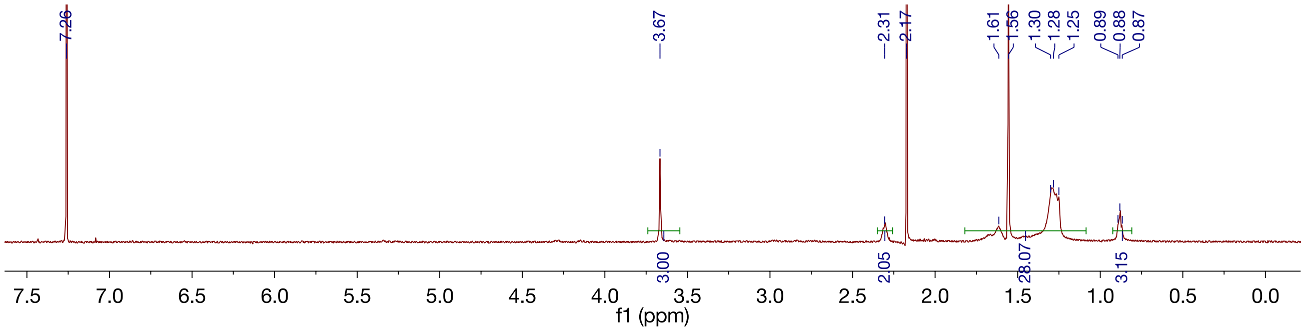

Sulfur and fatty acid methyl ester obtained from sunflower oil. Sulfur (404 mg, 1.56 mmol S_8) was added to a 100 mL round bottom flask and then heated to 180 ºC with stirring. The methyl ester prepared from transesterification of sunflower oil with sodium methoxide (Fig S3) (500 mg) was then added to the sulfur. The reaction was stirred at 180 ºC for 30 minutes and then cooled to room temperature to provide a viscous black oil. The mixture was analysed directly by ^{1}H NMR. All alkene peaks (5.0-5.5 ppm) were consumed in the reaction:

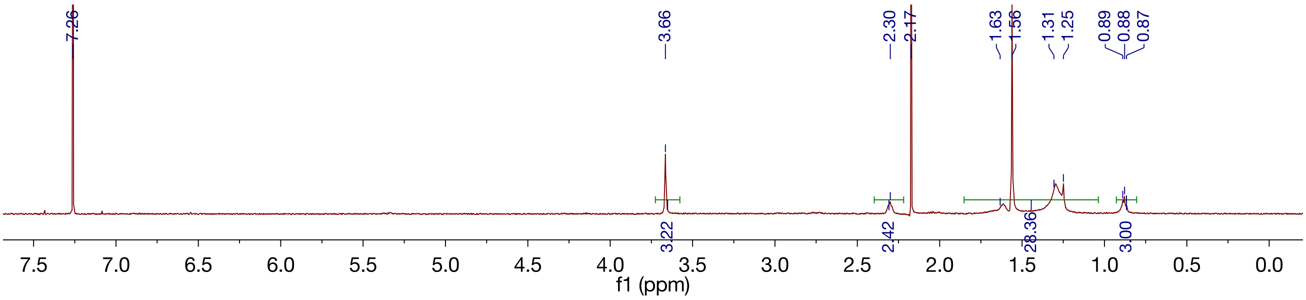

Sulfur and fatty acid methyl ester obtained from olive oil. Sulfur (440 mg, 1.72 mmol S_8) was added to a 100 mL round bottom flask and then heated to 180 ºC with stirring. The methyl ester prepared from transesterification of olive oil with sodium methoxide (Fig S3) (500 mg) was then added to the sulfur. The reaction was stirred at 180 ºC for 30 minutes and then cooled to room temperature to provide a viscous black oil. The mixture was analysed directly by ^{1}H NMR. All alkene peaks (5.0-5.5 ppm) were consumed in the reaction:

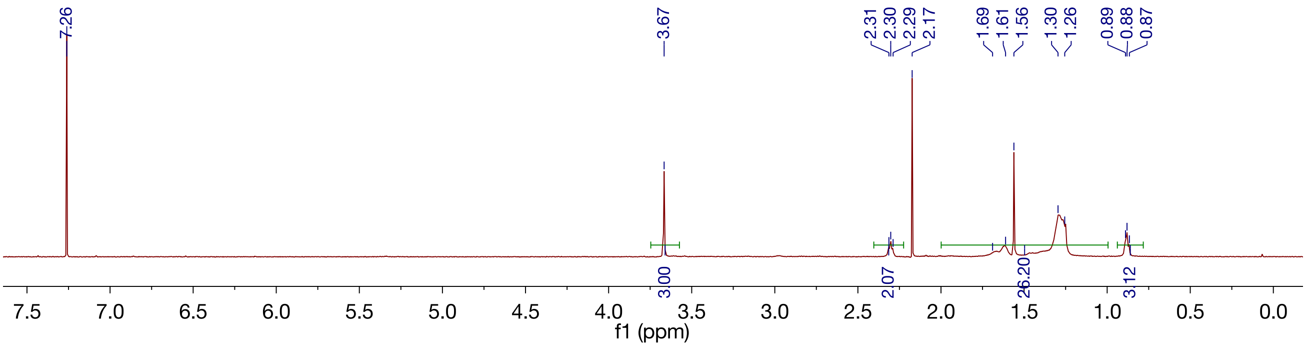

Fig. S10 | Reaction of sulfur and fatty acid methyl esters derived from plant oils. To confirm that sulfur reacts at the alkenes present in the vegetable oils, the reaction between elemental sulfur and the methyl ester derived from each oil was studied.

SEM analysis of Canola Oil Polysulfide (50% sulfur)

The Canola Oil Polysulfide was prepared according to the standard procedure, providing a distribution of particles from 0.2 to 12 mm. These particles were then passed through two polyethylene sieves to obtain particles in the range of 0.5 to 1 mm. A sample of these particles were then mounted on an aluminium SEM pin mount using carbon tape before sputter coating with platinum. Subsequent SEM analysis revealed the surface of the polysulfide to be microtextured—a property that increases surface area.

Canola Oil Polysulfide (before exposure to mercury):

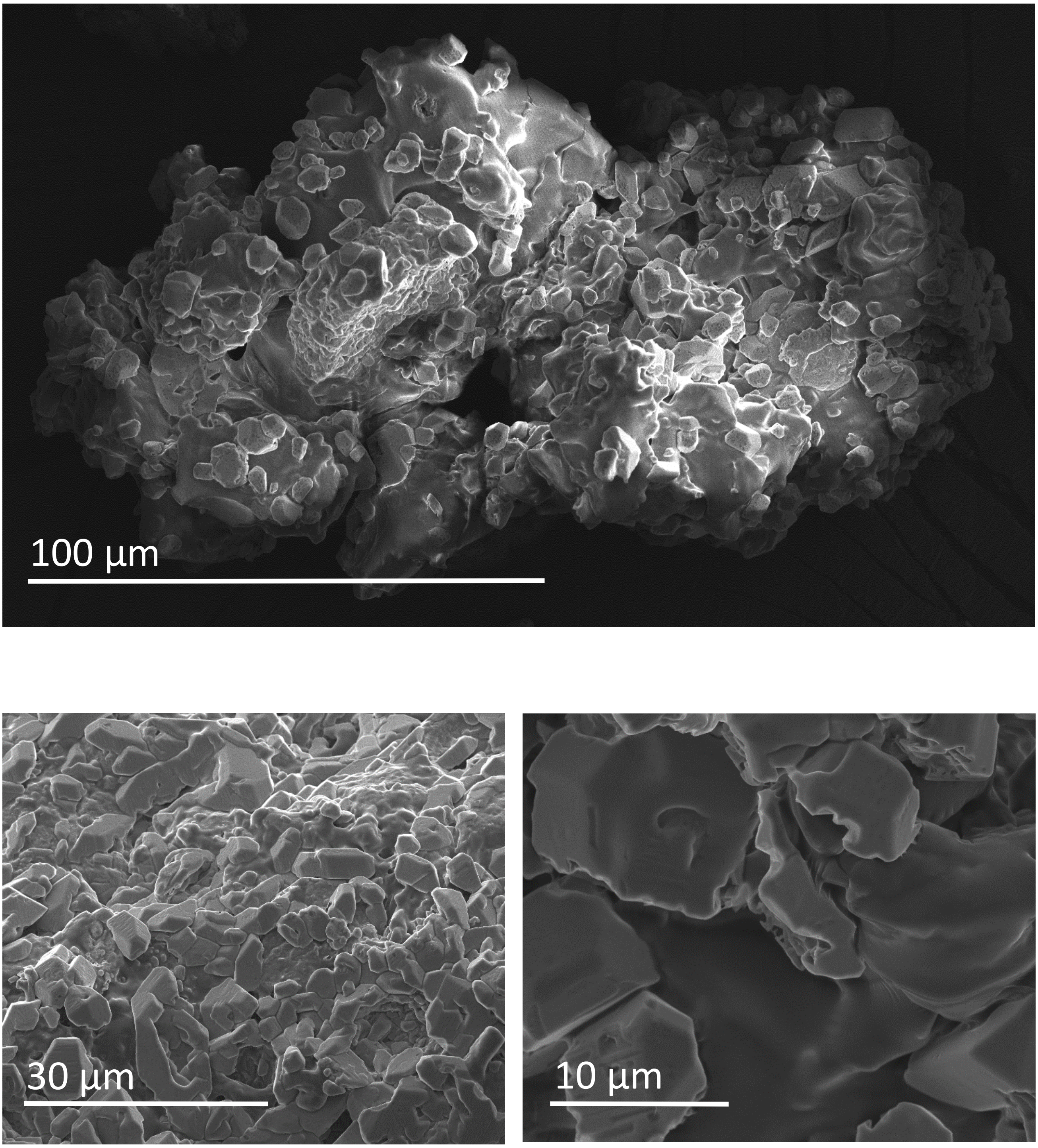

Fig. S11 | SEM analysis of Canola Oil Polysulfide (50% sulfur), shown at three different magnifications

EDS analysis of canola oil polysulfide (50% sulfur)

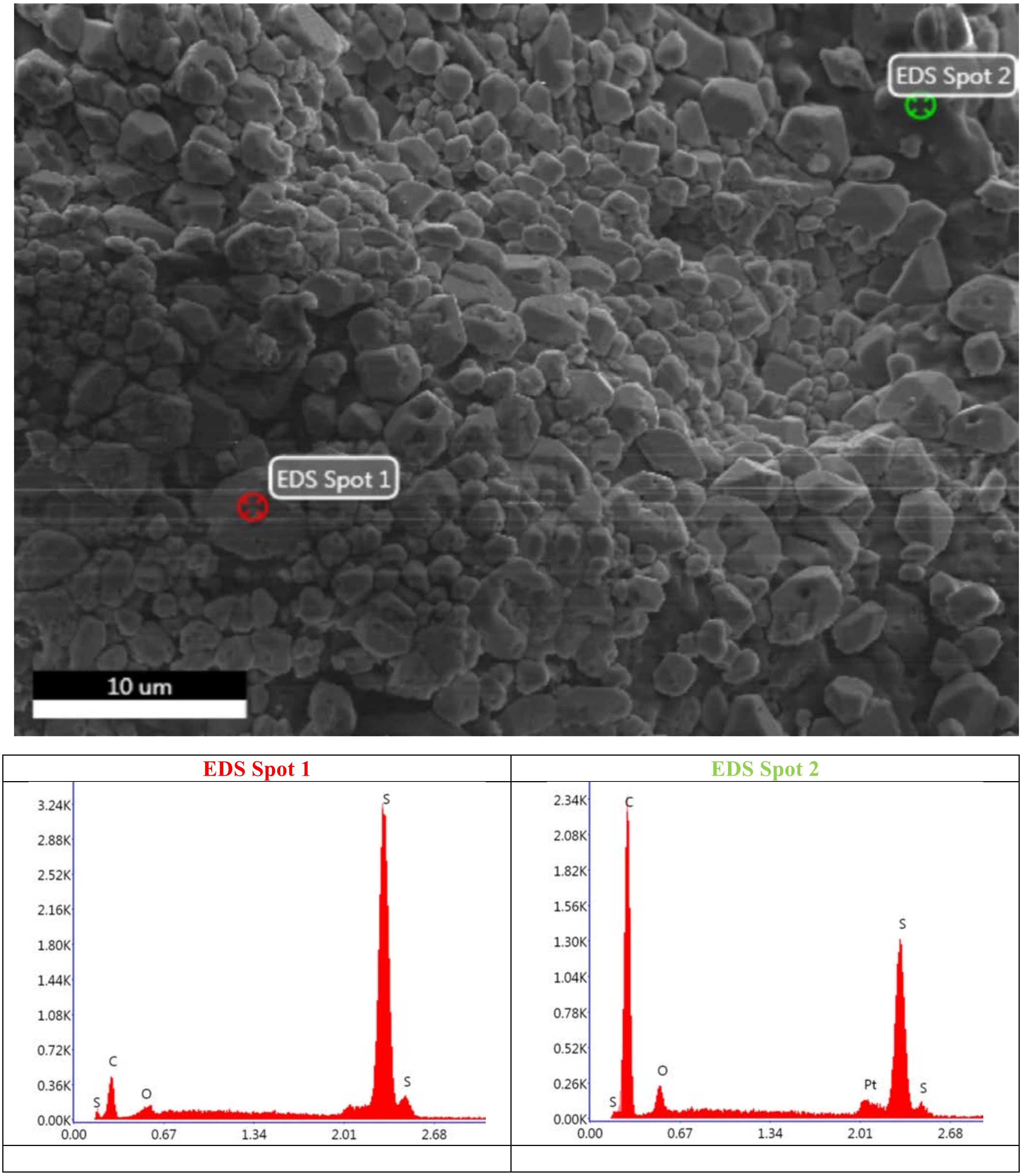

Fig. S12 | EDS analysis of the Canola Oil Polysulfide (50% sulfur) reveals regions of high sulfur, consistent with the polysulfide structure. Note that the sulfur content varies with each region, where some regions are very rich in sulfur (e.g. spot 1) and other regions have more carbon content. The sulfur rich regions are made up of predominately polysulfide (-S-[S]$_n$-S-) polymers and also free sulfur particles. Free sulfur makes up about 9% of the material by mass, as revealed by DSC (cf. Fig. S23)

Scanning Auger Electron Spectromicroscopy of canola oil polysulfide (50% sulfur)

The non-conductive nature of the samples meant that for a useful Auger Electron Spectrum to be obtained, a 2 nm layer of Platinum was needed to provide conductivity to the surface of the sample. The elemental maps of carbon and sulfur show that the carbon-sulphur ratio varies spatially.

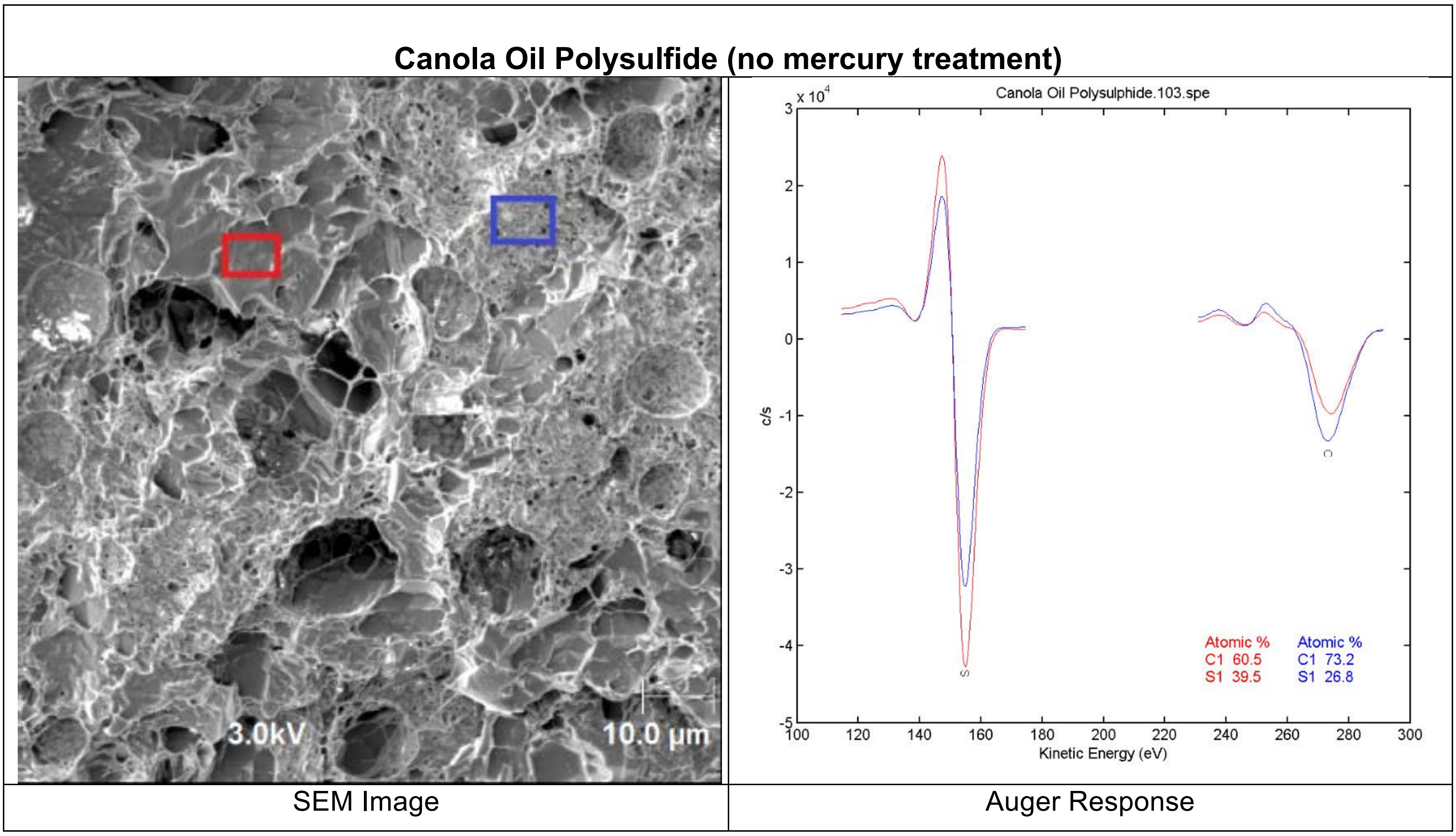

Fig. S13 | Auger spectroscopy of the canola oil polysulfide (50% sulfur) revealed strong signals for carbon and sulfur, consistent with the proposed structure.

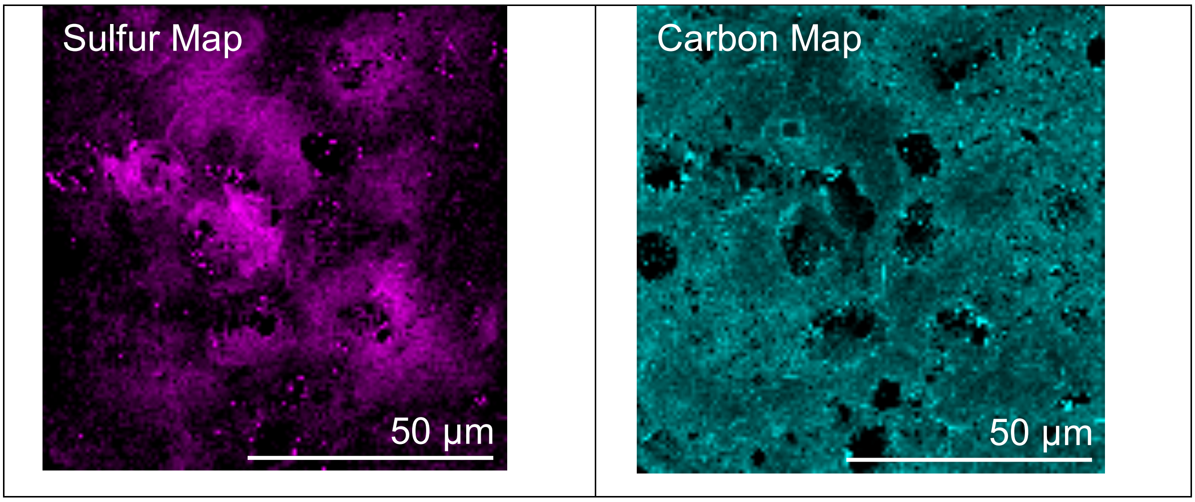

Fig. S14 | Auger imaging of representative sections of the canola oil polysulfide (50% sulfur), with atomic mapping of sulfur and carbon.

Raman Spectra of Canola Oil Polysulfide (50% sulfur)

Raman analysis shows stretches at 432 cm^{-1} and 470 cm^{-1}, consistent with S-S vibrational modes of a polysulfide material. Peaks at 1437 cm^{-1} and 2900 cm^{-1} are attributed to the canola oil domain of the polymer. The Raman spectra for the canola oil polysulfide (50% sulfur), and the canola oil and sulfur starting materials are shown below:

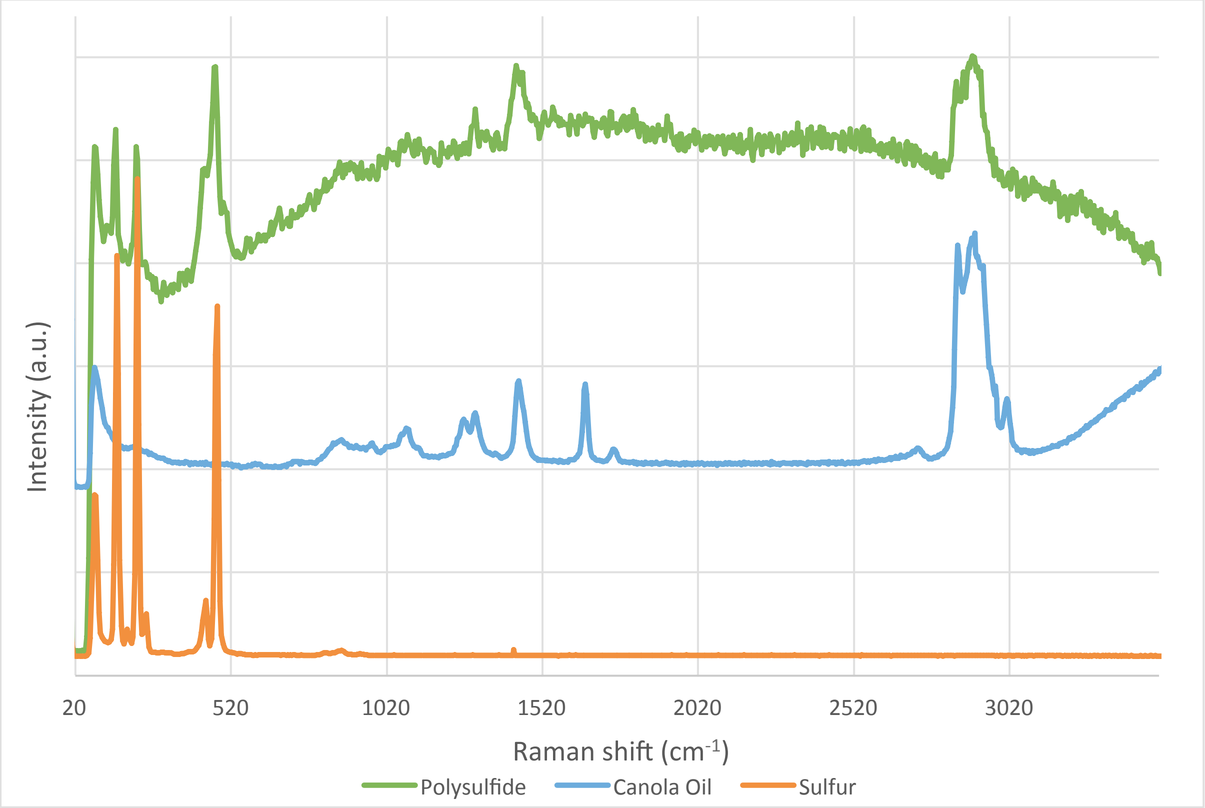

Fig. S15 | Raman spectra of the canola oil polysulfide (50% sulfur) and the canola oil and sulfur starting materials.

Confocal Raman images of Canola Oil Polysulfide (50% sulfur)

Confocal Raman images were acquired for the Canola oil polysulfide and are displayed in Figure S16. Figure S16a is an optical image of the sample with figures S16b and c representing confocal Raman images (30x30 μm) of exactly the same area of the sample. Figures S16d and e are zoomed in Raman images (15x15 μm) of the same area with the centre of each image corresponding to the white and black crosses in figures S16 b and c. The data in figures S16b and d were generated by plotting the intensity of the 470 cm^{-1} region of each Raman spectrum while the data in figures S16c and e were generated by plotting the intensity of the 2900 cm^{-1} region of each Raman spectrum. The Raman spectra that are present in the brighter regions of figures S16b and d typically have the appearance of the sulfur starting material displayed in figure S15 (orange curve) and the Raman spectra that are present in the brighter regions of figure S16b and e typically have the appearance of the canola oil polysulfide copolymer (50% sulfur) also displayed in figure S15 (green curve). It is apparent from figure S16b that there are regions of free sulphur embedded in the polysulfide matrix that form what appear to be small microparticles (5 to 15 μm in size). This data supports the SEM/EDS analysis as well as other results recently reported in the literature on related composites.[1]

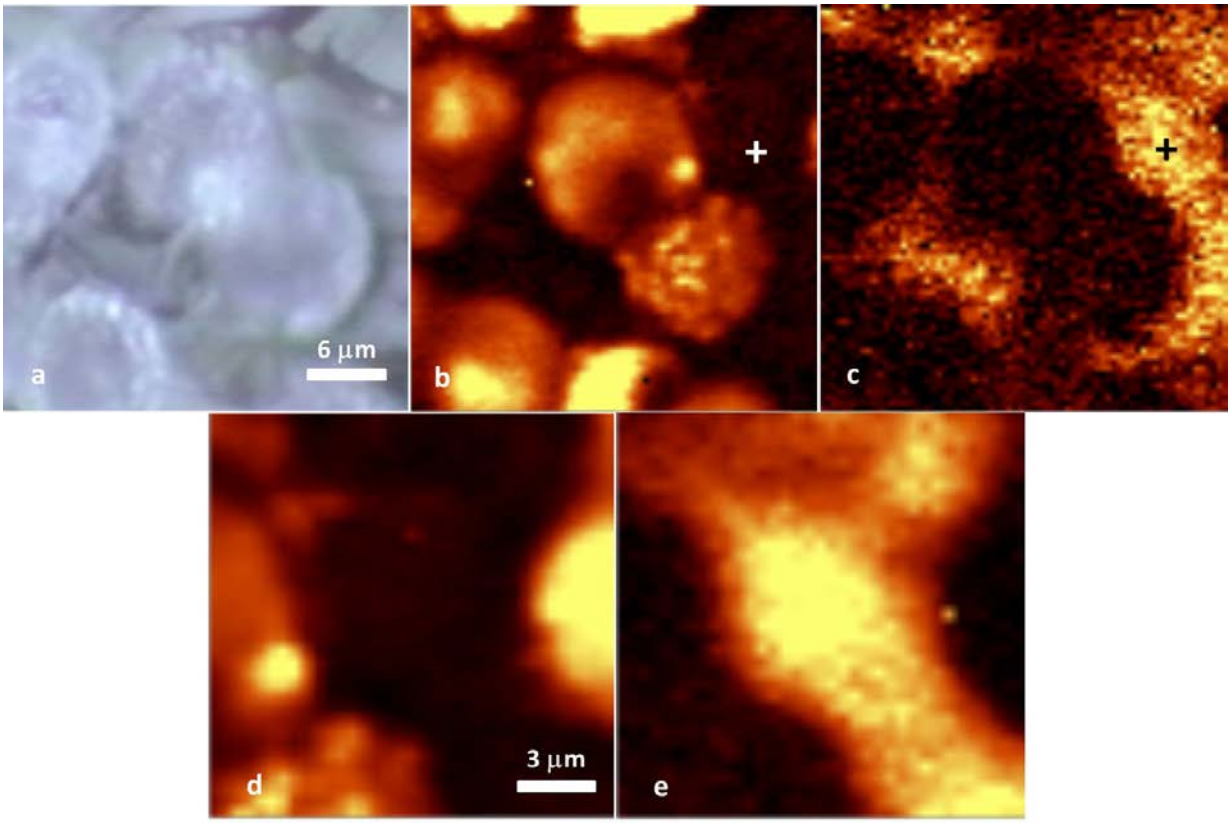

Fig. S16 | Optical image (a) of a section of the canola polysulfide with corresponding confocal Raman images of the same region (b and c). The number of pixels in b and c is 70x70 (4900) with the integration time per pixel equal to 1 second. The confocal Raman images in d and e are zoomed in areas of b and c and correspond to exactly the same area of the sample. The centre of each image in d and e is denoted by the white and black crosses displayed in b and c. The number of pixels in d and e is 35x35 (1225) with the integration time per pixel equal to 6 seconds.

Analysis of thiol-content on the canola oil polysulfide surface using Ellman's test

A sample of canola oil polysulfide (1.00 g, 50% sulfur) was placed into each of three 50 mL centrifuge tubes along with 8 mL phosphate buffer (100 mM, pH 8) and Ellman's reagent (8 mg, 0.020 mmol). As a control, Ellman's reagent was also added to three separate samples of buffer in the same way, except in the absence of polymer. All samples were mixed on a lab rotisserie for 1 hour at room temperature before filtering. The filtrates were then diluted 7-fold and analysed by UV-Vis spectroscopy. Absorbance at 412 nm are listed below. No reaction with Ellman's reagent was observed, as no significant increase in absorbance at 412 nm was observed (student t-test). Therefore, thiol content on the polymer is negligible and consistent with the proposed polysulfide structure.

	Absorbance of sample prepared using Ellman's reagent and no polymer (negative control showing absorbance of Ellman's reagent alone)	Absorbance of sample prepared using Ellman's reagent and polymer
Sample 1	0.0720	0.0753
Sample 2	0.0637	0.0762
Sample 3	0.0822	0.0628

Fig. S17 | Ellman's test for thiol content on the canola oil polysulfide (50% sulfur). No thiols were detected.

Thermogravimetric Analysis of the Canola Oil Polysulfide at Different Sulfur Compositions

TGA of the canola oil polysulfide was carried out for the canola oil polysulfide prepared at 30, 50, 60 and 70% sulfur by weight. The first major mass loss at ~250 ºC increased in proportion to the amount of sulfur in the polymer. We therefore attribute the first mass loss to thermal degradation of the polysulfide domain of the polymer. Consistent with this interpretation, the end of the first mass loss of each polymer (400 ºC) corresponds well with the mass of sulfur in each polymer (30% mass loss for the 30% sulfur polysulfide , 50% mass loss for the 50% sulfur polysulfide, 60% mass loss for the 60% sulfur polysulfide and 70% mass loss for the 70% sulfur polysulfide). The second mass loss occurs upon decomposition of the canola oil domain of the polymer.

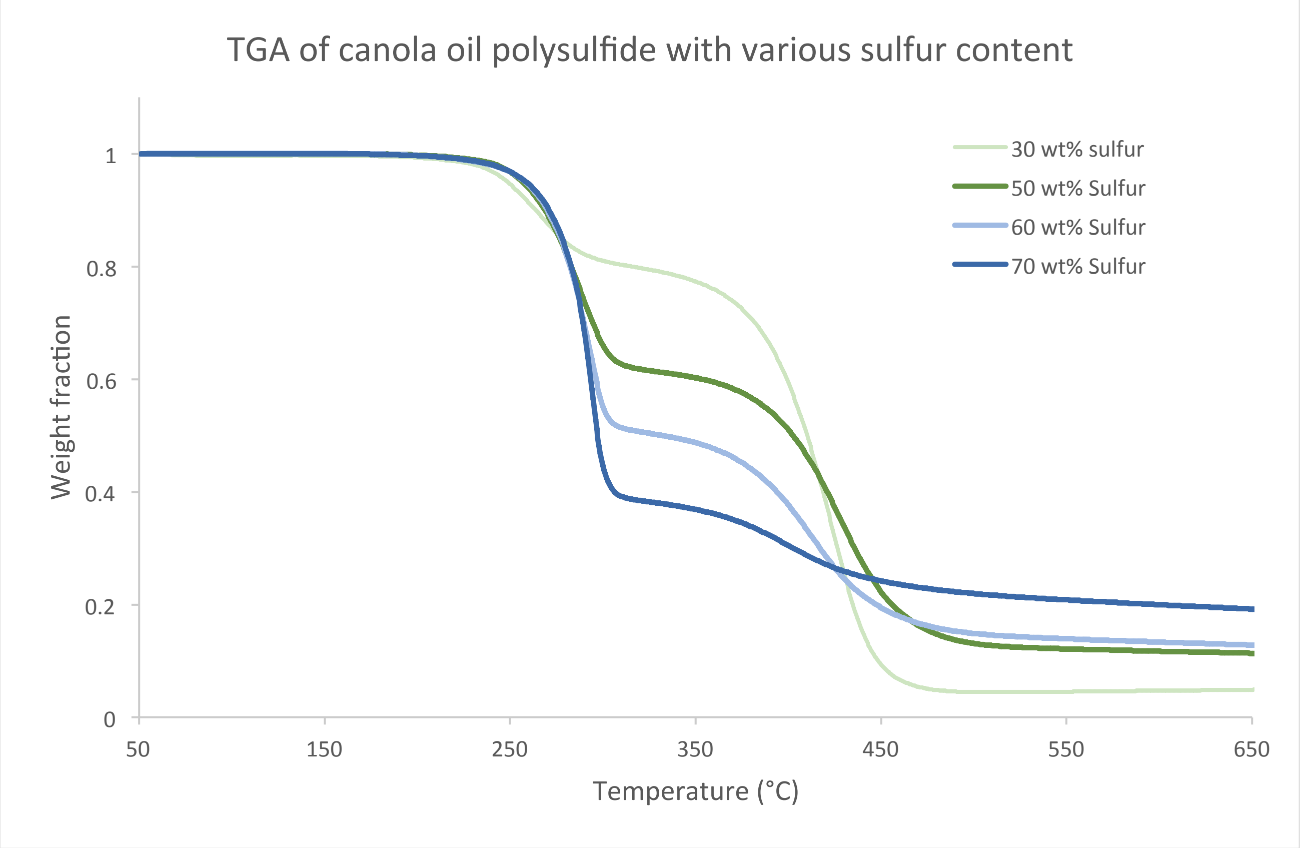

Fig. S18 | Thermogravimetric Analysis of the Canola Oil Polysulfide at Different Sulfur Compositions.

Simultaneous thermal analysis (DSC and TGA) of vegetable oils used in the synthesis of the polysulfides

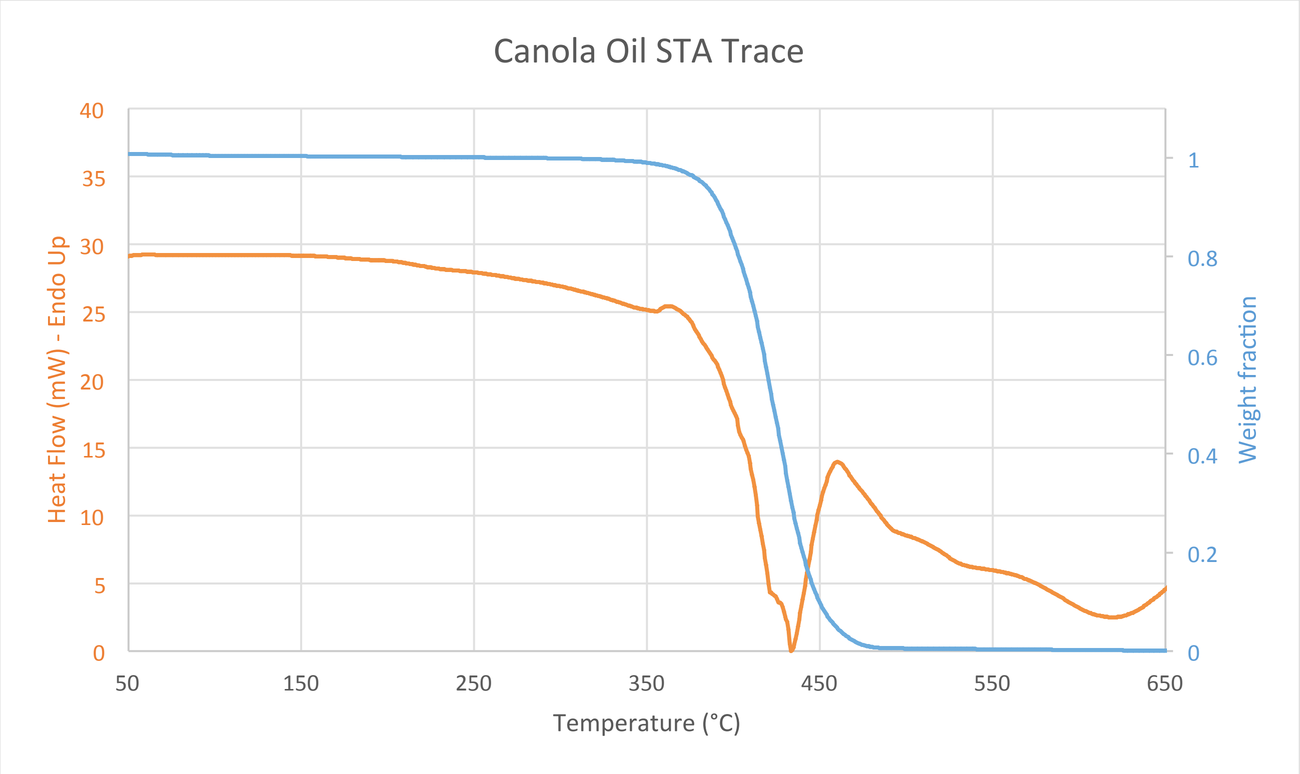

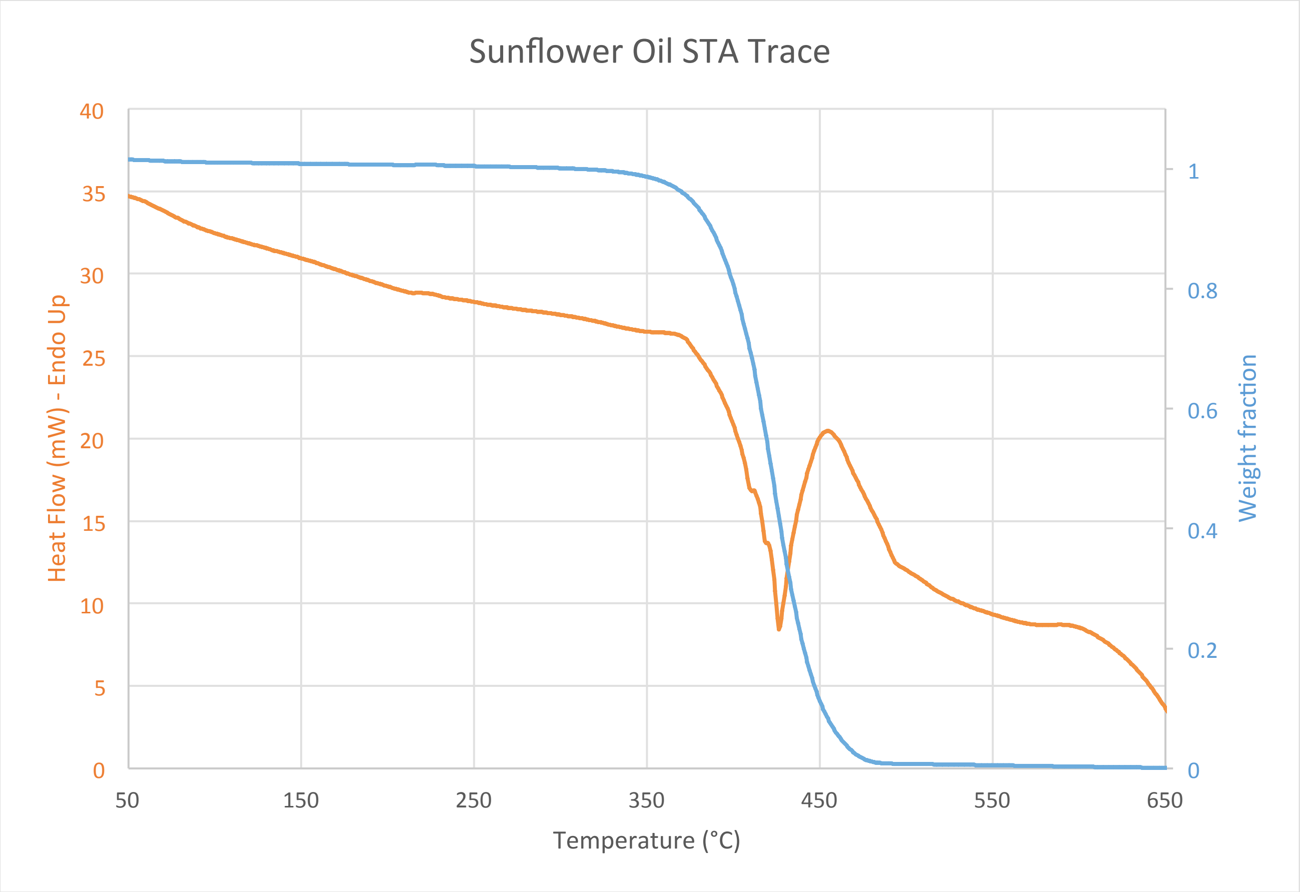

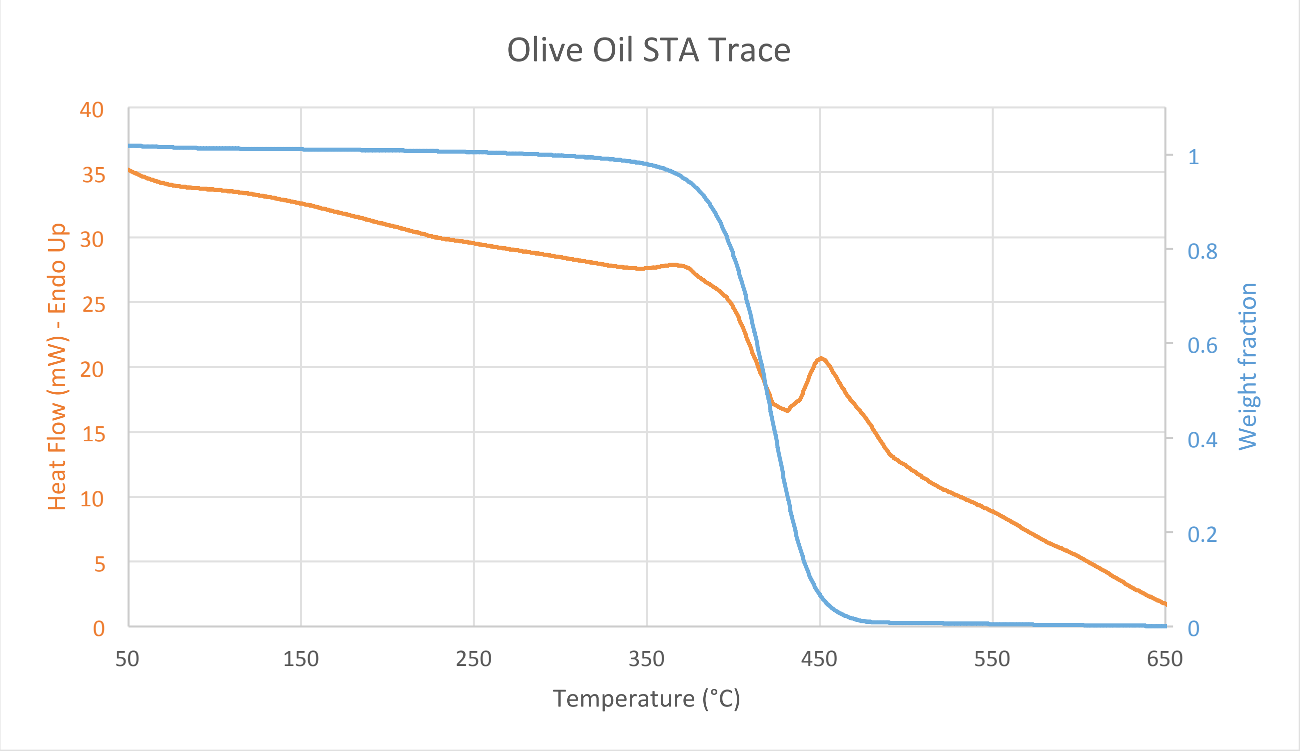

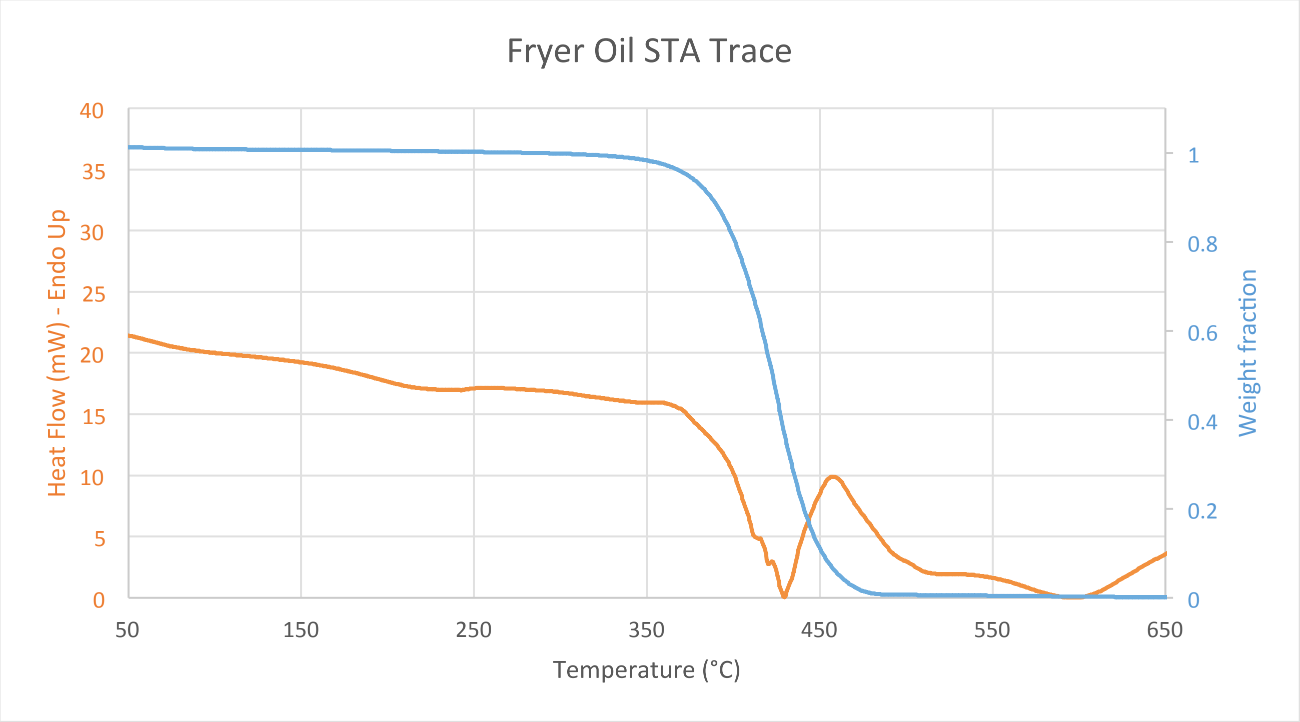

Fig. S19 | DSC (orange) and TGA (blue) traces for the vegetable oils used in the synthesis of all polysulfides.

Simultaneous thermal analysis (DSC and TGA) of sulfur used in the synthesis of the polysulfides

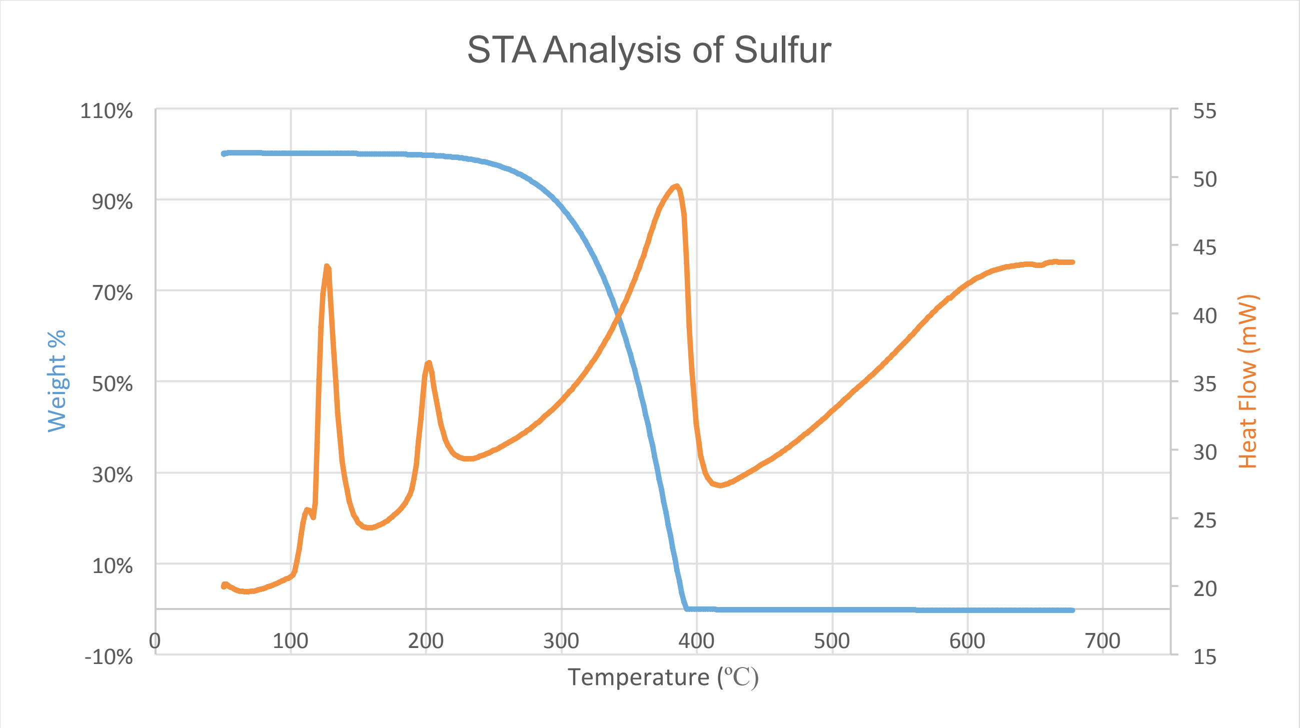

Fig. S20 | DSC (orange) and TGA (blue) traces for the elemental sulfur used in the synthesis of all polysulfides.

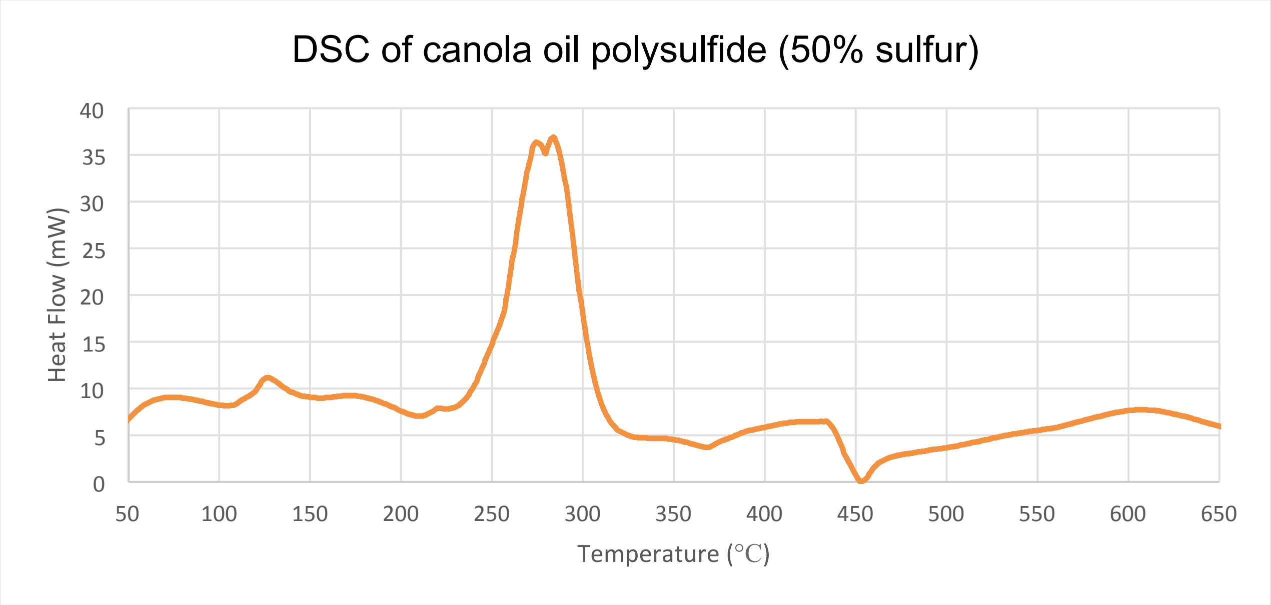

Fig. S21 | DSC trace for the canola oil polysulfide (50% sulfur). The small endotherm between 100 to 150 ºC corresponds to the melting transition of free sulfur. The large endotherm from 230 ºC corresponds to the thermal decomposition of the polysulfide.

DSC of canola oil polysulfide prepared at different sulfur compositions to determine free sulfur content

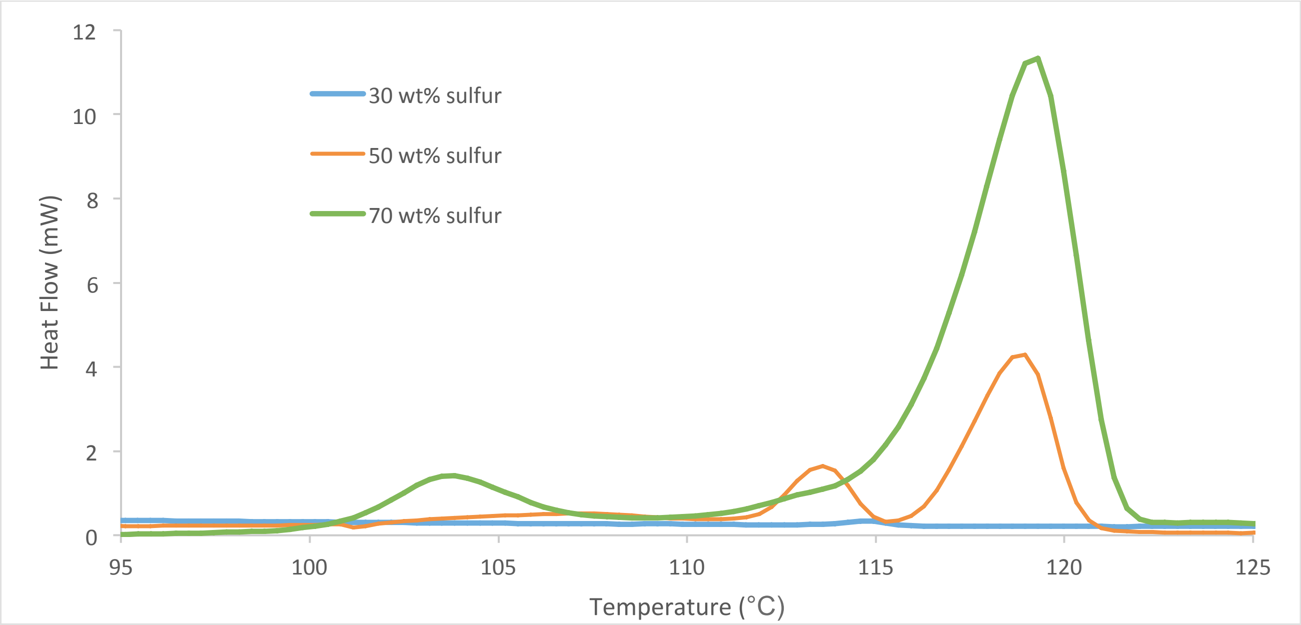

Fig. S22 | DSC of polysulfides prepared from canola oil with various amounts of sulfur. While the DSC curve was largely the same from sample to sample, subtle variations in the region between 100 and 125 ºC were noted, as shown in the plot. These endotherms correspond to the melting of free sulfur.

Estimation of free sulfur in canola oil polysulfides

Quantitative DSC was used to determine free sulfur content in the Canola Oil Polysulfides. S_8 has a distinctive DSC peak at 125 °C that stretches from 100 °C to 150 °C. The area of this peak (from 100 °C to 150 °C) increases linearly with sulfur mass. On average 1 mg sulfur gave a response of 49.3 J/g within the range tested. This response was used to approximate the free sulfur present in the polymer. Because the free sulfur may be present in forms other than S_8, this is only an estimate. The calibration curve is show below:

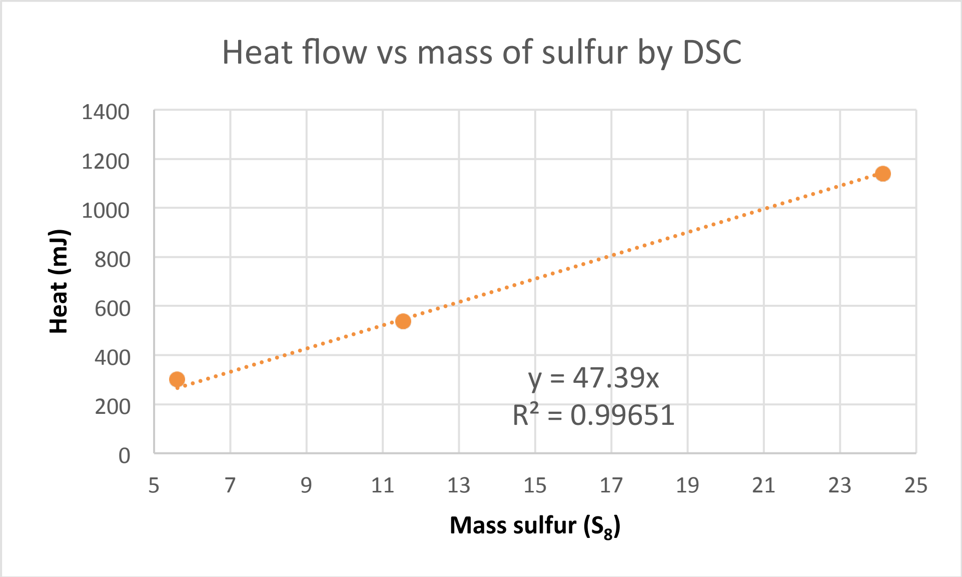

**Sample**	**ΔH (J/g)**	**Free sulfur (% mass)**
Canola Oil Polysulfide (30 wt% Sulfur)	1.866	3.8
Canola Oil Polysulfide (50 wt% Sulfur)	4.408	9.0
Canola Oil Polysulfide (60 wt% Sulfur)	11.467	23.3
Canola Oil Polysulfide (70 wt% Sulfur)	18.721	38.1
Olive Oil Polysulfide (50 wt% Sulfur)	8.429	17.1
Sunflower Oil Polysulfide (50 wt% Sulfur)	7.453	15.2
Waste Oil Polysulfide (50 wt% Sulfur)	7.667	15.6
Classically Vulcanised "Factice" (50 wt% Sulfur)	4.317	8.8

Fig. S23 | Estimation of free sulfur by integration of DSC endotherm from 100 to 150 ºC. Above 30% sulfur by mass, the polysulfides appear to contain significant quantities of free sulfur. For the canola oil polysulfide used in mercury capture experiments (50% sulfur), it is estimated to contain 9% free sulfur by mass.

TGA of polysulfides prepared from canola oil, sunflower oil, and olive oil

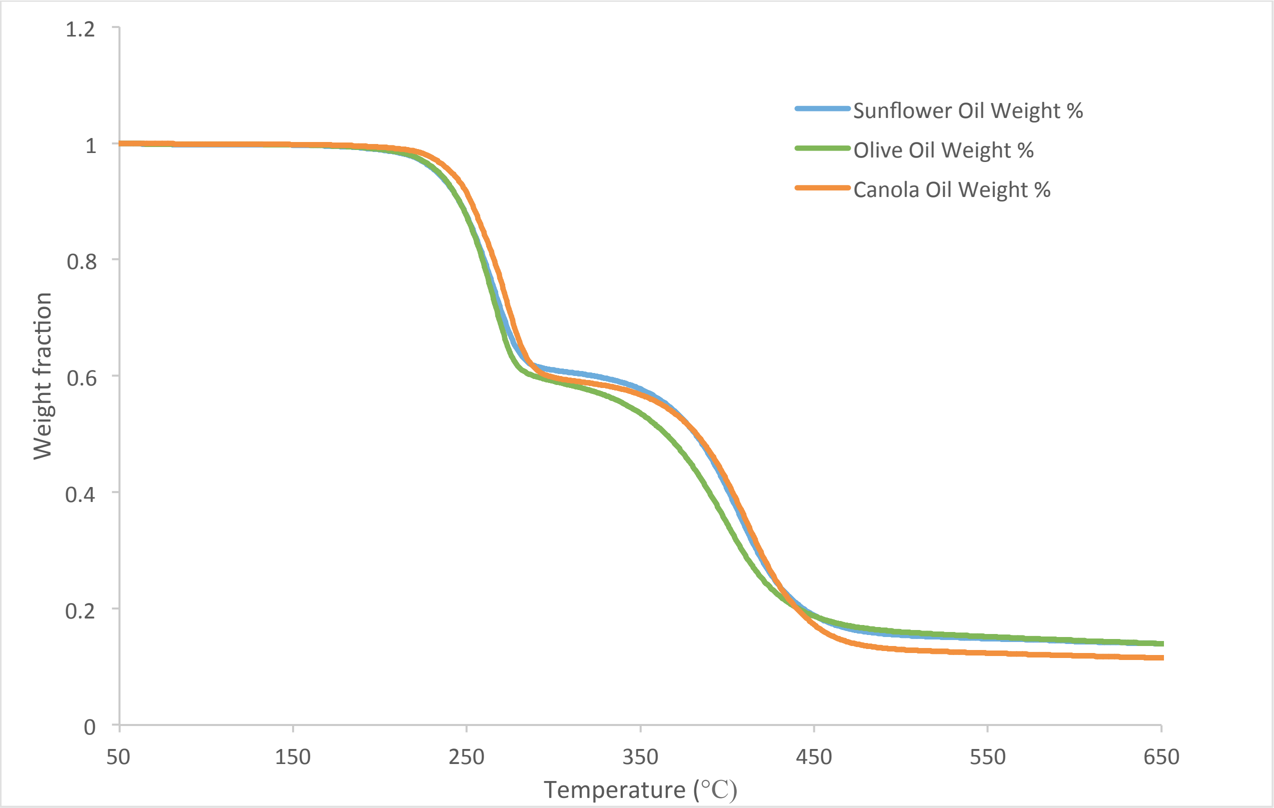

Fig. S24 | TGA of polysulfides prepared by the inverse vulcanisation reaction between sulfur and canola oil, sunflower oil, or olive oil. Similar profiles were observed for all of these polysulfides.

DSC of polysulfides prepared from canola oil, sunflower oil, olive oil and recycled cooking oil

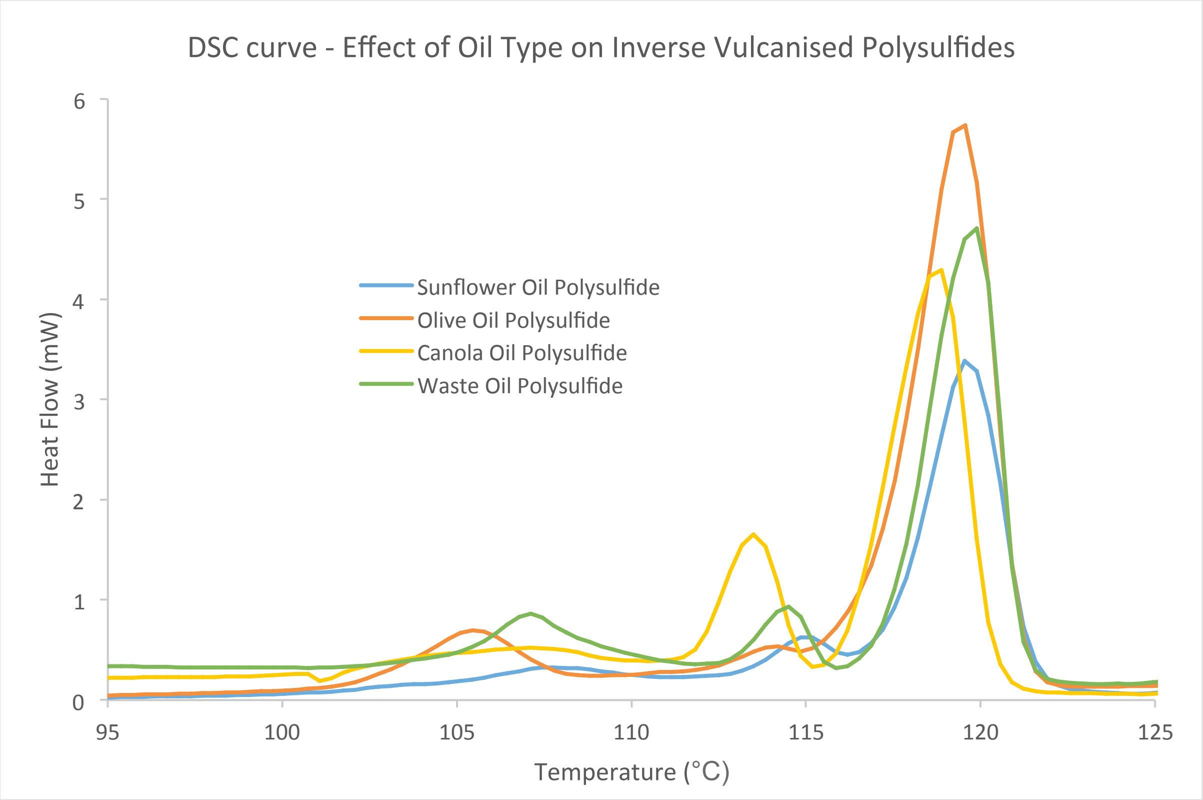

Fig. S25 | Normalised DSC of polysulfides prepared from canola oil, sunflower oil, olive oil and recycled cooking oil in the free sulfur region. While the TGA and DSC were largely the same (see below for full DSC) regardless of the oil source, subtle variations in the region between 100 and 125 ºC were noted, as shown in the figure. These endotherms correspond to the melting of free sulfur. The full DSCs in a repeat measurement of the DSC are shown in the Figure below.

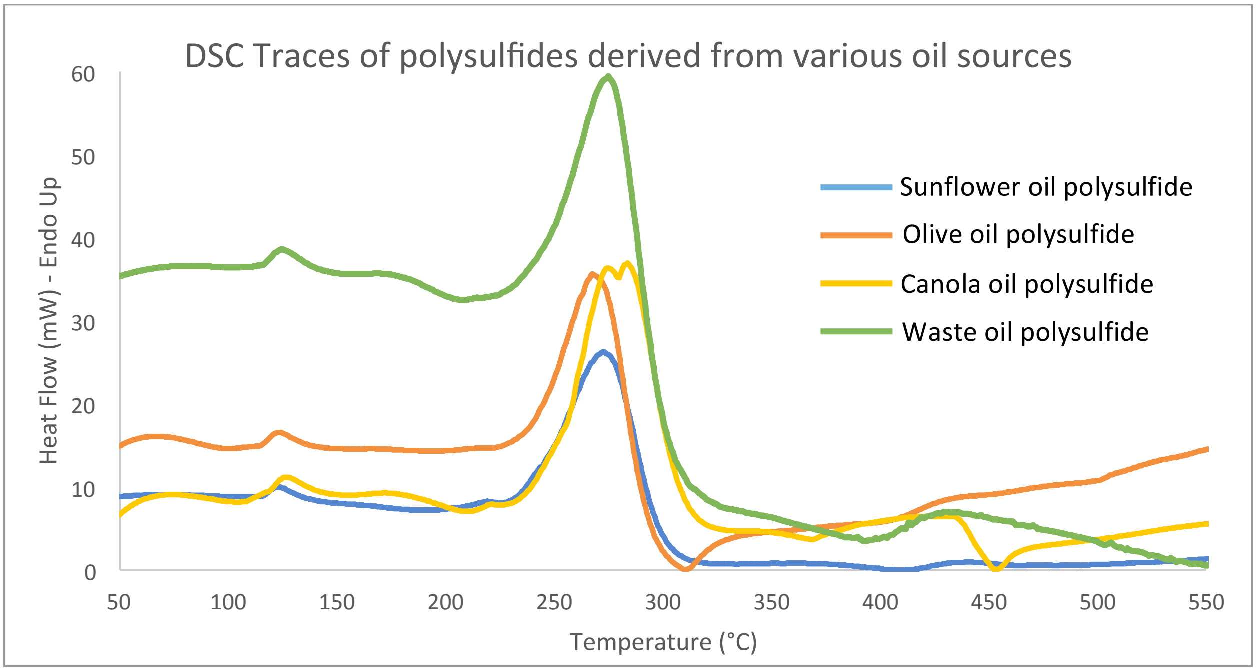

Fig. S26 | DSC of polysulfides prepared from canola oil, sunflower oil, olive oil and recycled cooking oil

Comparison of Canola Oil Polysulfide and Factice

Factice is a commercially available additive used extensively in the rubber industry. Factice is made through classic vulcanisation of vegetable oils, such as canola oil. Typically, this involves adding low percentages of sulfur to hot vegetable oil, resulting in cross-linking of the oil. In contrast, the canola oil polysulfide reported in this manuscript is prepared by *inverse vulcanisation* where the vegetable oil is added to high mass percentages of liquid sulfur, thereby crosslinking the polysulfide. Because both factice and the canola oil polysulfide are made with similar starting materials, we were interested to compare the two materials directly (spectroscopically, thermally and in its binding to mercury). Shown below, along side the canola oil polysulfide, are photographs of factice samples with 10%, 17% and 25% sulfur. These samples were generously provided by D.O.G. Chemie.

Canola Oil Polysulfide (50% sulfur)	Factice (F10) (10% Sulfur)	Factice (F17) (17% Sulfur)	Factice (F25) (25% Sulfur)

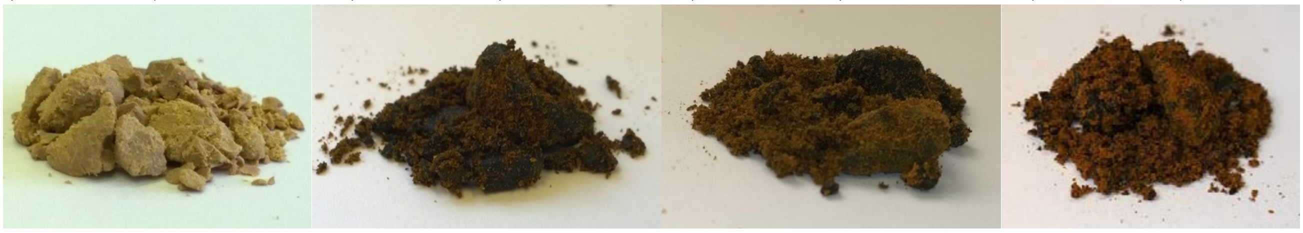

FTIR spectra indicate a very similar absorbance profile for the canola oil polysulfide and factice:

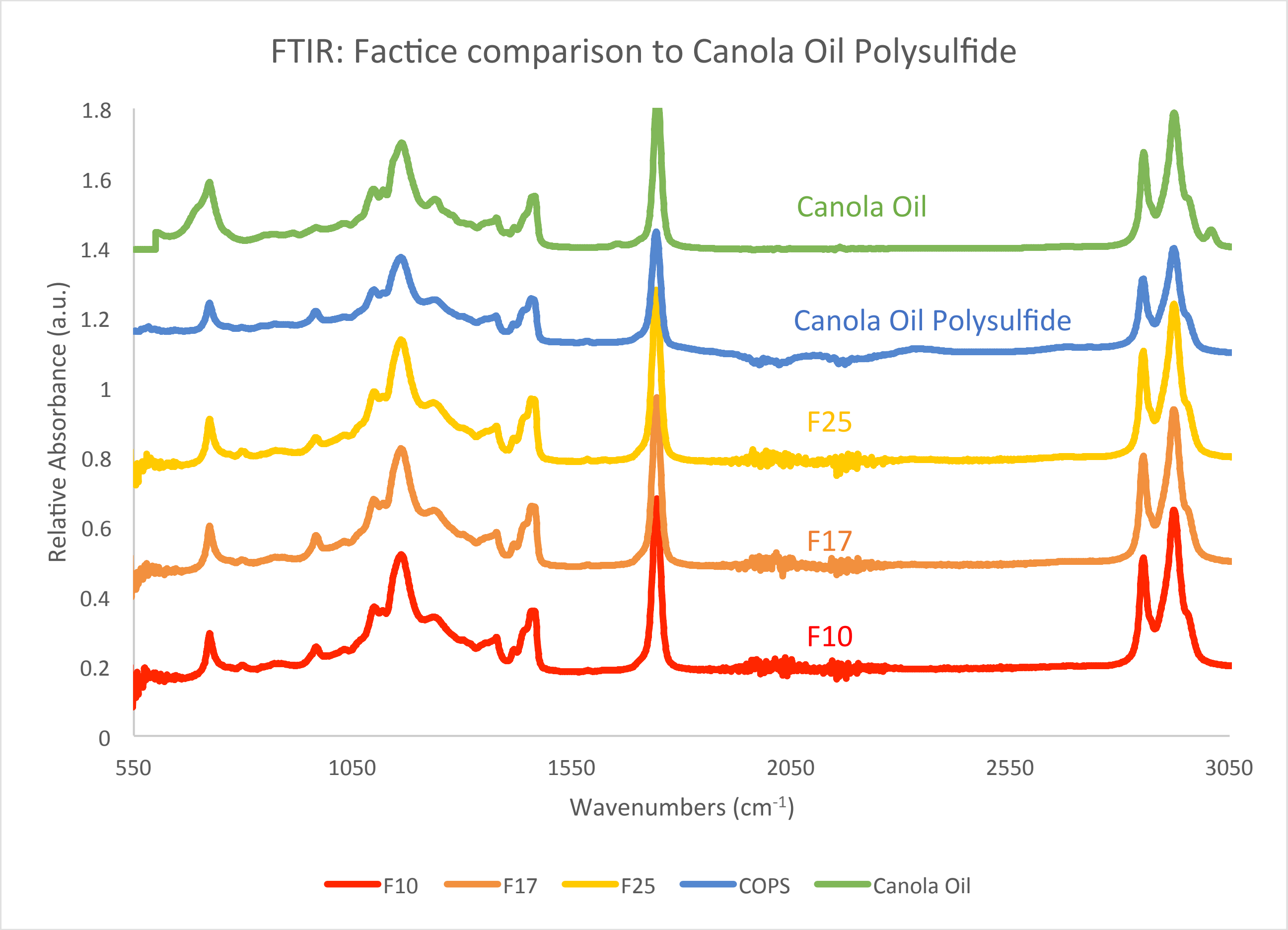

Fig. S27 | IR spectra for factice and the canola oil polysulfide (50% sulfur)

Raman Spectra of factice and canola oil polysulfide:

Raman spectra were obtained for F10 and F25 Factice and compared to the canola oil polysulfide (50 wt% sulfur) prepared by inverse vulcanisation. The increased sulfur content results in an increased intensity of peaks at 432 and 470 cm^{-1}. This is consistent with greater polysulfide (S-$[S]_n$-S) content in the 50 wt% canola oil polysulfide in comparison to F10 or F25 Factice

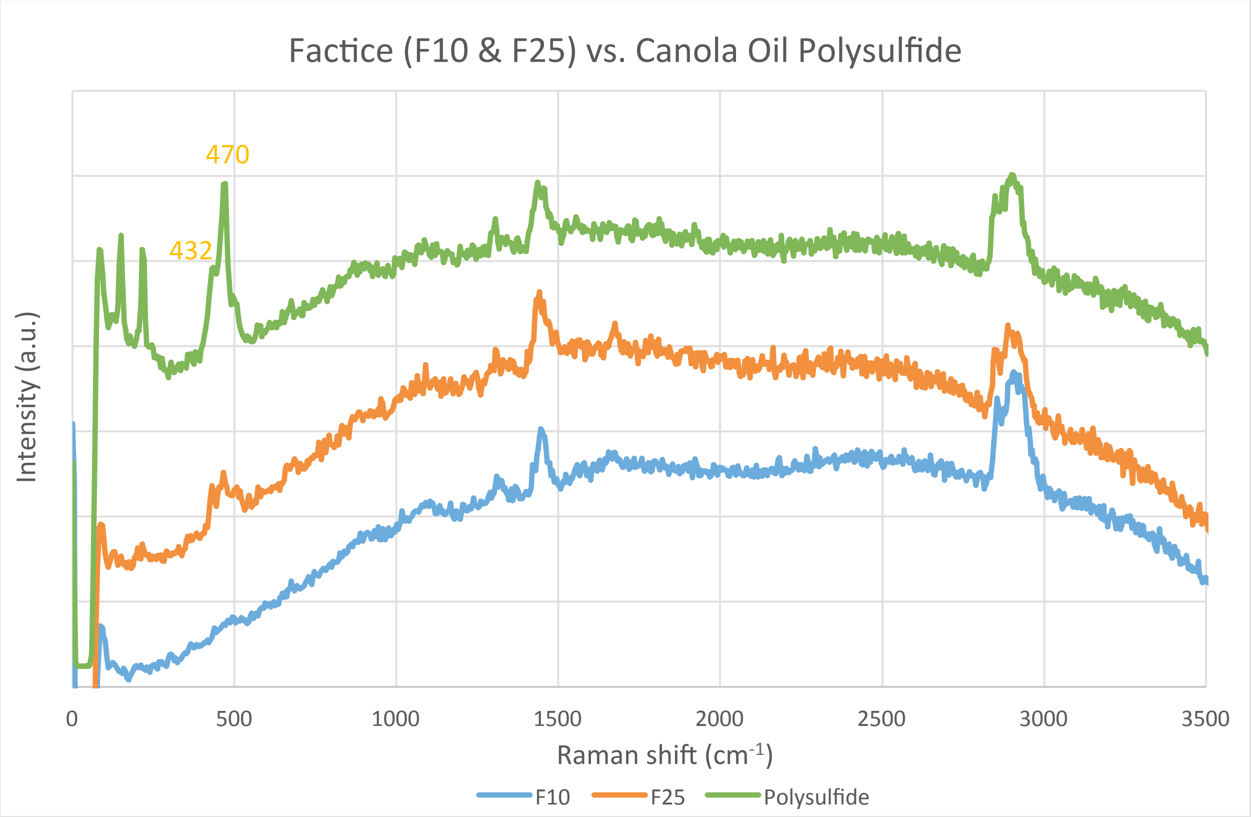

Fig. S28 | Raman spectra for factice and the canola oil polysulfide (50% sulfur)

Simultaneous Thermal Analysis of Factice (F10, F17, F25)

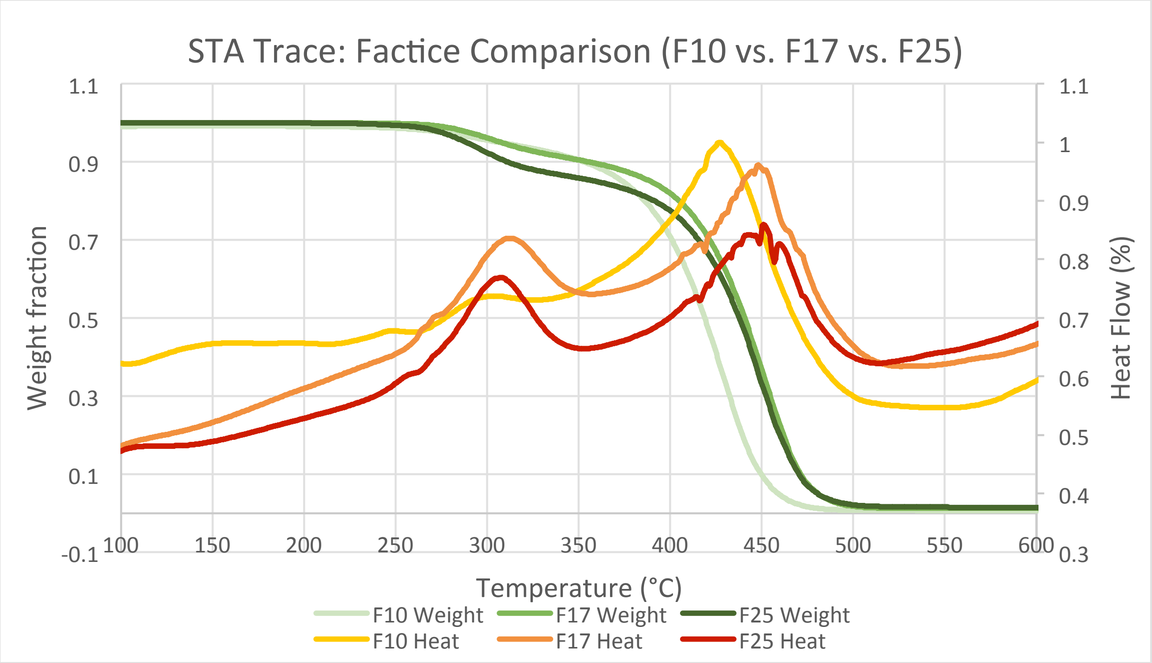

Fig. S29 | Simultaneous thermal analysis (DSC and TGA) for factice. All factice samples reveal similar DSC and TGA profiles with a minor mass loss at 280 ºC and a major mass loss at 400 ºC (Above). In contrast, the canola oil polysulfide (50% sulfur) exhibits a sharp mass loss beginning at < 250 ºC and then a second mass loss > 340 ºC (Fig. S18).

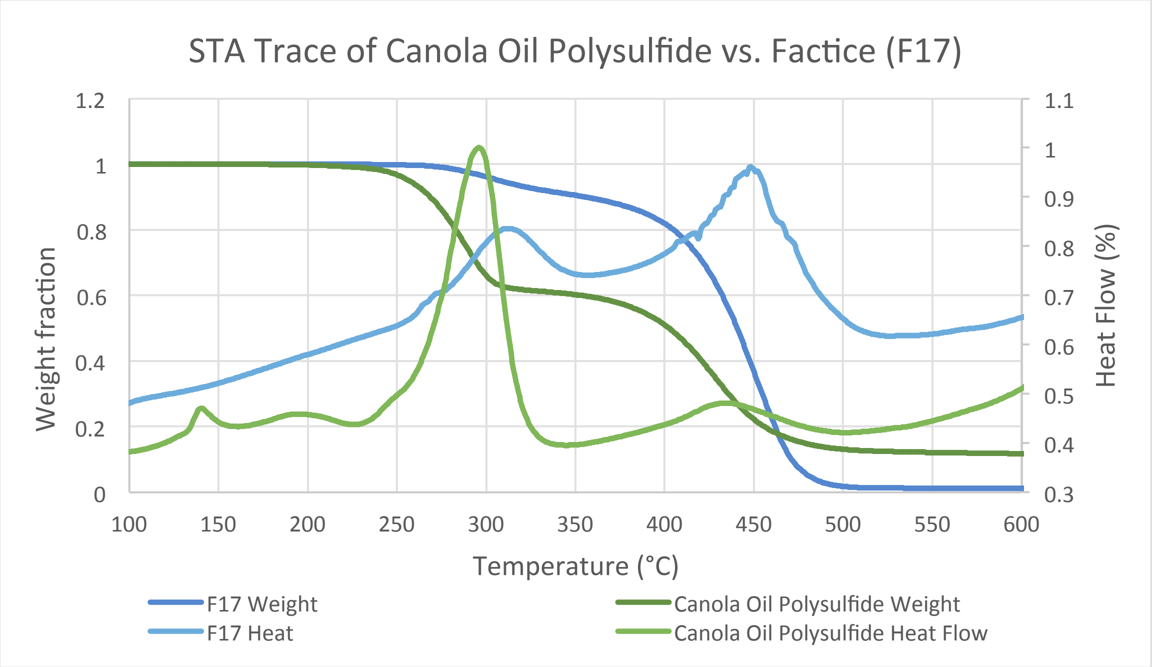

Fig. S30 | Simultaneous thermal analysis of the canola oil polysulfide (50% sulfur) prepared by inverse vulcanisation is plotted with factice F17 for comparison. We attribute the first major mass loss of the canola oil polysulfide to the thermal decomposition of the polysulfide domain.

Comparison of canola oil polysulfide prepared by inverse vulcanisation and classic vulcanisation. The canola oil polysulfide was prepared with 50% sulfur according to the standard inverse vulcanisation procedure (Fig. S1). For classic vulcanisation, canola oil (10.0 g) was heated to 180 ºC in a 250 mL round bottom flask with stirring. Sulfur (10.0 g) was then added in several portions over 5 minutes. The mixture was stirred vigorously for an additional 15 minutes, after which time the mixture reached its gel point and formed a brown rubber very similar in appearance to the product formed from inverse vulcanisation. STA of both samples revealed a similar decomposition and calorimetric profile.

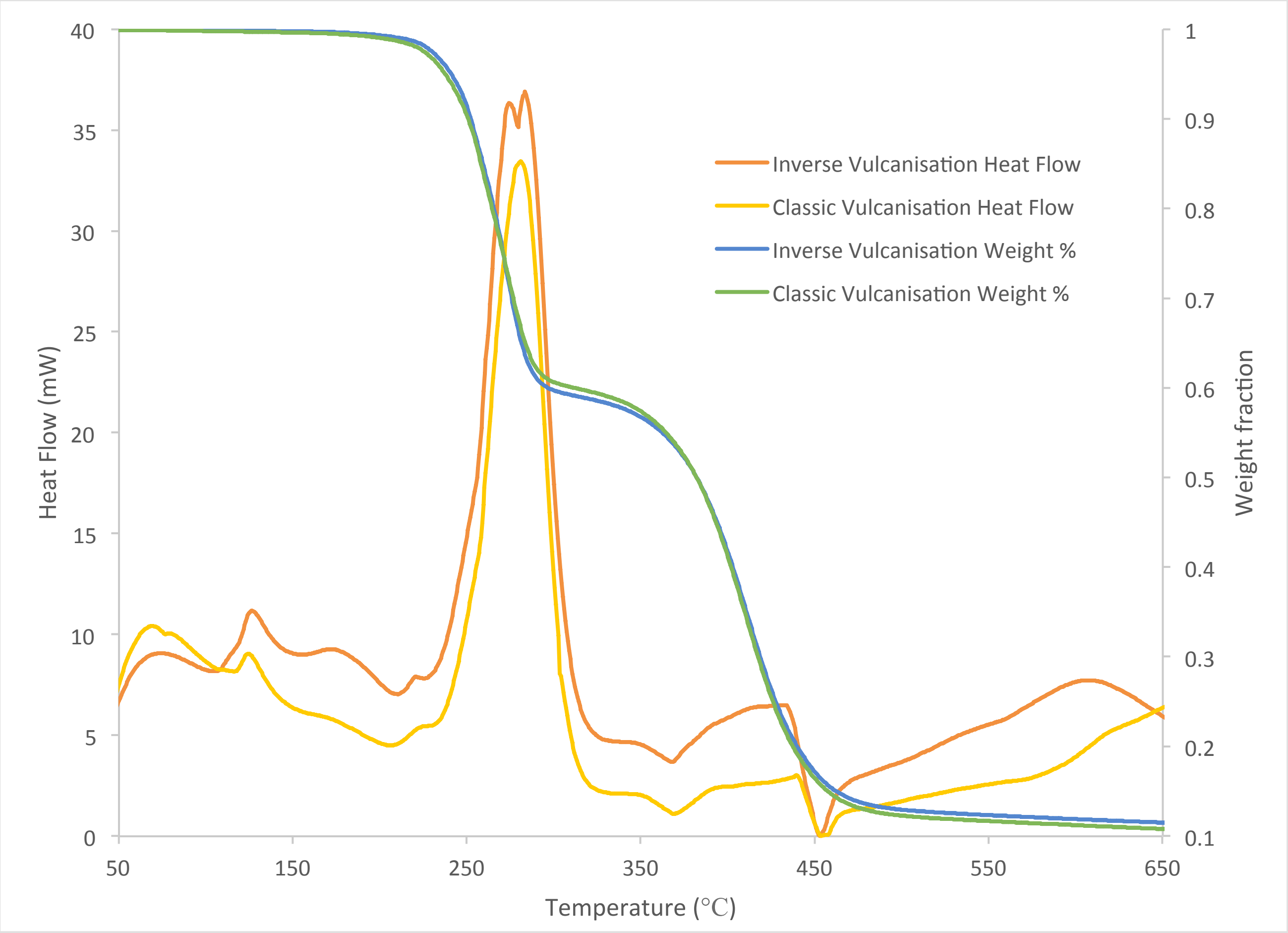

Fig. S31 | Thermogravimetric analysis and differential scanning calorimetry of the canola oil polysulfide prepared at 50% sulfur using inverse vulcanisation and classic vulcanisation.

DSC of canola oil polysulfide prepared by traditional vulcanisation and inverse vulcanisation

Differential scanning calorimetry was repeated, with a focus on the region where sulfur melts. Slightly more free sulfur was observed when using inverse vulcanisation (9% free sulfur) compared to traditional vulcanisation (8% free sulfur).

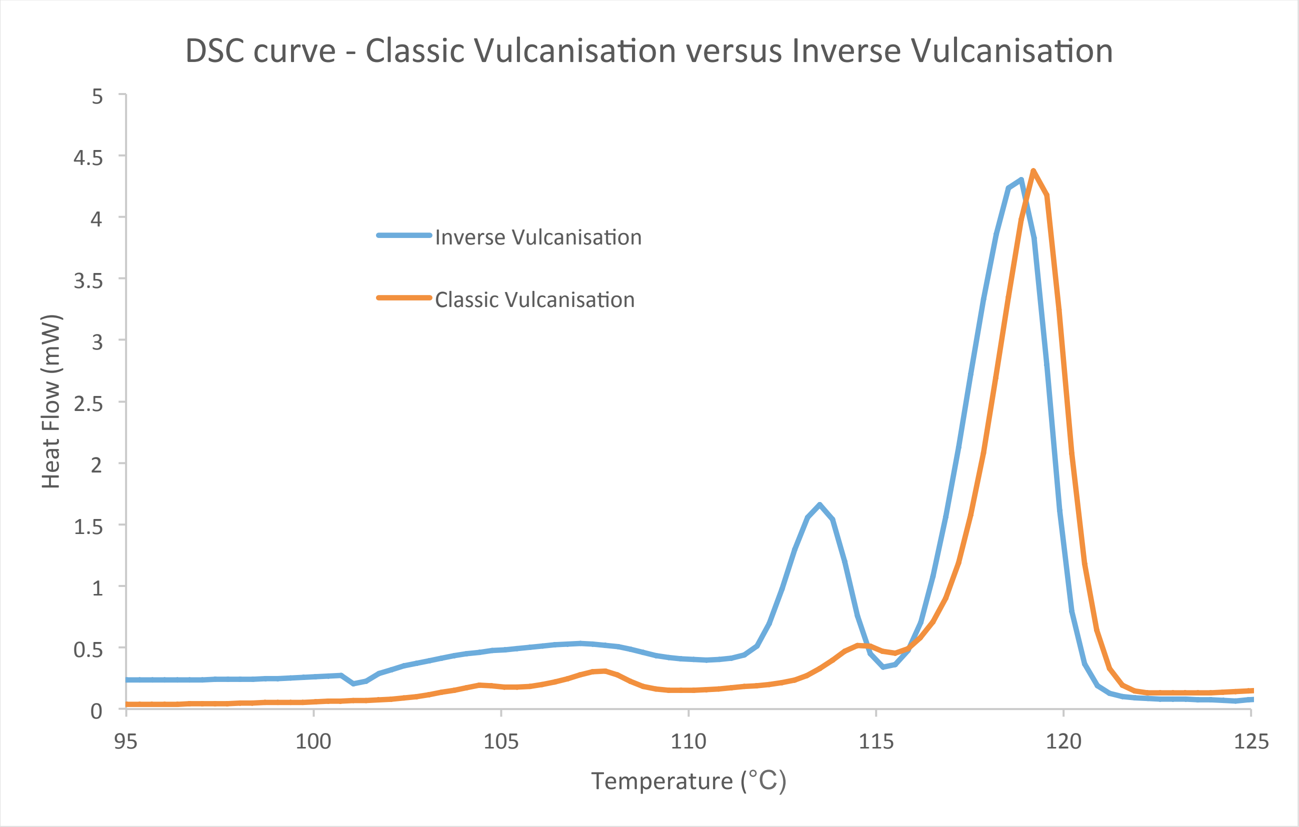

Fig. S32 | Differential Scanning Calorimetry of the canola oil polysulfide prepared at 50% sulfur using inverse vulcanisation and classic vulcanisation. The region of free sulfur is shown to illustrate a subtle difference in the materials.

Dynamic Mechanical Analysis of canola oil polysulfide (50% sulfur)

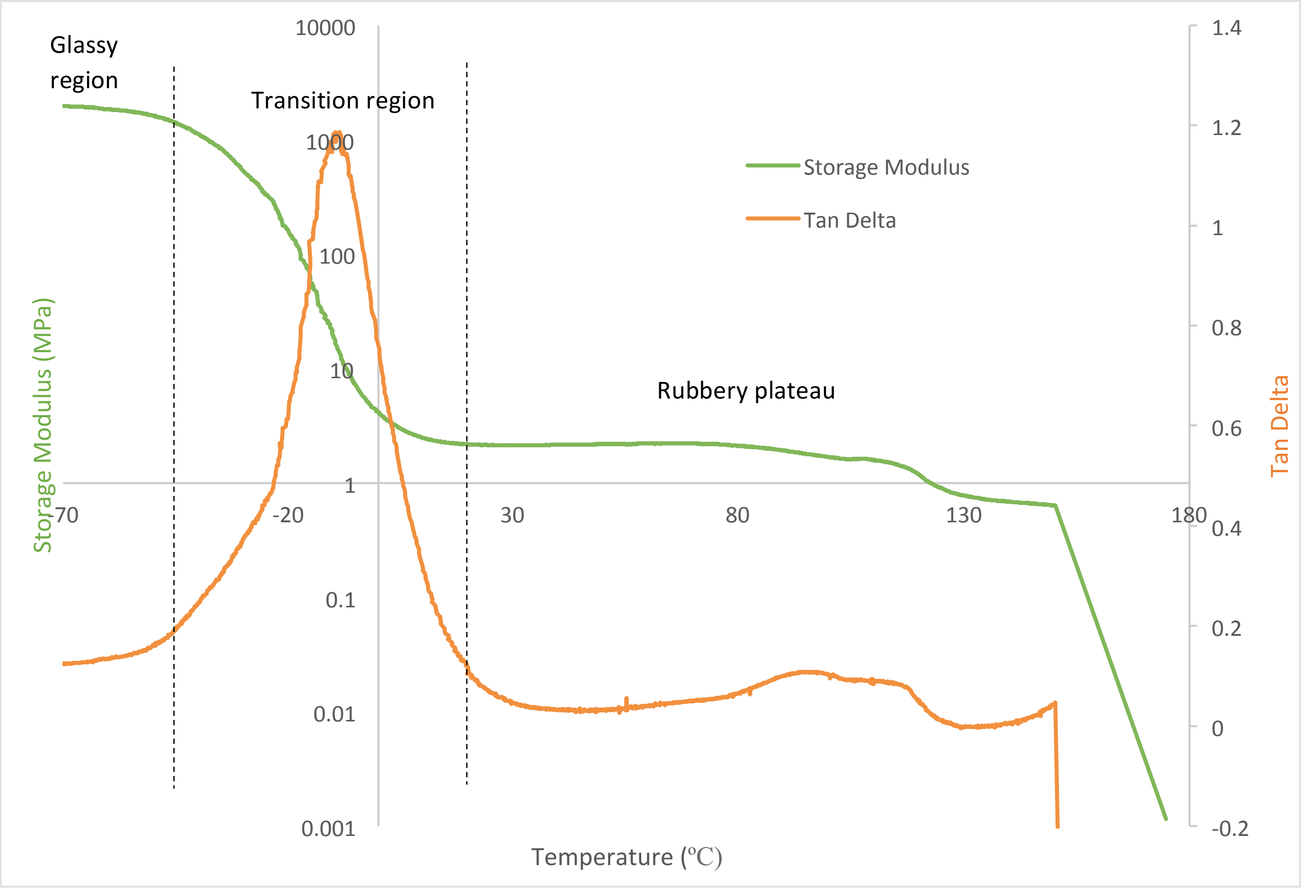

T_g measurements:

Storage Modulus drop onset	Tan Delta (Loss/Storage) Peak
-32 ºC	-9 ºC

Fig. S33 | Dynamic Mechanical Analysis was carried out in tension-mode using the Canola Oil Polysulfide prepared at 50% sulfur.

Determination of glass transition temperature by DSC for non-porous polysulfide (50% sulfur)

The glass transition temperature of the non-porous canola oil polysulfide was -12.2 ºC, as determined by DSC:

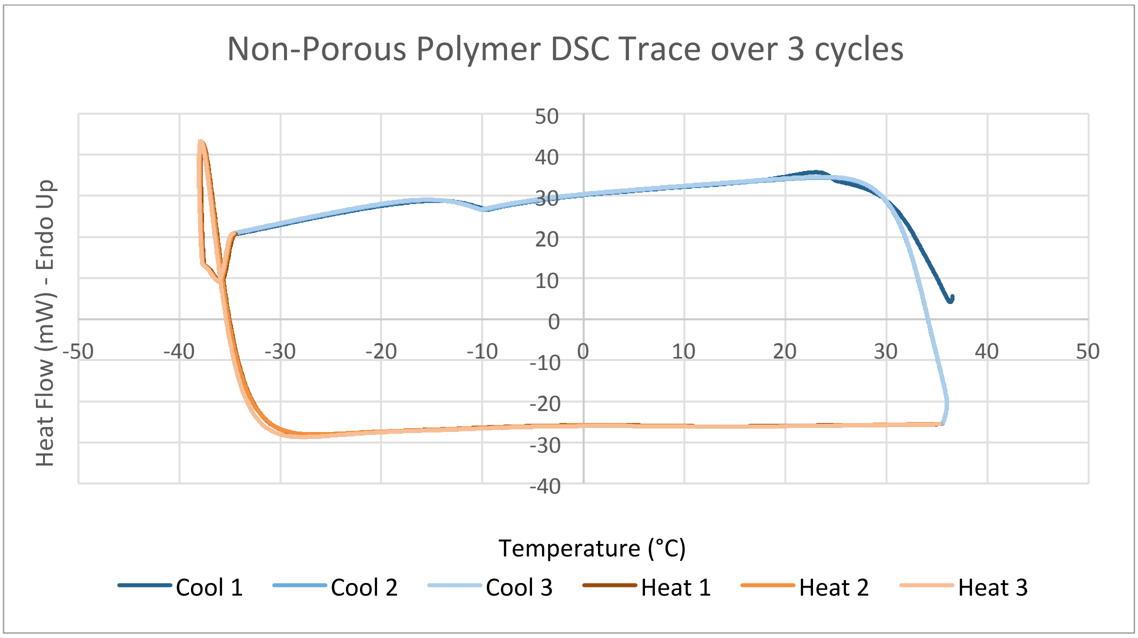

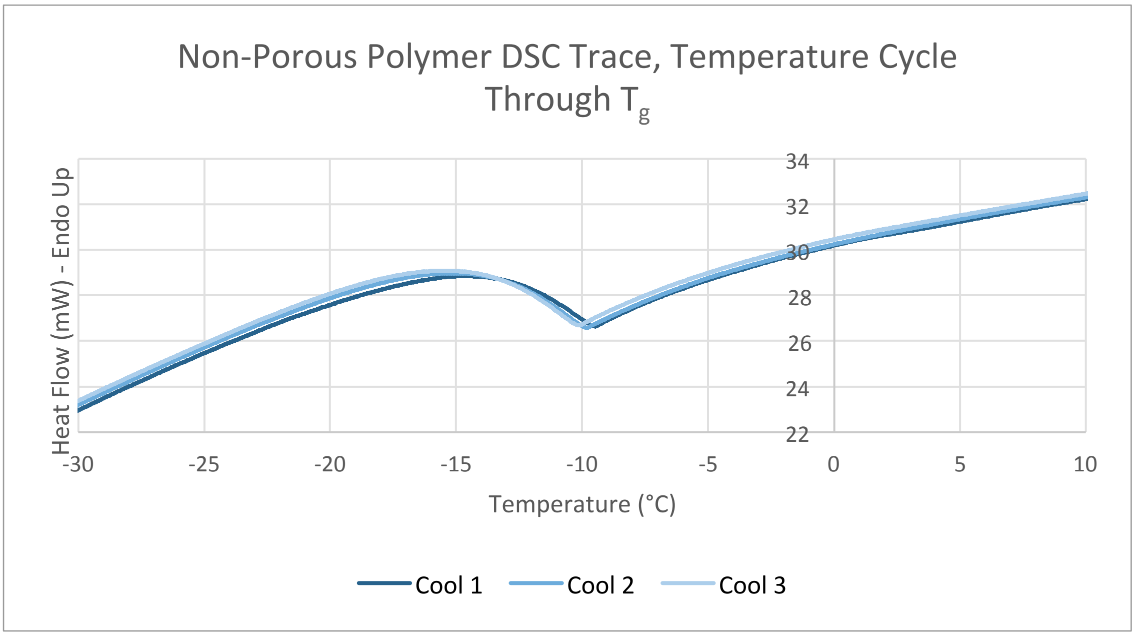

Fig. S34 | Determination of T_g using DSC for the non-porous Canola Oil Polysulfide prepared at 50% sulfur.

Canola Oil Polysulfide capture of mercury chloride from water

The Canola Oil Polysulfide (2.0 g, mixture of particles 2-12 mm in diameter) was added to a 20 mL glass vial, followed by 5 mL of a 20 mg/mL aqueous $HgCl_2$ solution (100 mg total $HgCl_2$). The mixture was incubated without stirring for 24 hours. A control sample containing just water and the polysulfide (and no $HgCl_2$) was also run in parallel. After the 24 hours, the polysulfide was isolated by filtration and washed with 3 aliquots of 5 mL deionised water. The aqueous solution was then transferred to a pre-weighed 50 mL round bottom flask and the water removed by rotary evaporation to provide unsequestered $HgCl_2$. The experiment was run in triplicate resulting in an average of 46 mg of $HgCl_2$ remaining in solution and 54 mg bound to the polysulfide. Notably, the polysulfide underwent a change in colour during the incubation, from brown to grey. No colour change was observed if mercury was not present.

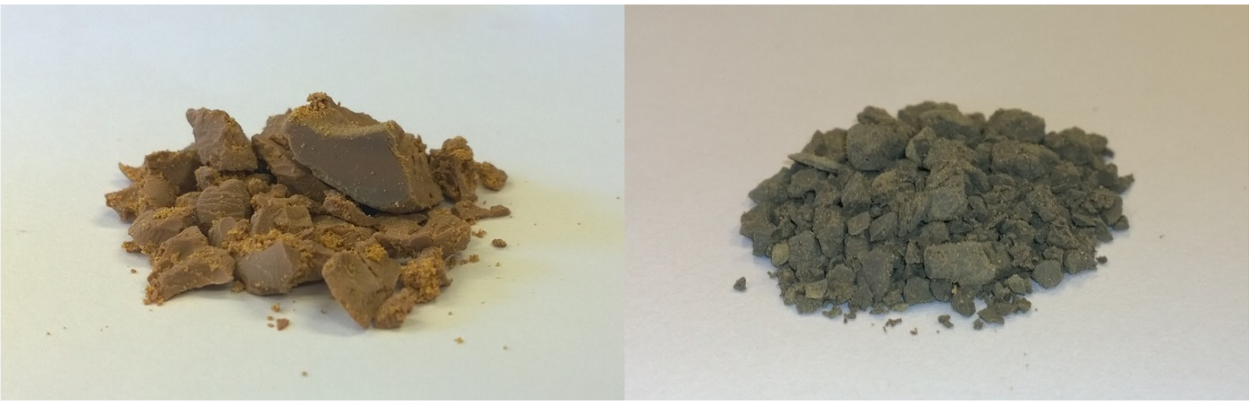

Fig. S35 | Canola oil polysulfide before and after treatment with mercury chloride
Left: Canola Oil Polysulfide synthesised before treatment with mercury chloride
Right: Canola Oil Polysulfide synthesised after treatment with mercury chloride (20 mg/mL, 24 hours).
The material changed colour (from brown to grey) after binding the mercury.

Effect of the amount of Canola Oil Polysulfide on the capture of mercury chloride from water. The procedure above was repeated with different quantities of Canola Oil Polysulfide: 250 mg, 500 mg, 1.00 g, 2.00 g, 4.00 g and 8.00 g. The volume and concentration of aqueous $HgCl_2$ remained the same for each sample (5 mL of a 20 mg/mL aqueous solution of $HgCl_2$), as did the incubation time (24 hours). As the mass of polysulfide increases, the mass of $HgCl_2$ remaining in solution after the 24 hour incubation decreases. This is likely because of the increased surface area available to bind to mercury. This experiment also indicates that the maximum amount of mercury chloride bound by weight for this particle size is about 4%. The results are tabulated below:

Effect of amount of Canola Oil Polysulfide on aqueous $HgCl_2$ capture

Mass Polysulfide (g)	$HgCl_2$ remaining (mg)	$HgCl_2$ sequestered (mg)	% $HgCl_2$ sequestered
0.25	91	9	9
0.50	82	18	18
1.00	60	40	40
2.00	42	58	58
4.00	23	78	78
**8.00**	**9**	**91**	**91**

Fig. S36 | Effect of Hg(II) concentration on Mercury(II) Capture:

The general procedure (see above) was repeated with different concentrations of mercury chloride: 20, 10 and 5 mg mL^{-1}. The volume of water (5.0 mL) and mass of polysulfide (2.00 g) remained the same for each sample, as did the incubation time (24 hours). There was not a substantial difference in mercury capture efficiency over this concentration range.

Effect of $HgCl_2$ concentration on aqueous $HgCl_2$ capture

Concentration $HgCl_2$ (mg mL^{-1})	Total $HgCl_2$ (mg)	$HgCl_2$ remaining (mg)	$HgCl_2$ sequestered (mg)	% $HgCl_2$ sequestered
5	25	6	19	76
10	50	18	32	63
20	100	38	62	62

Fig. S37 | Mercury(II) chloride capture at lower concentrations (measured by ICP-MS):

A solution of aqueous mercury chloride was made up to 3.5 ppm mercury (as measured by ICP-MS). 5 mL of this mercury chloride solution was incubated with 2 g of the polysulfide for 24 hours. After this time 1 mL liquid was filtered through a 0.2 µm syringe filter and the concentration of mercury remaining measured by ICP-MS. An average concentration of 0.35 ppm of mercury remained (average of triplicate experiments), indicating that 90% of the mercury was captured under these conditions.

SEM analysis of Canola Oil Polysulfide after treatment with mercury chloride

A 12.0 g sample of the Canola Oil Polysulfide (0.5 to 1.0 mm particles, as prepared above using sieves) was incubated in an aqueous solution of mercury chloride (30 mL of 20 mg/mL $HgCl_2$) for 24 hours. After this time, the polysulfide turned from brown to grey. The polysulfide was then filtered and washed with deionised water (3 × ~30 mL). The filtrate was concentrated under reduced pressure to provide 186 mg of unbound mercury chloride. Therefore, the polysulfide had captured 414 mg (or 70%) of the mercury. A sample of the mercury-treated polysulfide was then prepared for SEM and analysed.

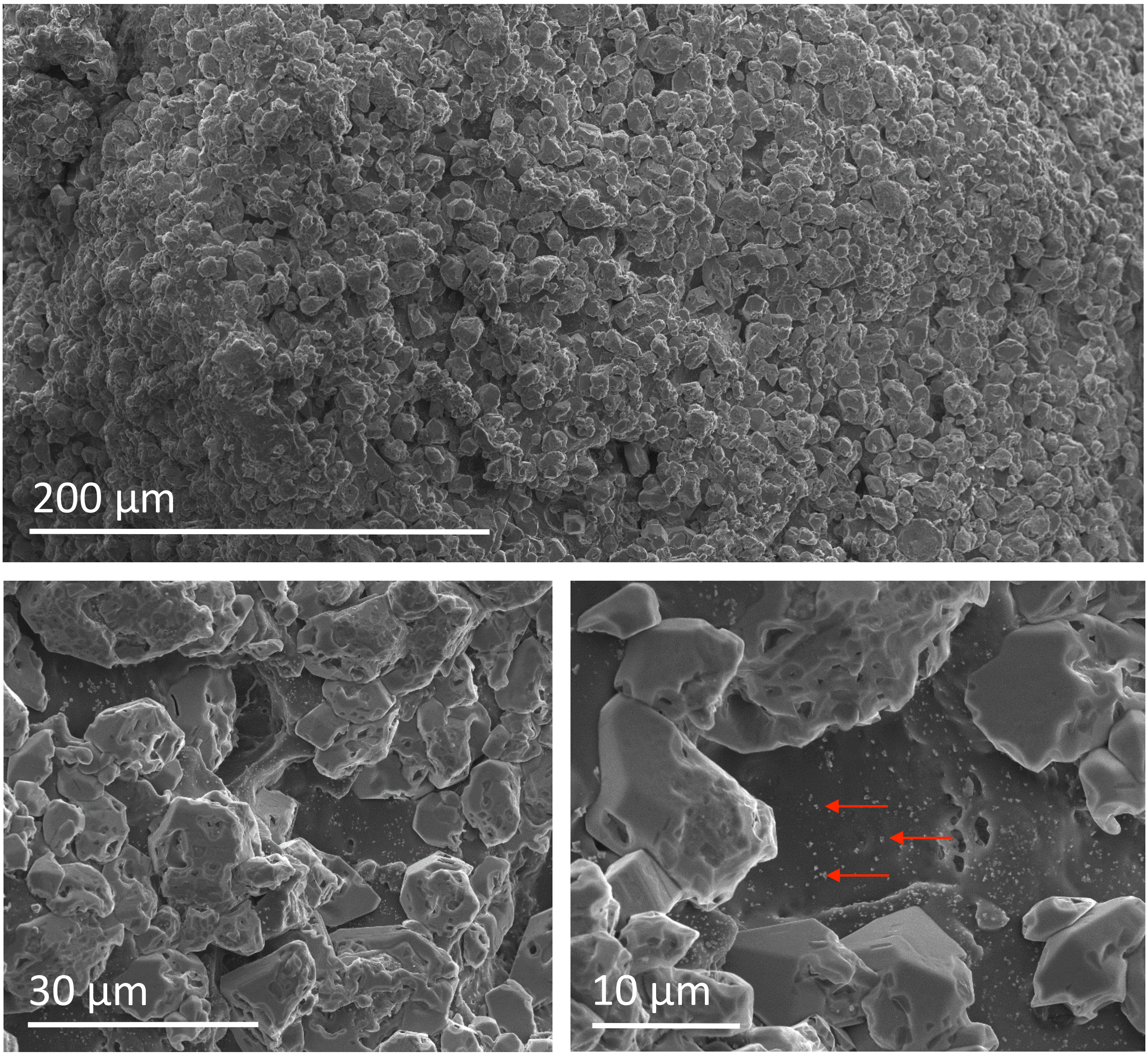

Fig. S38 | SEM of the mercury chloride treated polymer. Mercury rich nanoparticles were detected on the surface of the polymer (see red arrows for representative examples). The presence of mercury in these nanoparticles was verified by EDS (see next).

EDS analysis of mercury chloride-treated polysulfide surface

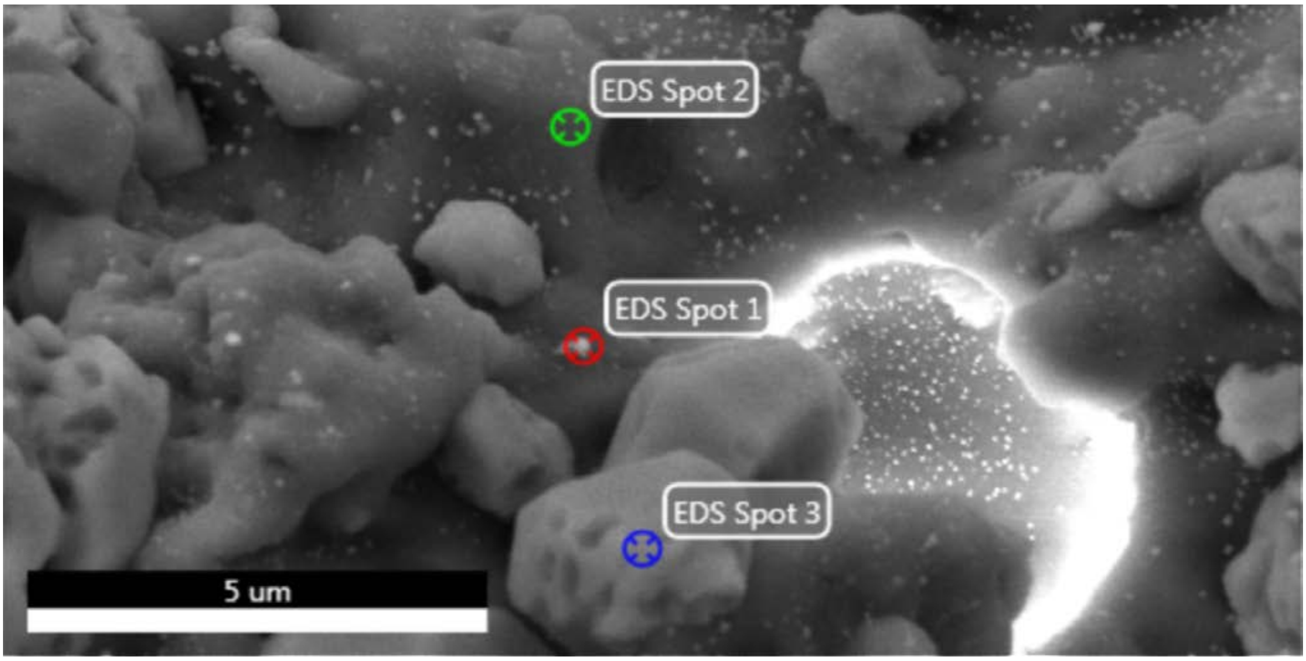

EDS Spot 1	EDS Spot 2	EDS Spot 3
Nanoparticles contain Hg	Smooth surface of polysulfide	Microparticle domain of polysulfide
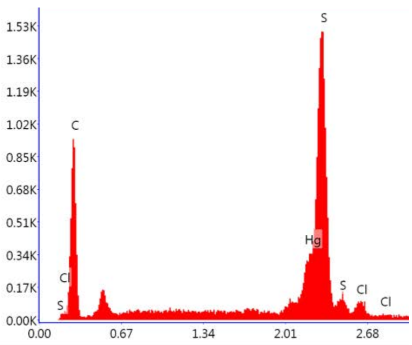	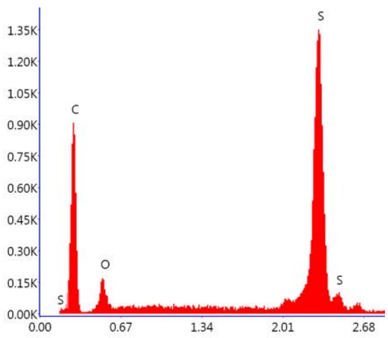	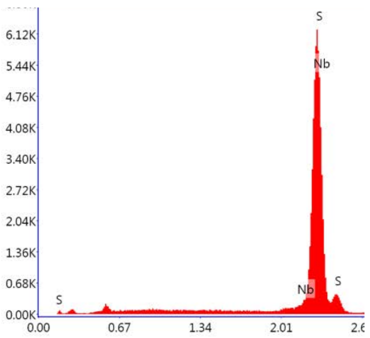
Mercury and chlorine were detected by EDS, indicating the nanoparticles are the product of mercury capture.	Unmodified domain of the canola oil polysulfide.	Sulfur rich domain of the canola oil polysulfide

Fig. S39 | SEM and EDS of the mercury chloride treated polymer. Mercury rich nanoparticles were detected on the surface of the polymer at Spot 1. The unmodified canola oil polysulfide is detected at Spot 2. A sulfur-rich particle was detected in Spot 3.

Mercury Leaching Study (mercury chloride)

1.0 g samples of mercury chloride-treated polysulfides were incubated in 10 mL milliQ water for 24 hours (2.2 mg total $HgCl_2$). The water was then tested by ICP-MS against an ICP standard of Hg in 2% HNO_3 (1% HNO_3 and 1% HCl in water used as a diluent) to determine the concentration of mercury that had leached from the polymer over this time. Tests were run in duplicate. Both samples were diluted 1/10 in a 1% HNO_3 and 1% HCl in water matrix. Samples were run in He mode to ensure ions flew monatomically.

Results of leaching:

**Sample**	**Conc. Hg (ppb) Leached into water**	**Description**
$HgCl_2$ (1)	0.51	$HgCl_2$-treated canola oil polysulfide (50% sulfur), 24 hour incubation in milliQ water – first replicate
$HgCl_2$ (2)	0.64	Replicate
**Average**	0.57	
Water	0.24	milliQ water (control)
Polysulfide	0.30	Untreated canola oil polysulfide (50% sulfur) 24hr incubation water (control)

This result indicates that leaching into water is negligible. If all mercury chloride were leached from the polymer, a concentration of 0.22 mg/mL or 220,000 ppb would be measured. An average of only 0.57 ppb Hg^{2+} was detected in the leachate.

Fig. S40 | Leaching study of mercury chloride, bound to the canola oil polysulfide (50% sulfur).

Canola Oil Polysulfide reactive capture of liquid mercury metal [$Hg^{(0)}$]

The Canola Oil Polysulfide (1.00 g, mixture of particles 2-12 mm in diameter) and 100 mg elemental mercury were added to a glass vial containing 7 mL deionised water. The mixture was stirred vigorously for 24 hours at room temperature. After this time, no elemental mercury was visible and the polysulfide had changed colour from brown to black. The colour change occurred after approximately four hours of vigorous stirring at room temperature. The colour change correlates with mercury capture and occurs on the surface of the particle. After the 24 hours of stirring, the black polymer-bound mercury was isolated by filtration and dried to constant mass. A mass of 1.099 g of this material was isolated, indicating good mass balance in the mercury capture (e.g. > 99% of the mercury reacted with the polysulfide).

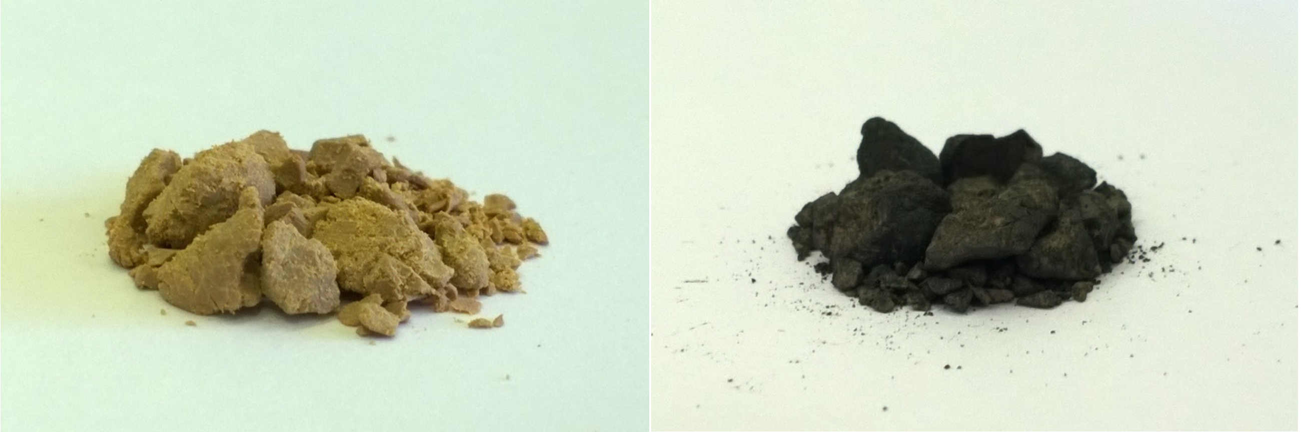

Left: Canola Oil Polysulfide
Right: Reaction product of the polysulfide with mercury metal.

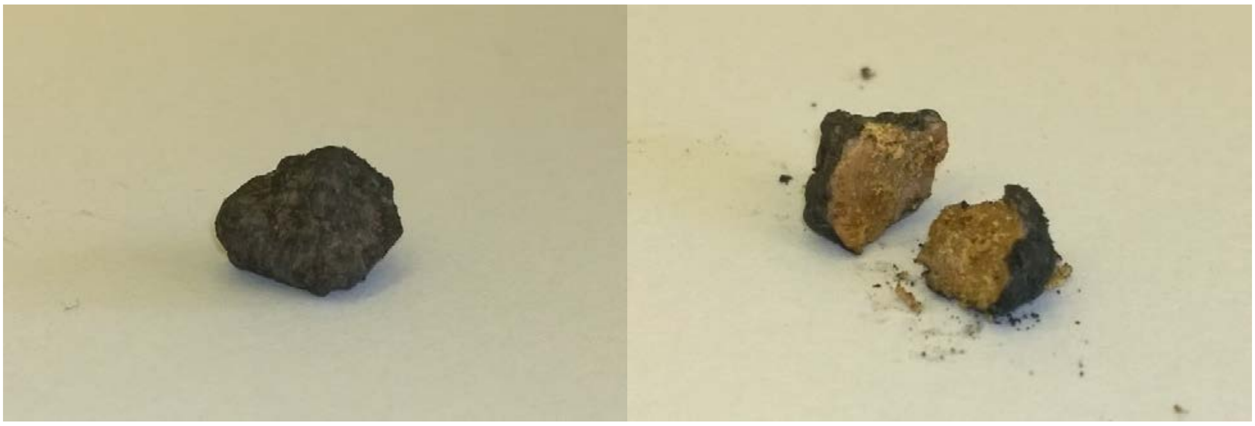

Left: Particle of the polysulfide after reaction with mercury metal
Right: Severed particle reveals that mercury is bound only to surface of particle.

Fig. S41 | The surface of the canola oil polysulfide (50% sulfur) reacts with mercury metal, forming a black product.

EDS analysis of elemental mercury-treated polysulfide surface

The Canola Oil Polysulfide (50% sulfur) and elemental mercury were added to a glass vial containing 7 mL DI water and reacted as described previously. After the reaction (24 hours, vigorous stirring), no elemental mercury was visible and the polysulfide had changed colour from brown to black. The polysulfide was isolated by filtration and then a 10 mm particle was cut in half. The cross-section was profiled by SEM and EDS, revealing the mercury was bound only to the surface, where the material appeared black.

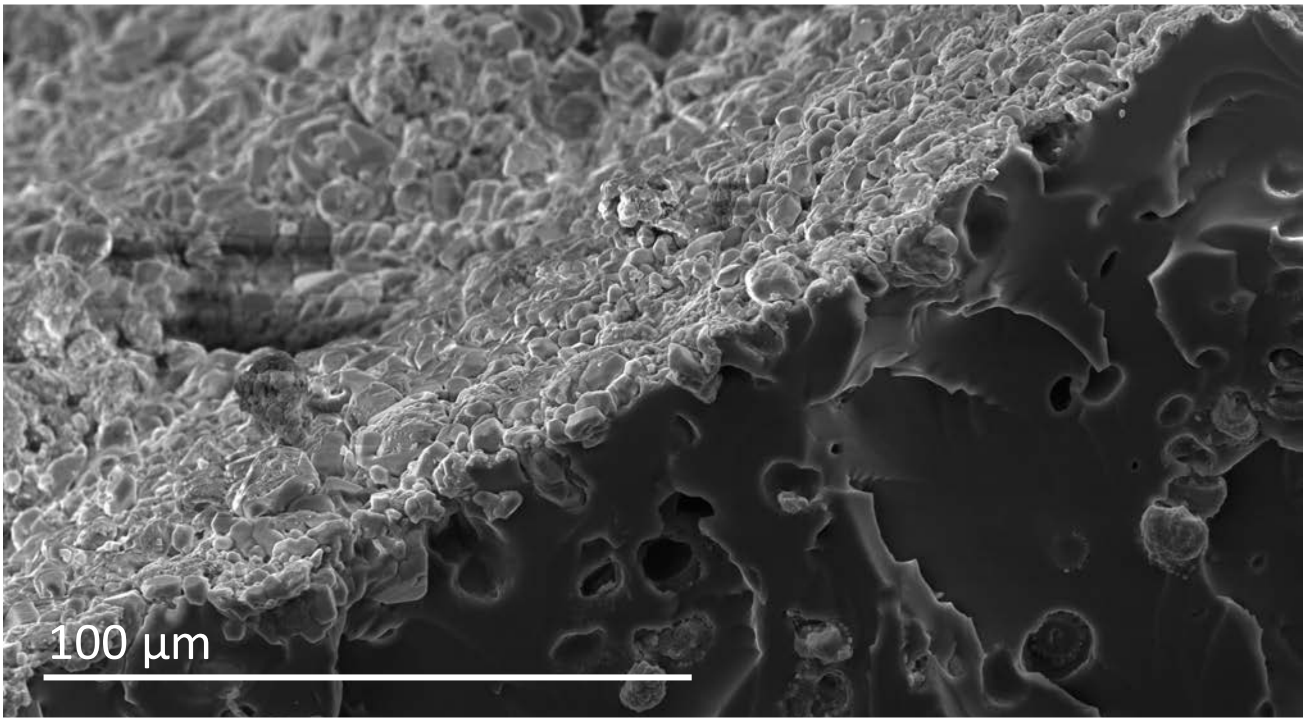

SEM image of polysulfide cross-section. Upper left: surface, lower right: interior

SEM: Cross section | EDS: Sulfur map | EDS: Mercury map

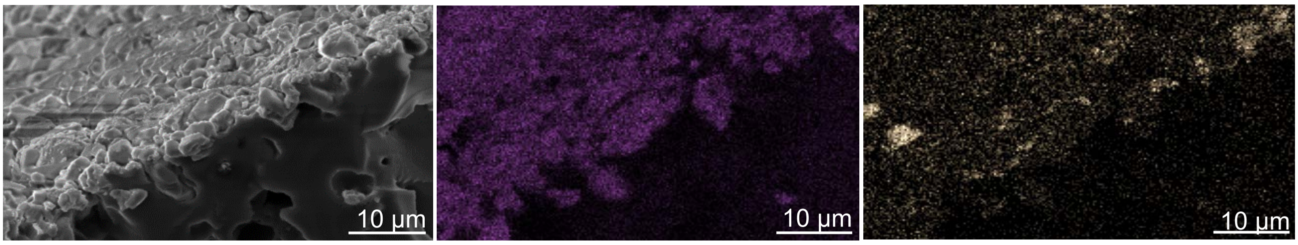

Cross section

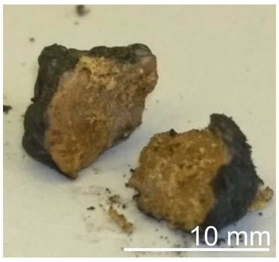

Fig. S42 | The surface of the canola oil polysulfide (50% sulfur) reacts with mercury metal, forming a black product. EDS analysis verifies mercury is found on the surface of the material.

Auger analysis of canola oil polysulfide after treatment with mercury metal

Elemental Hg treated Canola Oil Polysulfide

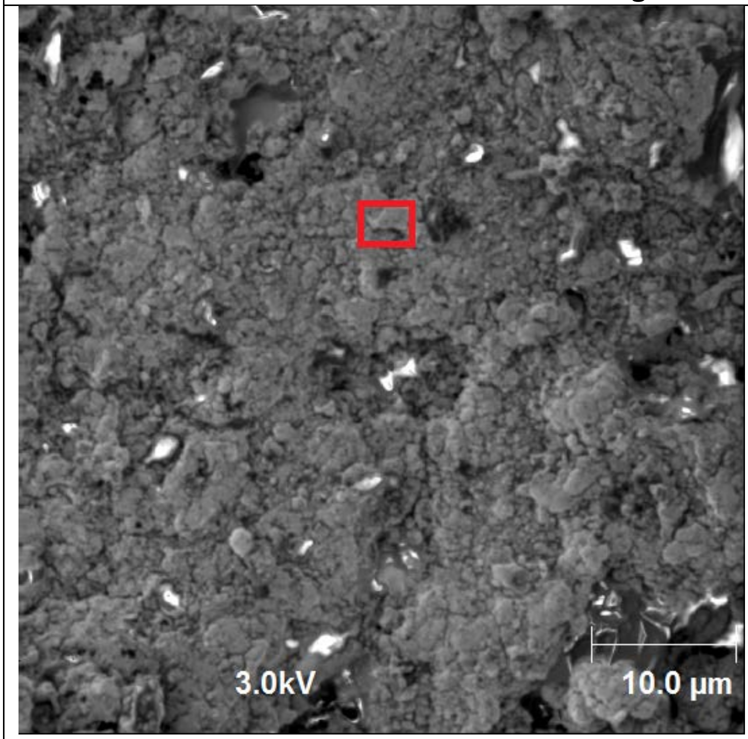

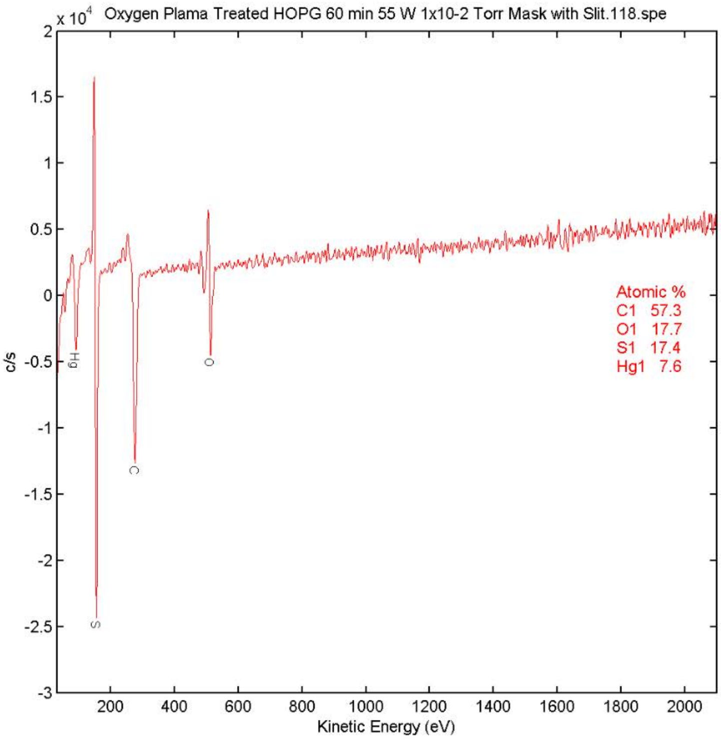

SEM Image	Auger Response

Fig. S43 | The surface of the canola oil polysulfide (50% sulfur) reacts with mercury metal, forming a black product. Auger analysis verifies mercury is found on the surface of the material.

XPS analysis of mercury-treated canola oil polysulfide before and after mercury capture

a. Canola Oil Polysulfide

Name	Position	% Conc.
O 1s	532.5	8.777
C 1s	285	83.431
S 2p	164.5	7.792

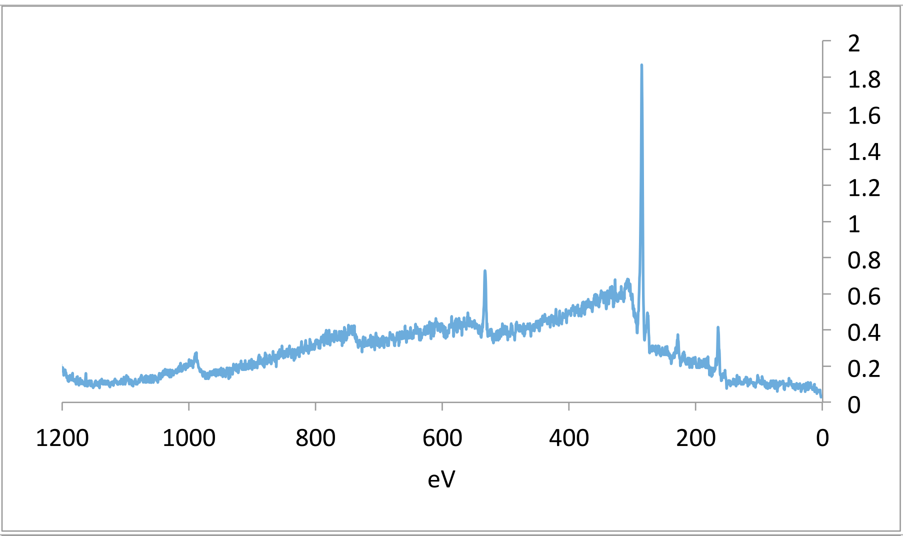

b. $HgCl_2$-treated Canola Oil Polysulfide

Name	Position	% Conc.
C 1s	285	79.393
S 2p	163.5	4.505
Hg 4f	102	0.312
Cl 2p	200	1.886
O 1s	532	13.904

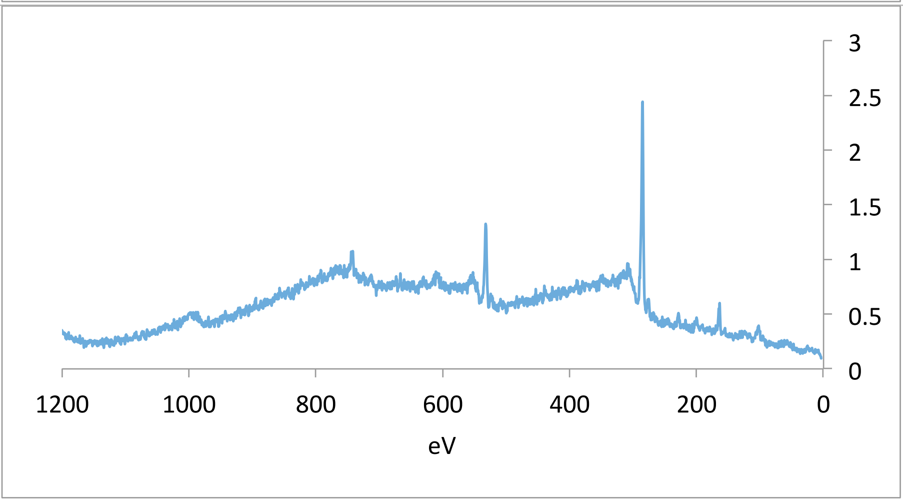

c. Hg^0-treated Canola Oil Polysulfide

Name	Position	% Conc.
O 1s	533	8.458
C 1s	285	86.992
S 2p	164	4.383
Hg 4f	101	0.167

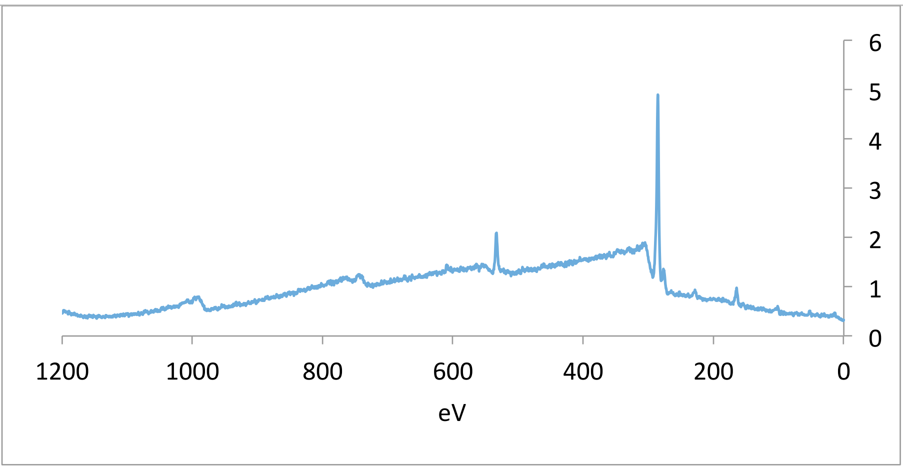

Fig. S44 | XPS analysis of the canola oil polysulfide revealed the mercury '4*f*' photoelectron peak for both the mercury chloride capture (b) and mercury metal capture (c). The observed binding energy is associated with mercury bound to sulphur (~101eV for HgS) for both samples. In the case of $Hg^{(0)}$ capture, this is consistent with oxidation of mercury to metacinnabar.

XRD Sample Preparation 1.24 g elemental mercury was added to a 50 mL centrifuge tube containing 2.47 g sulfur and mixed for 24 hours using an end-over-end mixer. Similarly, 2.47 g of canola oil polysulfide (50% sulfur, < 0.5 mm particle size) was mixed with 1.52 g elemental mercury in an end-over-end mixer for 24 hours. Unreacted sulfur, unreacted polysulfide, as well as those samples reacted with elemental mercury, were all ground to a fine powder using a mortar and pestle in preparation for loading on an XRD sample stage. The XRD spectra obtained for both reactions was metacinnabar, as it was identical to previously published XRD spectra.[2] It can therefore be concluded that the black material that results from the reaction of mercury metal and the S-S bonds of the canola oil polysulfide is metacinnabar.

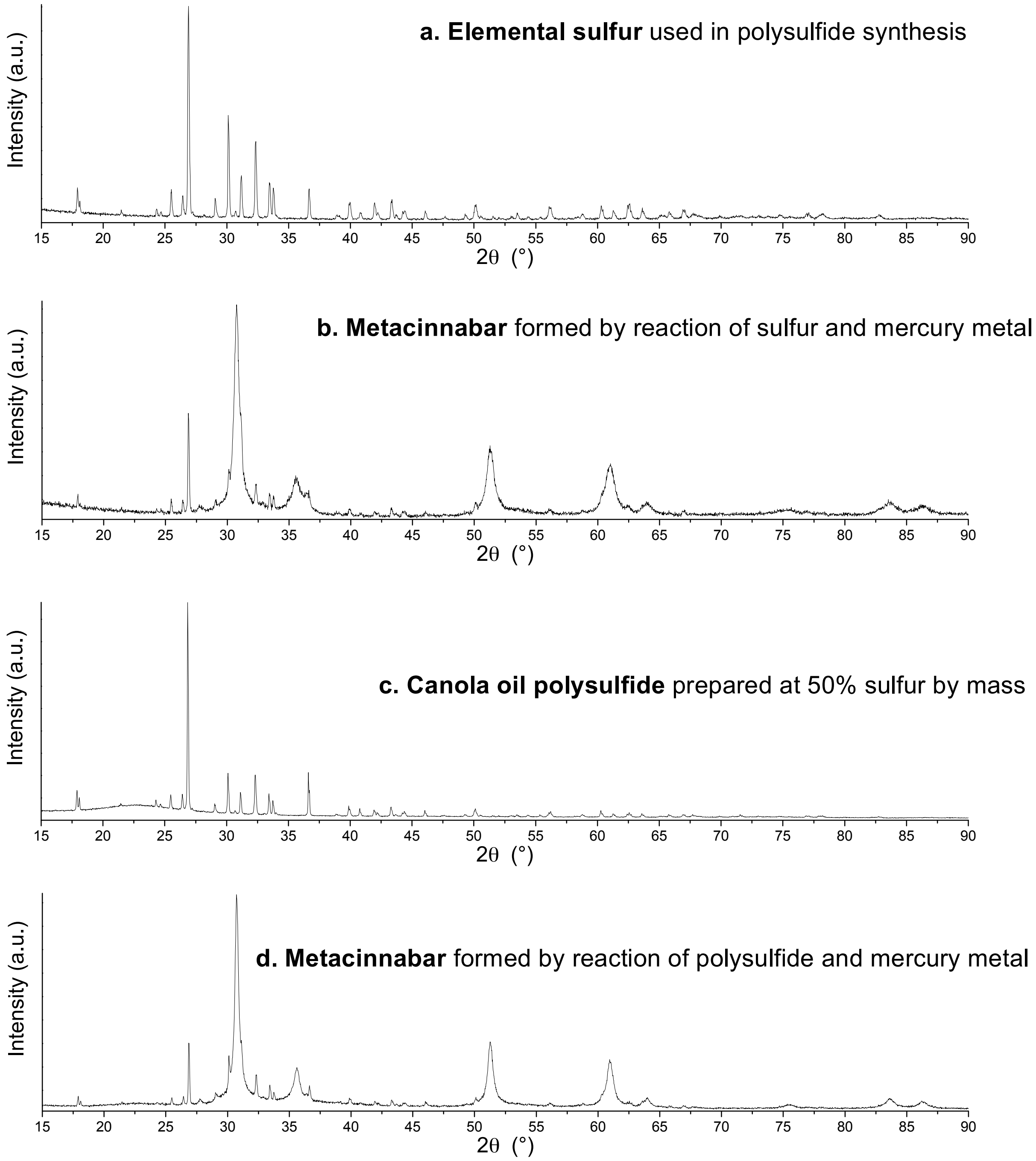

Fig S45 | XRD scans of **a,** elemental sulfur, **b,** metacinnabar prepared by the reaction of sulfur and mercury metal **c,** canola oil polysulfide (50% sulfur) and **d,** metacinnabar formed by reaction of polysulfide and mercury metal.

Mercury capture using polysulfide prepared from recycled cooking oil

1.0 g of the polysulfide (50% sulfur) prepared from recycled cooking oil (Fig. S7) was placed in a 25 mL round bottom flask equipped with a stirrer bar, along with elemental mercury (171 mg) and 10 mL DI water. The flask was sealed and the mixture stirred for 24 hours. During this time the polysulfide turned black, and some unreacted elemental mercury was still visible. The polymer and mercury were separated by mixing with equal volumes of hexane and water. The polymer remained at the phase boundary and the mercury settled to the bottom of the aqueous phase. The water and mercury were isolated, and separated from the polymer. The mercury was then separated from the water by transferring to a separatory funnel and diluting with dichloromethane. The mercury-dichloromethane mixture was then isolated and the dichloromethane evaporated in a fume hood. The mass of the unreacted mercury was recorded.

Mercury capture using Factice F17 (D.O.G.)

2.8 g of F17 grad D.O.G. Factice was placed in a 25 mL round bottom flask equipped with a stirrer bar, along with elemental mercury (217 mg) and 10 mL DI water. The flask was sealed and the mixture stirred for 24 hours. During this time the factice darkened in colour, and some unreacted elemental mercury was still visible. The factice and unreacted mercury were separated by mixing with equal volumes of hexane and water. The polymer remained at the phase boundary and the mercury settled to the bottom of the aqueous phase. The water and mercury were isolated, and separated from the polymer. The mercury was then separated from the water by transferring to a separatory funnel and diluting with dichloromethane. The mercury-dichloromethane mixture was then isolated and the dichloromethane evaporated in a fume hood. The mass of the unreacted mercury was recorded.

Sample	Polymer mass (g)	Sulfur mass (g)	Hg mass (mg)	Time (hours)	Hg removed (mg)
Factice F17	2.8	0.50	217	24	117
Polysulfide from Recycled cooking oil	1.0	0.50	171	24	116

Fig. S46 | Factice F17 (17% sulfur) and a polysulfide prepared from recycled cooking oil (50% sulfur) were compared in their reaction with mercury metal. An amount of polymer was added such that the mass of sulfur was the same. Both samples captured virtually the same amount of mercury metal, suggesting that the amount of mercury that can react corresponds to the amount of sulfur in the polysulfide. This result also suggests that the polysulfides in factice can react with mercury metal and that free sulfur is not required.

Sensitivity of chromogenic response of canola oil polysulfide in its reaction with mercury metal

In order to test the sensitivity of the polysulfide's response to elemental mercury, quantities of mercury ranging from 72 to 285 mg were added to 10 and 20 g quantities of polysulfide in separate 50 mL centrifuge tubes (Fig. S47). The polymer-mercury mixtures were rotated on a lab rotisserie for 24 hours and any changes to the mixture recorded. In all cases the polymer turned black, indicating reaction of mercury with the polysulfide. Given the intensity of the colour change, it is presumed that the polymer may also turn black when exposed to lesser quantities of elemental mercury than shown here. Because of the difficulties in measuring small quantities of metallic mercury, this experiment was not pursued further. From these results we can conclude that mercury can be detected by visual inspection after the reaction of mercury and the canola oil polysulfide at ratios of 3.6 mg of mercury per gram of polymer or lower.

Polymer (g)	10.0	10.0	10.0	20.0	20.0	20.0	10.0
$Hg^{(0)}$ (mg)	285	207	75	121	72	216	0
Result:	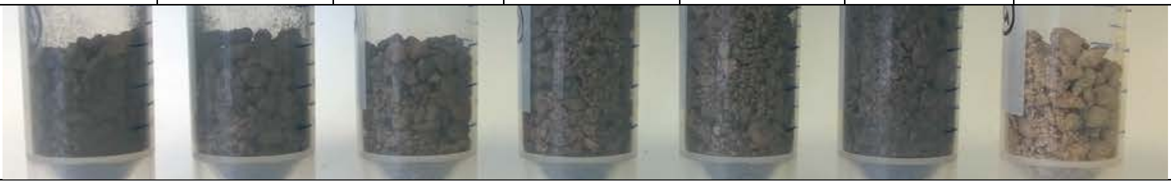						

Fig. S47 | Preliminary study of the sensitivity of the canola oil polysulfide in its detection of metallic mercury

Mercury flour preparation and SEM and EDS analysis

Loam was obtained from Glenalta, South Australia and ground with a mortar and pestle before passing through a sieve to obtain a soil with particle size less than 0.50 mm. 5.00 g of this powdered soil was sealed in a 50 mL centrifuge tube with 200 mg elemental mercury and rotated (30 rpm) on an end-over-end mixer for 24 hours. After this time the mercury was no longer visible to the naked eye, having been dispersed throughout the soil. There was no visible difference between the soil before or after treatment with mercury.

Soil	Soil milled with mercury metal: “Simulated Mercury Flour”
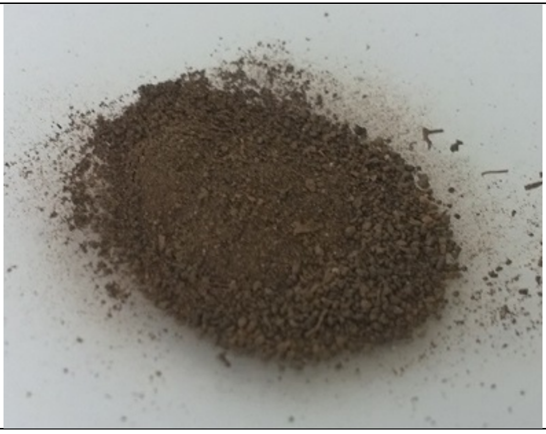	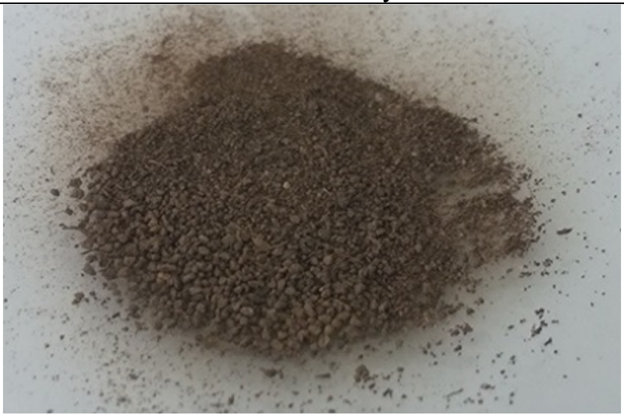
Loam, prepared at a particle sizes < 0.50 mm	Soil milled with mercury appeared highly similar to the untreated soil

Fig. S48 | Soil (left) and simulated mercury flour (right, 4% Hg by mass) appear similar to the naked eye

The soil and simulated mercury flour were analysed by SEM and EDS:

<table>
<tr><th>Soil</th><th>Simulated mercury flour</th></tr>
<tr><td>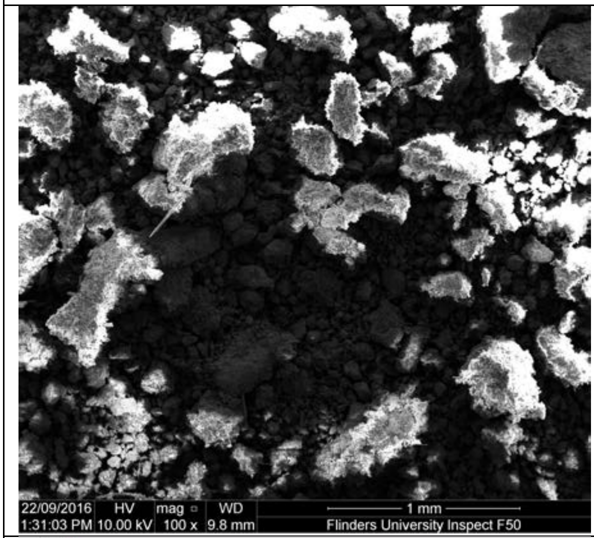
</td><td>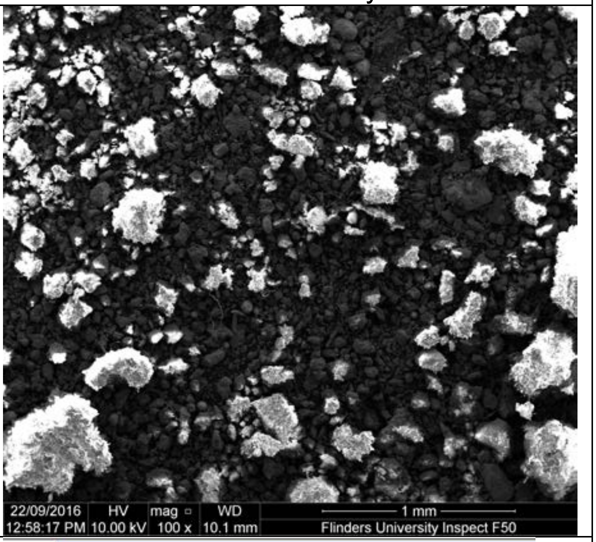
</td></tr>
<tr><td>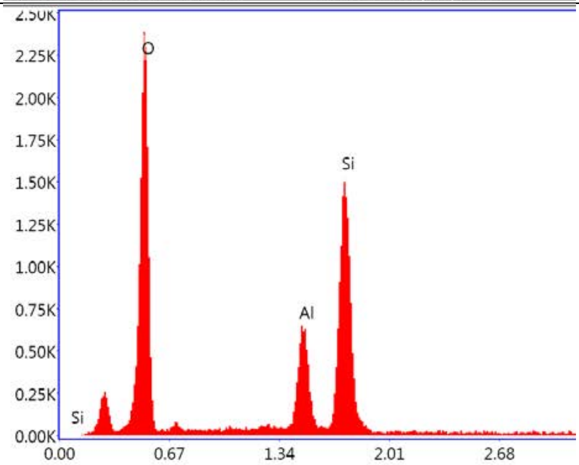
</td><td>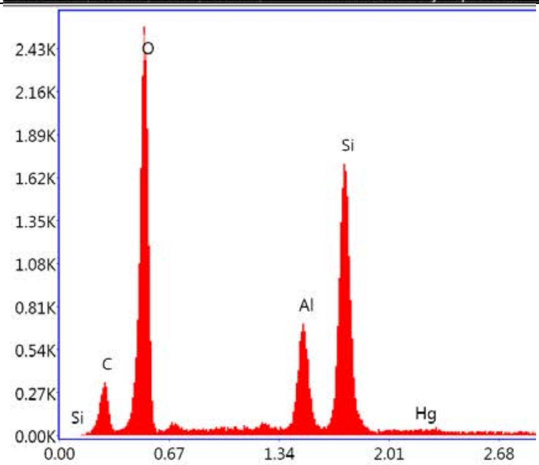
</td></tr>
<tr><td>Soil, sieved to a fine powder of particles no greater than 0.50 mm in diameter</td><td>EDS scans over an area of approx. 10 mm^2 of the simulated mercury flour did not return a clear indication of mercury due to the formation of very small microspheres of mercury that are difficult to detect.</td></tr>
</table>

Fig. S49 | In a cursory SEM and EDS scan, it is difficult to detect mercury in the simulated mercury flour.

Simulated Mercury Flour

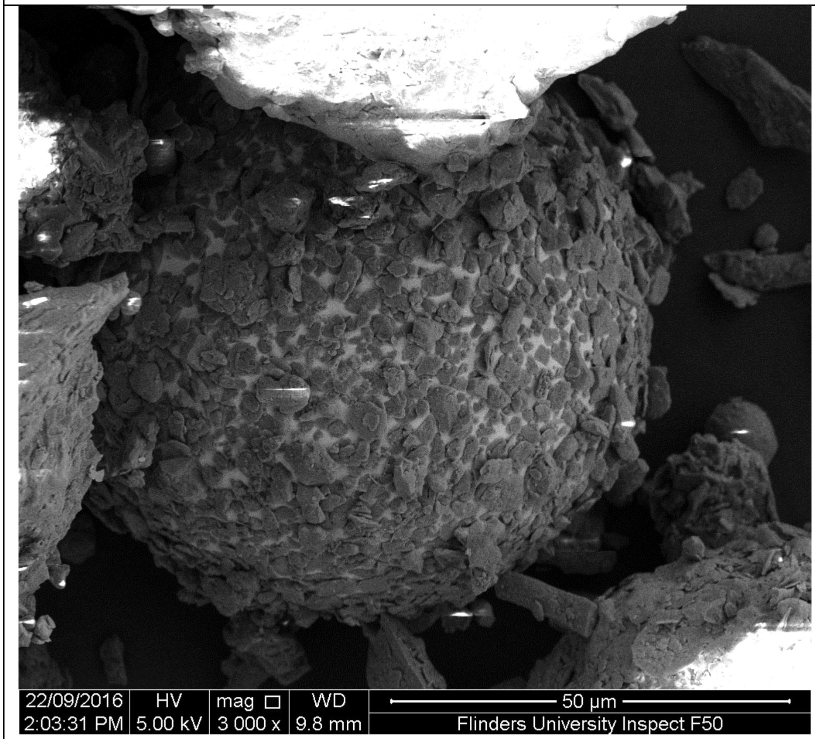

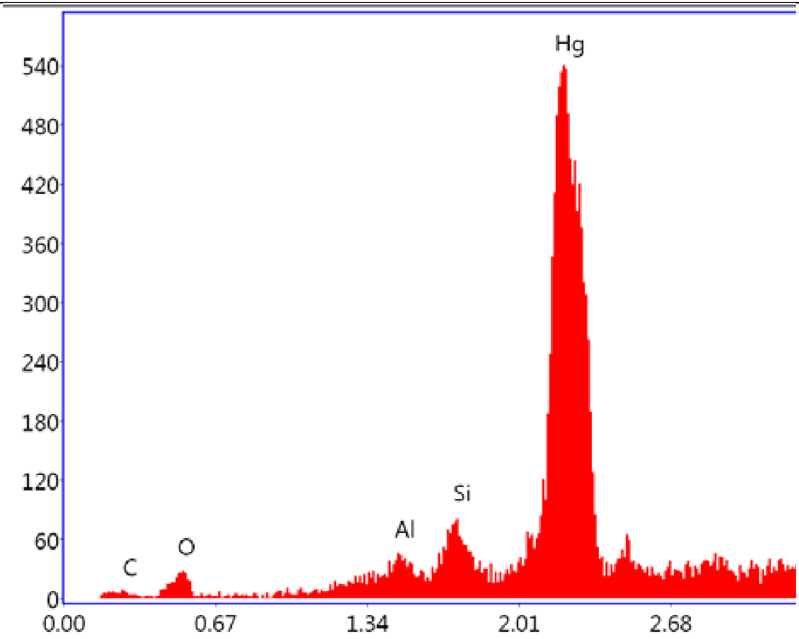

Fig. S50 | SEM and EDS analysis of mercury flour. After thorough searching, mercury was detected as microspheres dispersed in the soil. This "floured mercury" is covered in micro- and nanoparticles of soil. The soil prevents the mercury from coalescing.

Microparticle of mercury, coated in soil particles

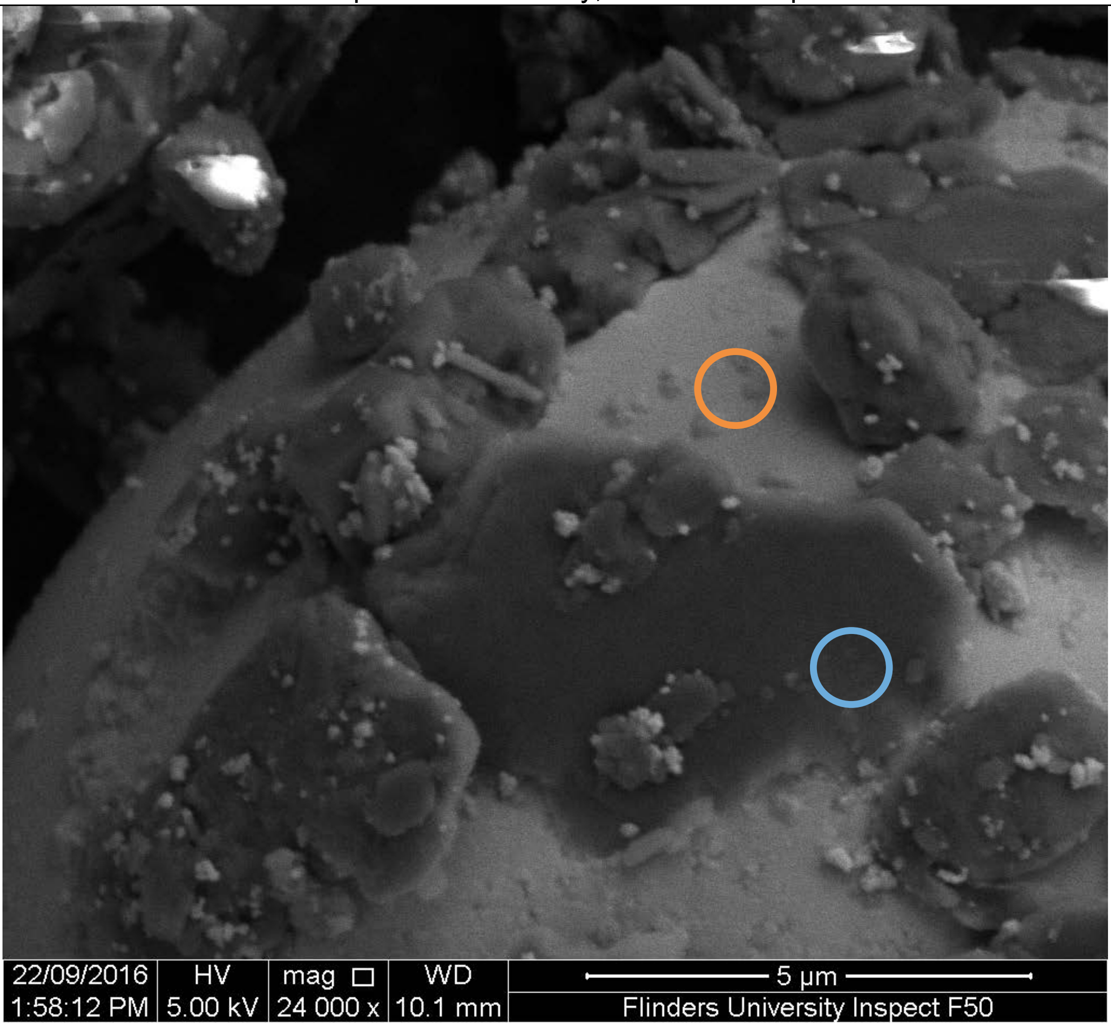

EDS Spot 1

Microparticle of mercury, coated in nanoscale soil particles

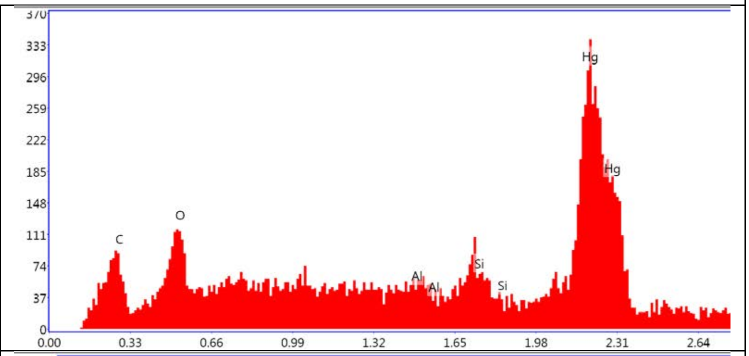

EDS Spot 2

Soil particle, adsorbed to the surface of a mercury microparticle

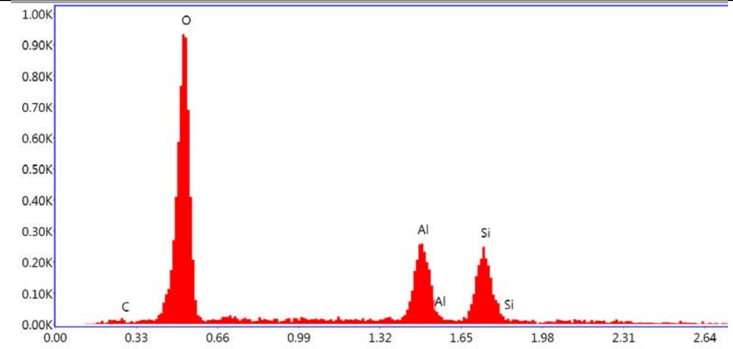

Fig. S51 | SEM and EDS analysis of mercury flour. The SEM image reveals micro- and nanoparticles of soil adhering to the surface of the mercury microsphere.

Capturing mercury flour using the non-porous canola oil polysulfide

5.0 g canola oil polysulfide (50% sulfur) of a particle range of 2.5 – 5.0 mm was isolated using a sieve. These particles were added to 5.0 g of the simulated mercury flour and mixed in a 50 mL centrifuge tube on an end-over-end mixer for 24 hours. A control sample treated identically but without the addition of mercury was also prepared for comparison. Over this time, the polymer in the presence of mercury turned black, indicating reaction with the mercury flour. The polymer in the soil in which no mercury was added remained brown. The polymer particles were then separated from the bulk of the soil using a sieve. EDS analysis clearly indicated that mercury was bound to the polymer. This experiment demonstrates that the canola oil polysulfide, prepared as a particle, can capture mercury from soil and then be isolated using a sieve.

The isolated polymer after incubation with mercury-treated soil: After mixing with the mercury flour, the polymer changed from brown to black, indicating reaction with the elemental mercury.

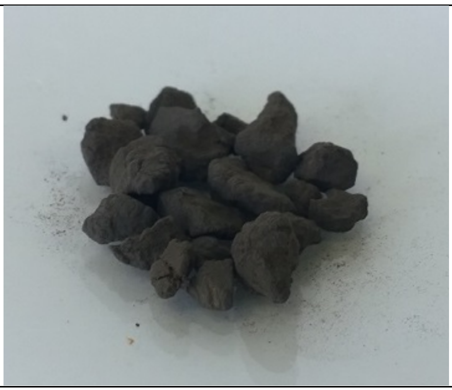

The isolated polymer from the control sample (soil, but no mercury). After mixing with just soil, the polymer remains its original brown colour

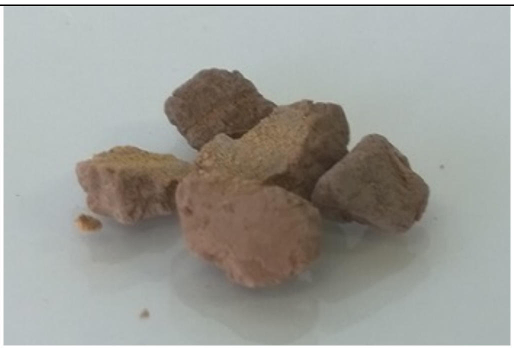

Fig. S52 | The canola oil polysulfide reacts with mercury flour**.** The top image is the black polymer, isolated from the soil using a sieve. The black colour is consistent with reaction with the mercury flour. In a control experiment (bottom), the polymer retains its brown colour after mixing with soil that does not contain mercury.

Control polymer<br>(isolated from soil with no mercury)	Polymer, post treatment<br>(isolated after reaction with mercury flour)
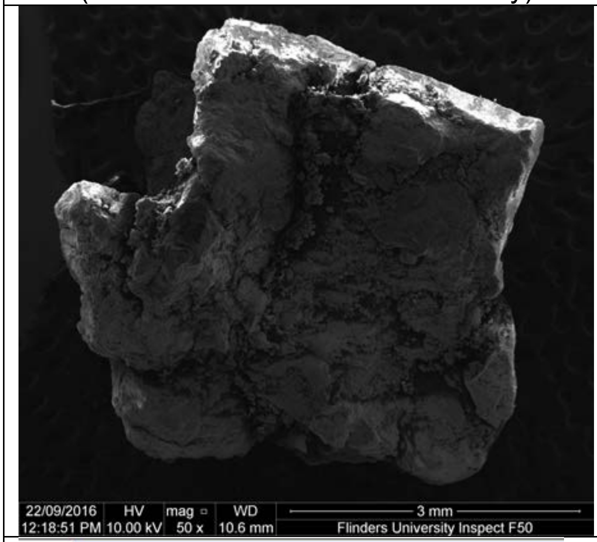	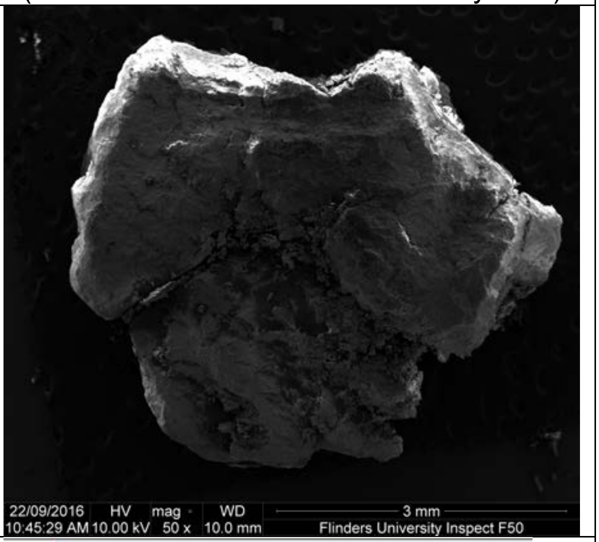
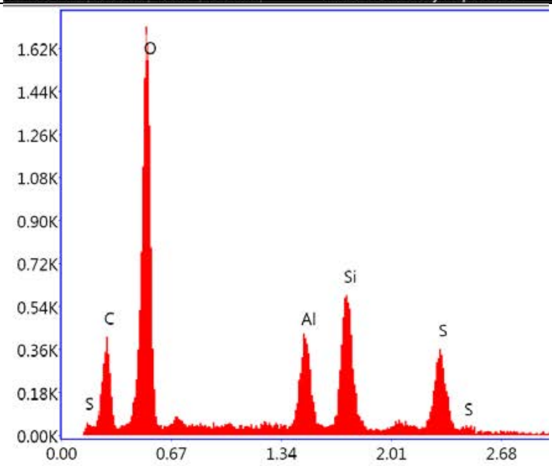	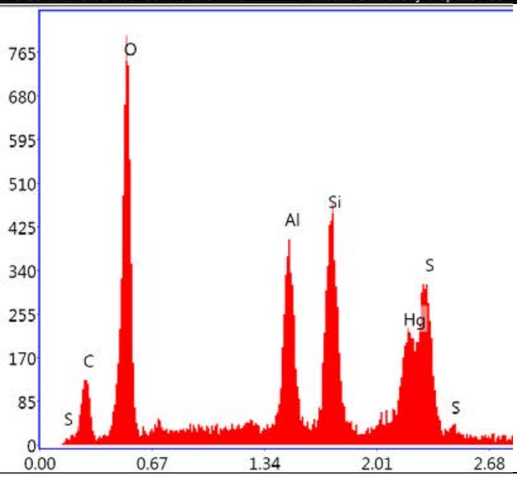
Polymer mixed with soil, separated using a sieve and then washed with 3 x 10 aliquots DI water to remove some of the soil	Polymer mixed with mercury flour, separated using a sieve and then washed with 3 x 10 aliquots DI water to remove some of the soil particles

Fig. S53 | The canola oil polysulfide reacts with mercury flour. SEM and EDS analysis of the particles isolated from the soil after treatment are shown. The particle isolated from the mercury flour clearly trapped mercury.

Toxicity Studies

Cell culture. Huh7 and HepG2 (ATCC® HB-8065™) cells were routinely grown in a humidified incubator at 37 ºC under 5% CO_2, and split before reaching confluence using TrypLE™ Express. Both cell lines were grown on DMEM medium supplemented with 10% heat-inactivated FBS, 2 mM GlutaMAX™, 10 mM HEPES, 1% NEAA, 1 mM sodium pyruvate, 100 units/mL penicillin and 100 µg/mL streptomycin. All reagents were bought from Gibco, Life Technologies (USA), unless otherwise stated.

Cytotoxicity of mercury-treated and untreated polysulfides in HepG2 and Huh7 cells. Cells were cultivated as described above and seeded in 24 well-Transwell® plates at a concentration of 30 000 cells/well (300 µl), and allowed to adhere to the bottom of the well for 24 h. At this point, culture medium was removed and 200 µl of fresh complete medium was added to the bottom layer. Also, 3.75 mg or 37.5 mg of treated or untreated polysulfide was added to each insert in technical duplicates, and 100 µl of complete medium was added on top of the polysulfide, thus creating a continuous layer of medium on top of the cells and the polysulfides. Cells were incubated for another 22 h 30, at which time cell viability was assessed as described above. Results are shown as average of 3 independent experiments (bars), and error bars represent standard error of the mean. There was no difference in cell viability for the cells treated with polymer and cells treated with the polymer-bound mercury. Under these conditions, neither the polymer nor the polymer-bound mercury exhibit significant toxicity.

Notes for Figs S54 and S55:
Dose 1 = 37.5 mg of polymer in 300 mL of culture medium
Dose 2 = 3.75 mg of polymer in 300 mL of culture medium

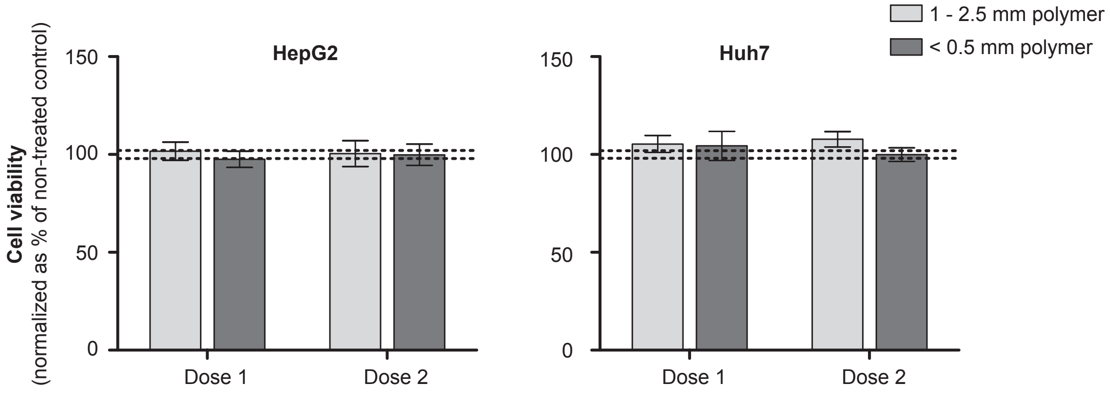

Fig. S54 | Cell viability of HepG2 and Huh7 cell lines grown in presence of canola oil polysulfide (50% sulfur). There was no difference in cell viability for the cells treated with polymer and cells that were untreated. This experiment demonstrates that the polysulfide is not toxic.

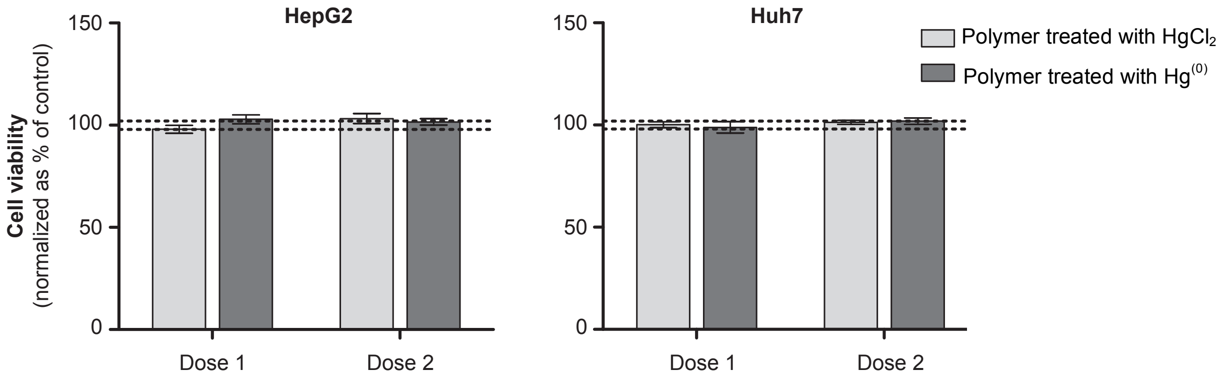

Fig. S55 | Cell viability of HepG2 and Huh7 cell lines grown in presence of polymer-bound mercury. There was no difference in cell viability for the cells treated with polymer and cells treated with the polymer-bound mercury. Under these conditions, neither the polymer nor the polymer-bound mercury exhibit significant toxicity. The polymer treated with $HgCl_2$ contained 2.2 mg $HgCl_2$ per gram of polymer. The polymer treated with $Hg^{(0)}$ contained 79 mg mercury per gram of polymer

Cytotoxicity and estimation of IC_{50} of $HgCl_2$ in HepG2 and Huh7 cells. Cytotoxicity of $HgCl_2$ was assessed using a CellTiter-Blue® Cell Viability Assay (Promega, USA), a fluorescent dye approach based on the ability of metabolically active cells to convert the dye resazurin to the fluorescent resorufin product. Briefly, cells were seeded at a concentration of 10 000 cells/well (100 µL) in flat-bottom 96 well-plates and allowed to adhere and adapt to the plates for 24 h. At this point, culture medium was exchanged to complete medium supplemented with increasing concentrations of $HgCl_2$ in technical triplicates (1, 5, 10, 30, 60, 80, 100 µM). Plates were incubated for 22 h 30 min, at which time cell viability was assessed by exchanging the culture medium to medium supplemented with CellTiter-Blue Reagent (dilution 1:20 from commercial stock) and incubated for another 1 h 30 min, before analysis of fluorescence on an Infinite M200 (Tecan, USA) plate-reader (λ_{exc}=530, λ_{em}=590). Relative fluorescence units (R.L.U.) were normalized to the values obtained for the appropriate vehicle controls. Results are shown as average of 3 independent experiments. A sigmoidal curve (variable slope) was fitted to each dataset, using GraphPad Prism v5 software, and used to calculate the half maximal inhibitory concentration (IC_{50}) of $HgCl_2$ on both cell-lines. The average IC_{50} was 40 µM for HepG2 cells and 34 µM for Huh7 cells.

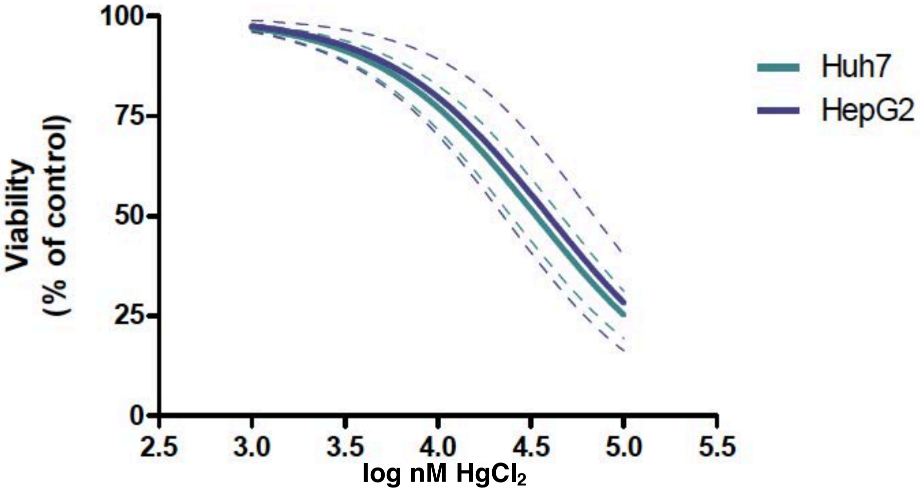

Fig. S56 | Dose-response curve and IC_{50} measurement of $HgCl_2$ in HepG2 and Huh7 cells. The average IC_{50} was 40 µM for HepG2 cells and 34 µM for Huh7 cells.

Synthesis of a porous canola oil polysulfide (50% sulfur) using a sodium chloride porogen

Sulfur (3.00 g) was added to a 250 ml round bottom flask equipped with a 40 mm oval stirring bar. The flask was then placed in a DrySyn reactor cup preheated to 180 °C. The mixture was stirred slowly as the sulfur melted and turned into an orange liquid. At this time, canola oil (food grade, 3.00 g) was added dropwise over 2 minutes to maintain a temperature near 180 °C. After the addition of the canola oil, sodium chloride (14.00 g, previously powdered using a mortar and pestle) was added in several portions over 10 minutes. The addition of the sodium chloride results in a thick, paste-like mixture. The rate of stirring was adjusted to ensure steady mixing. Typically 15-20 minutes after the addition of sodium chloride was complete, the reaction mixture vitrifies and turns into a hard brown solid. After vitrification, the flask was removed from the DrySyn heater and allowed to cool for 1 hour. The product was removed from the flask using a spatula and then milled for 1 minute in a blender (8.5 cm rotating blade). The resulting material (20.0 g) was then transferred to a beaker, followed by 150 mL of deionised water. The mixture was stirred for 1 hour at room temperature to leach the sodium chloride from the polymer particles. The particles were isolated by filtration and then washed a second time in the same manner to ensure the complete removal of sodium chloride. Isolating the polymer by filtration and then drying under high vacuum provided the final porous polymer as a sponge-like material (6.0 g). If residual sodium chloride is observed on the surface of the polymer or by SEM, additional water washes can be used.

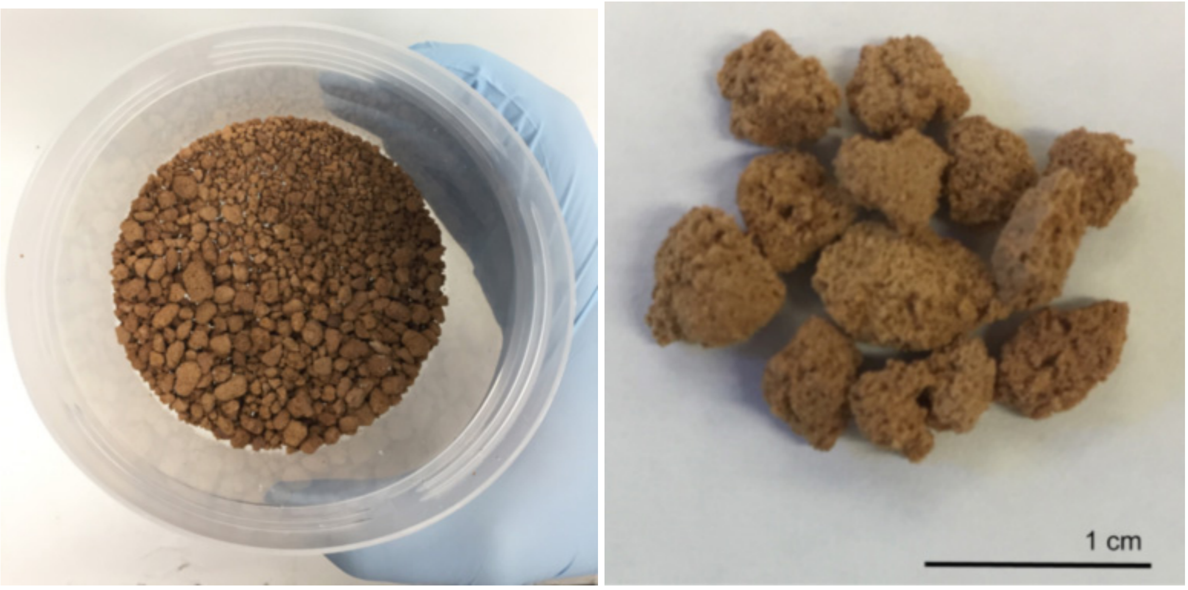

Fig. S57 | Samples of the porous canola oil polysulfide (50% sulfur) prepared using sodium chloride as a porogen.

SEM analysis of porous canola oil polysulfide (50% sulfur)

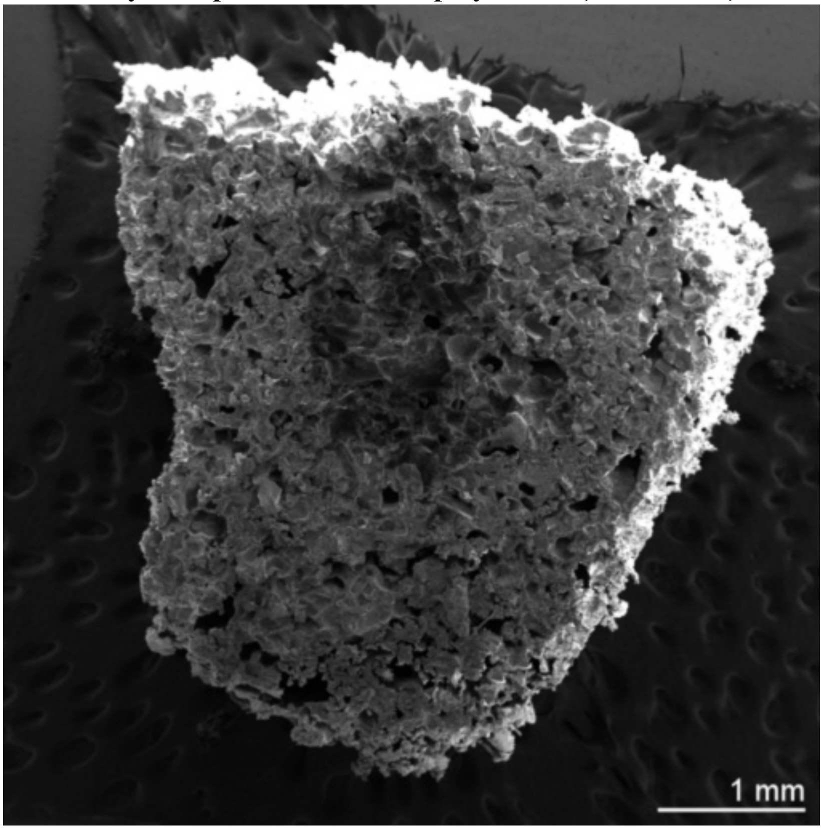

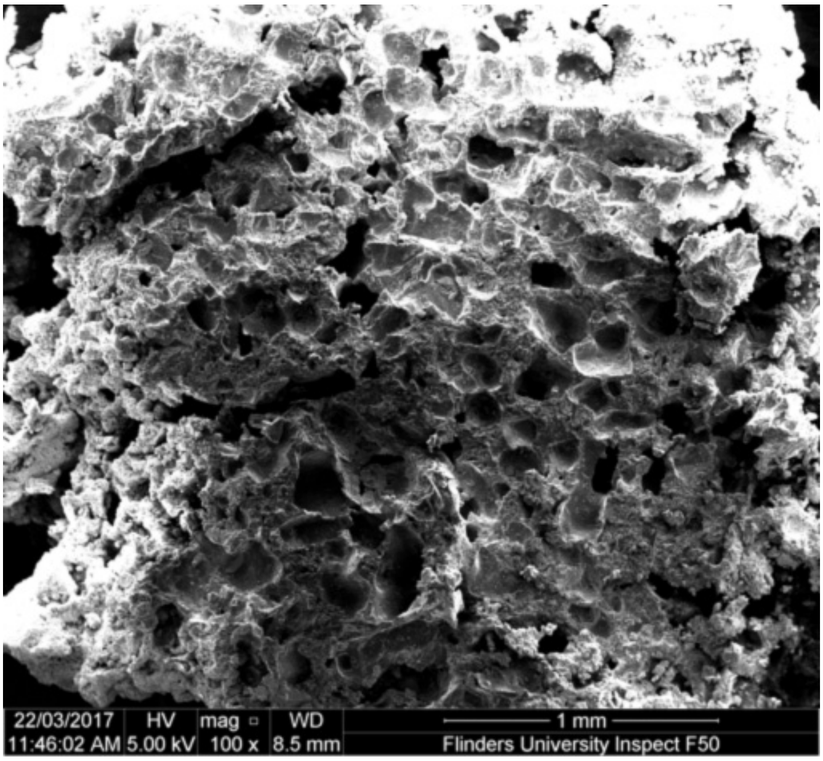

Fig. S58 | Representative SEM images of the porous canola oil polysulfide. Channels and pits (~100-300 μm in diameter) were formed by leaching out the sodium chloride porogen with water.

Raman spectroscopic analysis of porous canola oil polysulfide (50% sulfur)

A sample of the porous canola oil polysulfide was analysed at 20 points over a 400 × 400 micron area using an XplorRA Horiba Scientific Confocal Raman microscope. As with the non-porous polymer, signals at 434 and 467 cm^{-1} are consistent with S-S vibrational modes of a polysulfide material and signals at 1438 cm^{-1} and 2903 cm^{-1} are attributed to the canola oil domain of the polymer. Representative Raman spectra are shown for regions rich in the canola oil polysulfide (both S-S and canola oil signals visible) and regions rich in primarily S-S (free sulfur or long stretches of polysulfide). These spectra are highly similar to the non-porous polysulfide.

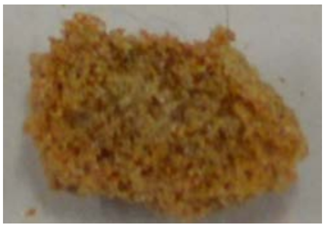
~5 mm diameter particle of porous polymer used in the Raman analysis.

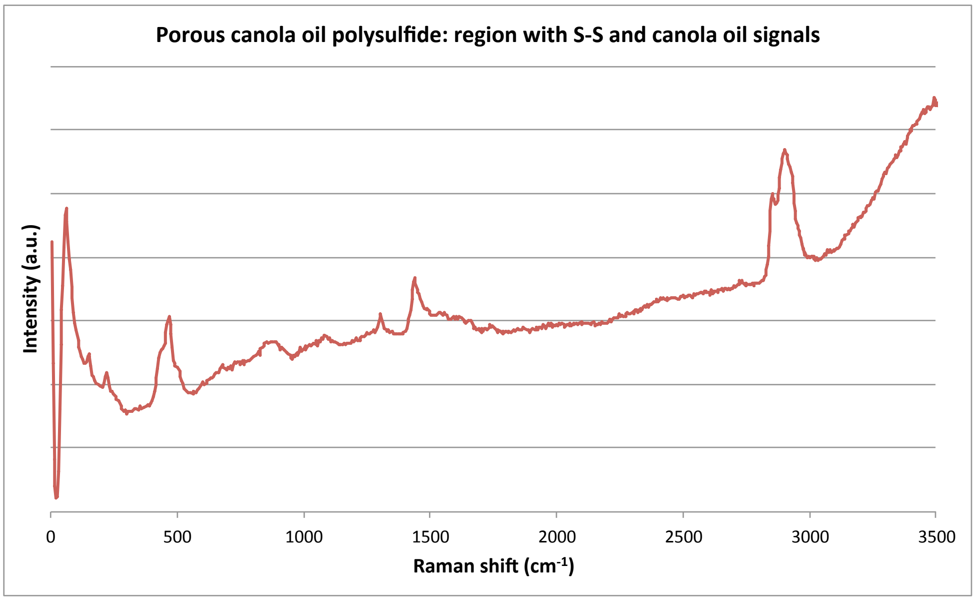

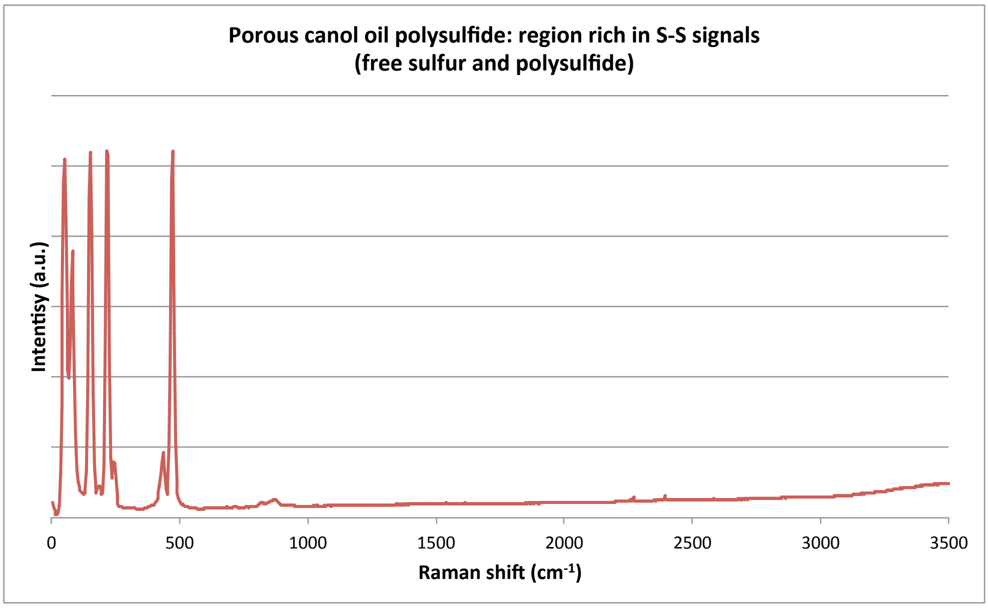

Fig. S59 | Representative Raman spectra obtained on a sample of the porous canola oil polysulfide

TGA and DSC of porous canola oil polysulfide (50% sulfur)

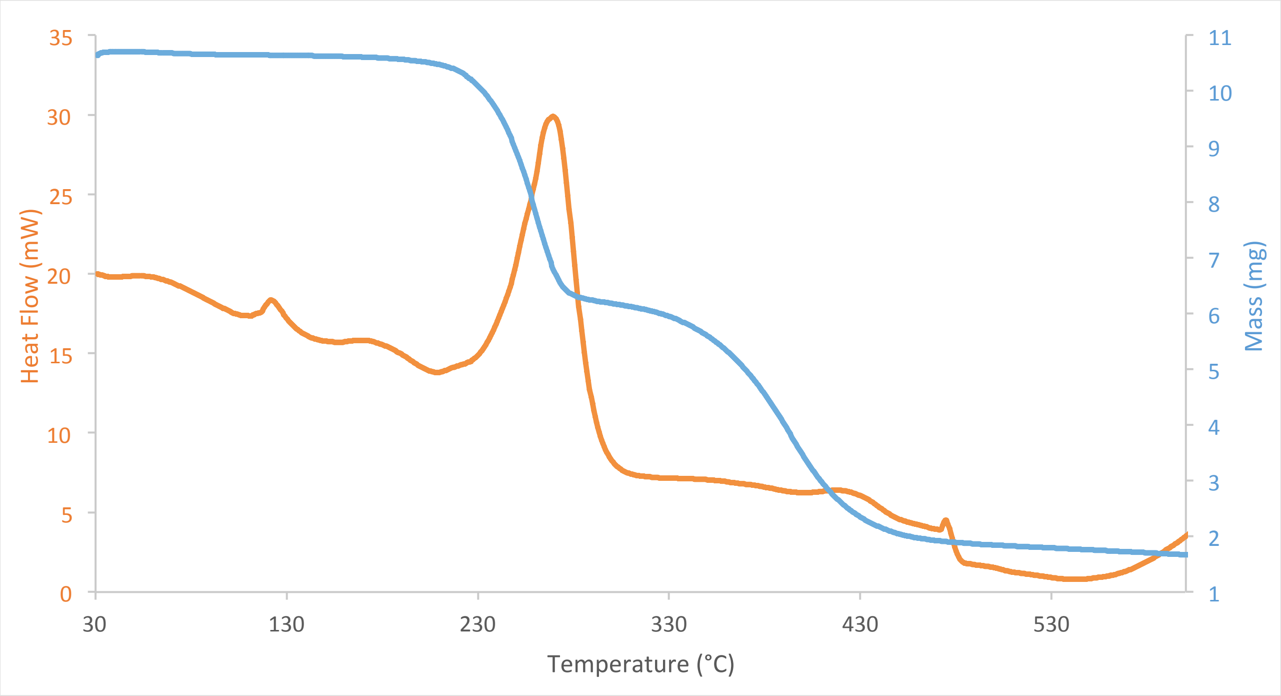

Fig. S60 | DSC (orange) and TGA (blue) traces for the porous canola oil polysulfide. This data indicates the thermal properties are highly similar to the non-porous polysulfide (cf. Fig S18 and Fig. S21).

The amount of free sulfur in the porous canola oil polysulfide was estimated to be 13% by mass, as determined by integration of the peak from 100 ºC to 150 ºC (see Fig 23 for the analogous experiment for the non-porous polysulfide and calibration curve).

Determination of glass transition temperature by DSC for porous polysulfide (50% sulfur)

The glass transition temperature of the porous canola oil polysulfide was -12.9 ºC, as determined by DSC:

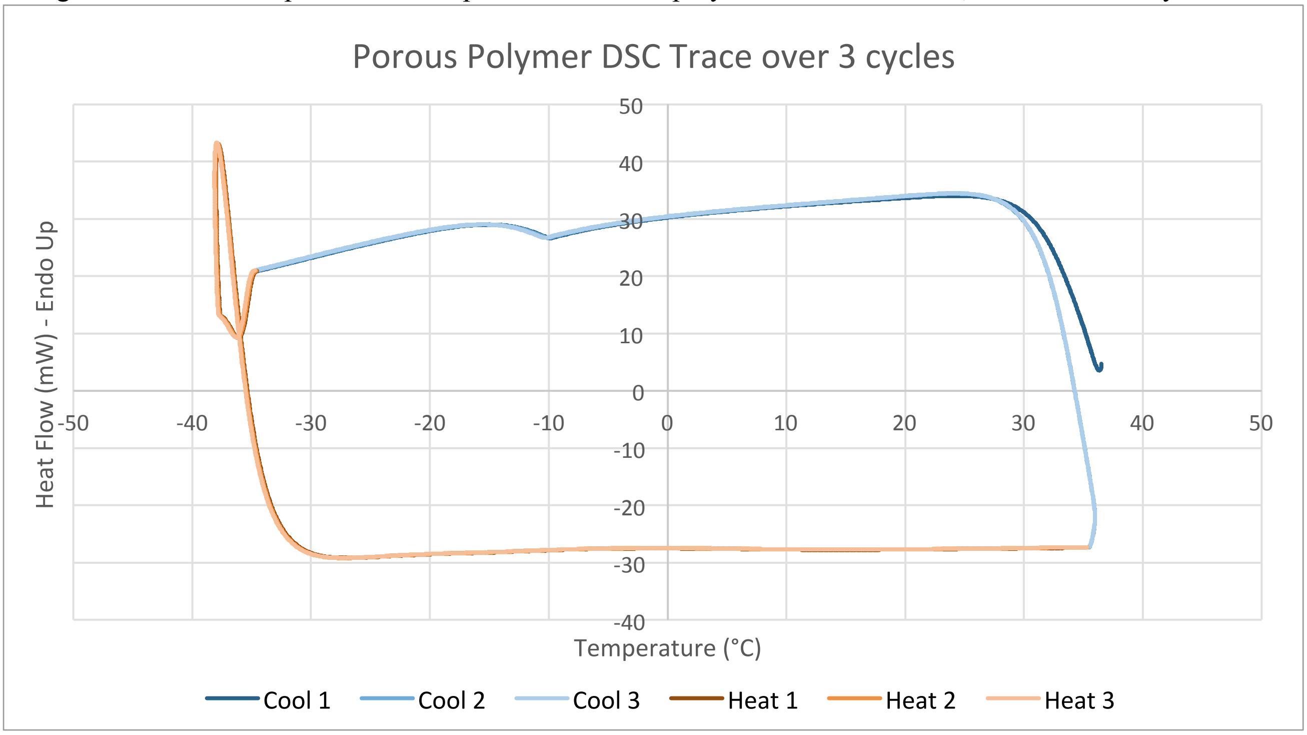

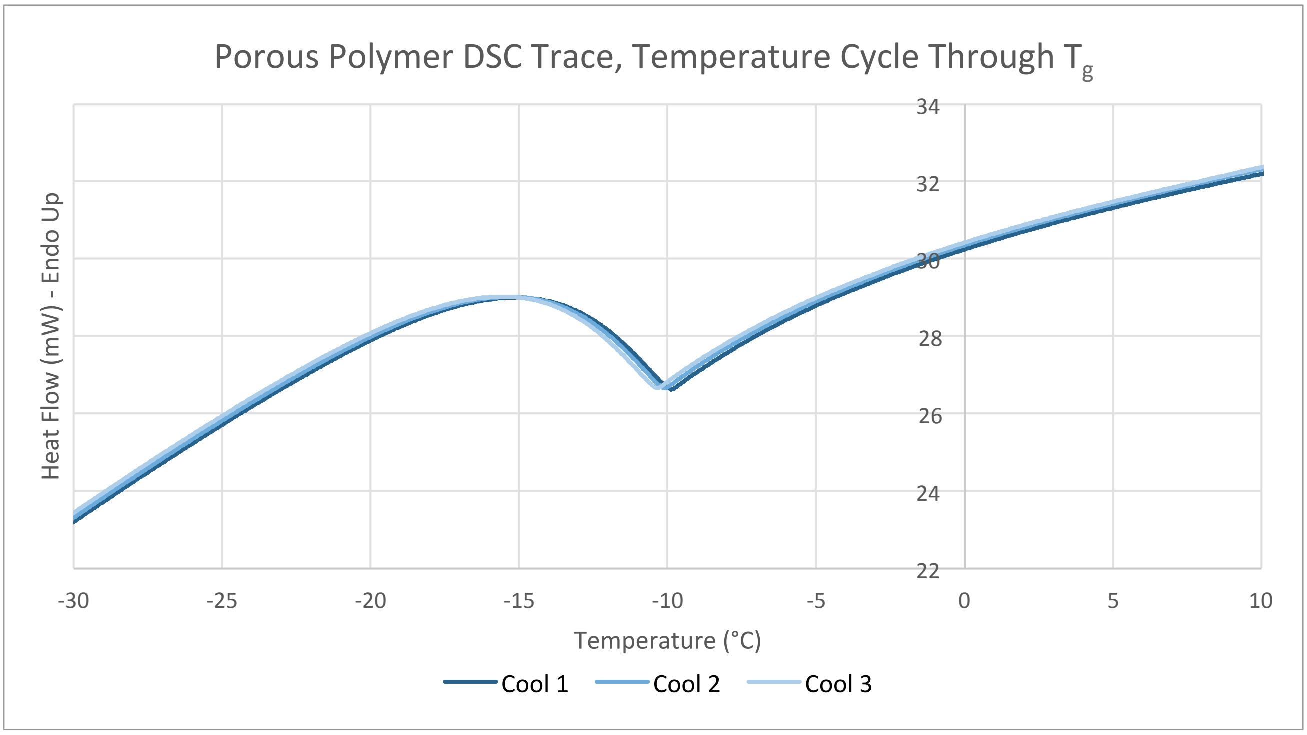

Fig. S61 | Determination of T_g using DSC for the porous Canola Oil Polysulfide prepared at 50% sulfur.

NMR analysis of $CDCl_3$ soluble fraction of porous canola oil polysulfide (50% sulfur).

$CDCl_3$ (10 mL) was added to a sample of the porous polysulfide (508 mg). The mixture was stirred vigorously at room temperature for 15 minutes to extract the soluble fraction of the polymer. The resulting solution was filtered and then analysed by ^{1}H NMR. The spectra are shown below with unreacted canola oil as a reference. The key findings from this experiment are that the soluble fraction of the polymer contains unreacted alkene peaks. However, even in this soluble fraction the ratio of the terminal methyl groups of the fatty acid ester (0.87 ppm) and the alkene peaks (5.25-5.37 ppm) have changed. In the canola oil this ratio is 1.0 : 1.0. and in the soluble fractions of the polymer it is ~3:1, indicated alkene reaction. After NMR analysis, the solvent was evaporated to determine the amount of polymer dissolved. For the porous canola oil polysulfide, 111 mg dissolved (22% polymer mass). From this result, it can be concluded that in the polymerisation of canola oil and sulfur at a mass ratio of 1:1, the gel point is reached before all alkenes are consumed.

^{1}H NMR of canola oil (before polymerisation with sulfur)

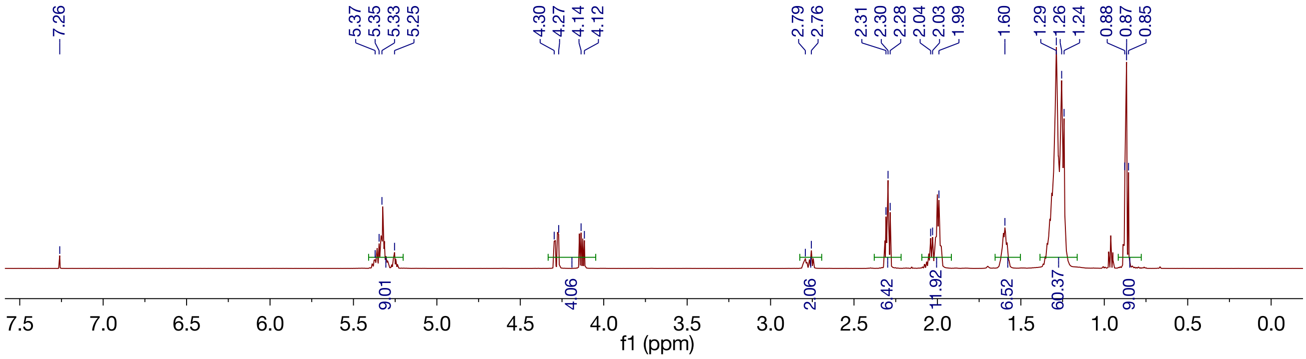

^{1}H NMR of $CDCl_3$ soluble fraction of porous canola oil polysulfide

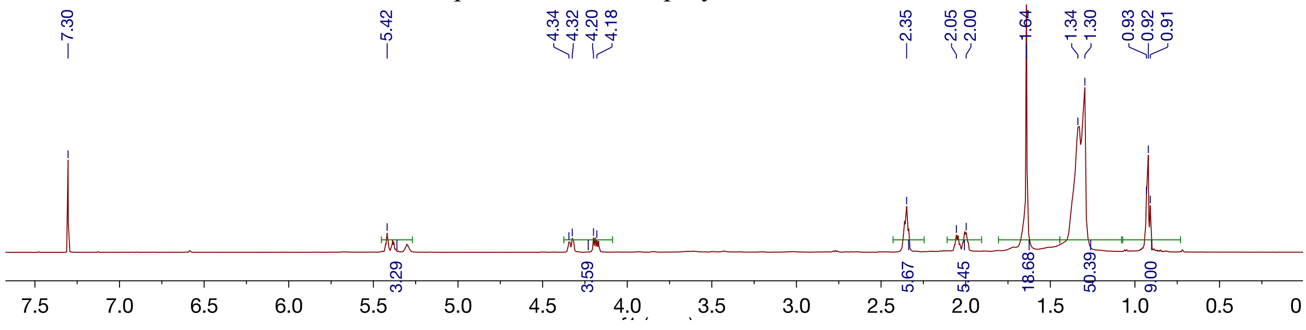

Fig. S62 | ^{1}H NMR of canola oil, and the soluble fractions of the porous polysulfides

Mercury vapour experiments using the porous canola oil polysulfide

Hg^0 removal tests were performed using a fixed bed-reactor as shown in Fig. S63. The inlet Hg^0 vapour was generated using a mercury permeation device (VICI metronics), which was operated at 60 °C. The porous canola oil polysulfide (300 mg) was placed in the quartz glass reactor (1 cm internal diameter), occupying a volume of approximately 0.4 cm^2. N_2 gas with a flow rate of 0.1 L/ min, which contained 586.4 µg/Nm^3 Hg^0, was introduced to the reactor using mass flow controllers. At this volume of sorbent and flow rate, the residence time is 0.24 seconds—a challenging test for the polysulfide sorbent. All elemental and oxidised mercury exiting the reactor were measured quantitatively using a modified Ontario Hydro Method (OHM), in which KCl (0.01 M) and $KMnO_4/H_2SO_4$ (20 mg L^{-1}) impinger solutions were used in the train of traps as mercury absorbing media. Elemental mercury (Hg^0) is captured by the $KMnO_4$ solution, whereas any oxidised mercury (Hg^{2+}) is trapped by the KCl solution. The remaining adsorbed mercury was retained on the canola oil polysulfide. Cold vapour atomic fluorescence spectroscopy (CV-AFS) was used to measure the collected Hg from the system after the Hg^0 removal experiments. In all experiments, the amount of oxidised mercury (Hg^{2+}) collected from the KCl traps was negligible (<< 1% of total Hg). Hg^0 removal efficiency of material was determined by the following equation:

$$Hg^0 \text{ removal efficiency } (\%) = \frac{Hg^0_{in} - Hg^0_{out}}{Hg^0_{in}} \bullet 100\ (\%)$$

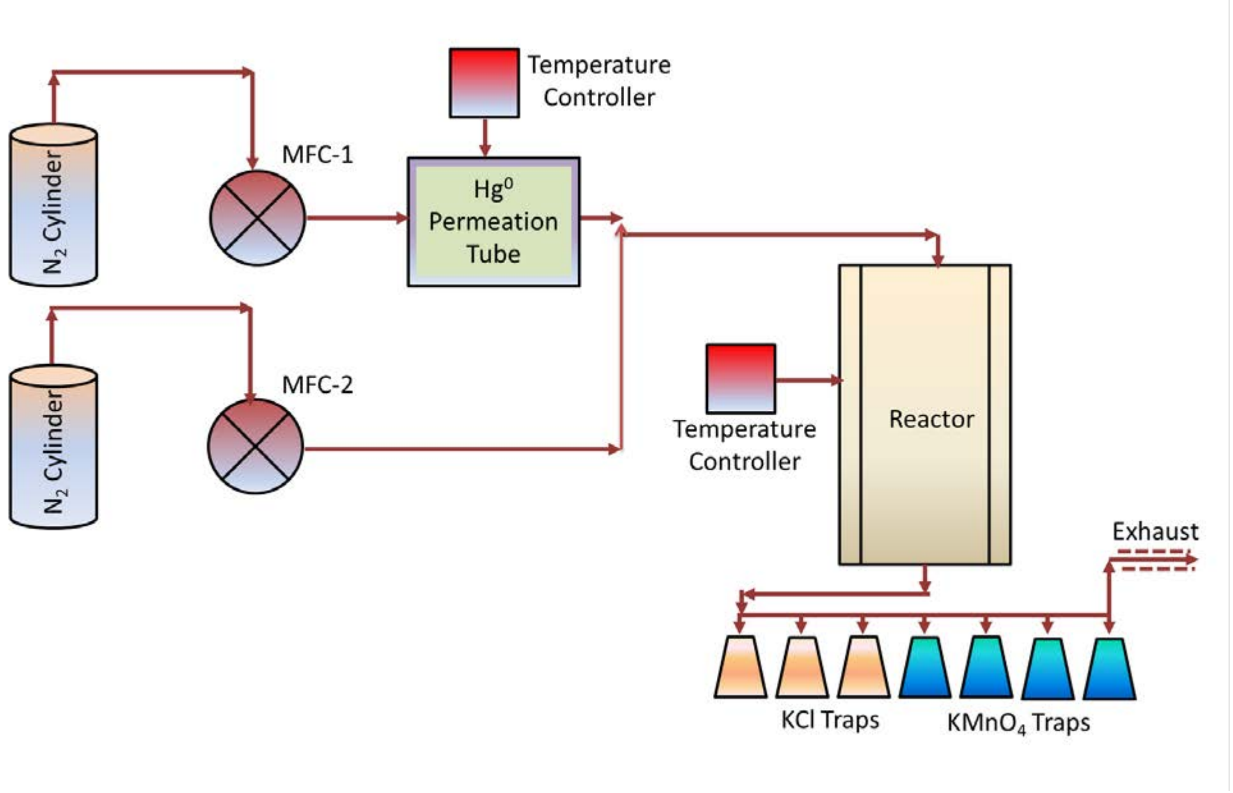

Fig. S63 | Schematic diagram of the experimental setup for testing the canola oil polysulfide as a sorbent for mercury vapour.

The effect of operating temperature on mercury removal efficiencies of the developed material was tested by varying the reactor temperature from 25–100 °C. It was hypothesised that the rate of reaction between the mercury vapour and the polysulfide would increase with temperature—a requirement for continuous processes

with short residence times such as those in this experiment. It was found that the material had highest Hg^0 removal efficiency of 66.5 % at 75 °C.

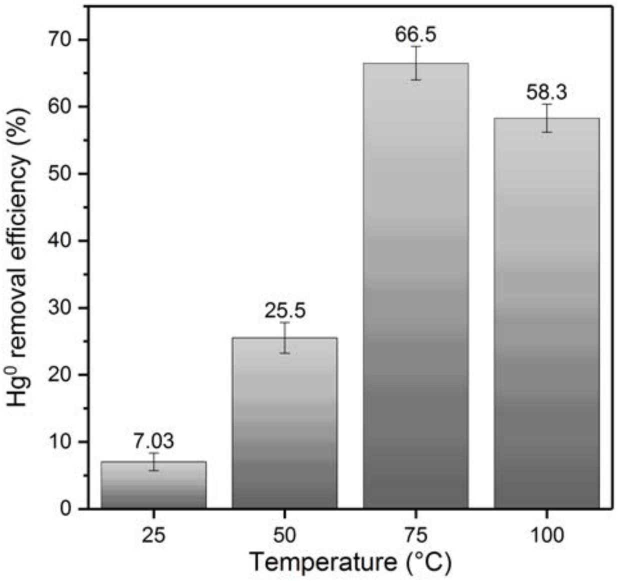

Fig. S64 | 75 ºC was found to be an optimal temperature for capturing mercury in a continuous process, with 66.5% of the mercury removed from the gas stream over a residence time of approximately 0.24 seconds.

Installation of thiols on the porous canola oil polysulfide by partial reduction with $NaBH_4$

The porous canola oil polysulfide (2.00 g) was added to a 100 mL round bottom flask along with 34 mg sodium borohydride. Methanol (10 mL) was added carefully and the reaction mixture was stirred open to air for 1 hour. After this time the reaction was quenched with 10% HCl (10 mL) and then diluted further with 10 mL H_2O. The resulting product was isolated by filtration and dried under vacuum. This material was positive in an Ellman's test in which 10 mg of the Ellman's reagent was added to a sample of the polymer in 10 mL phosphate buffer (100 mM, pH 8.0), indicating the presence of thiols on the surface of the polymer. The partially reduced polymer was very similar in appearance to the original porous polysulfide. Using a larger excess of sodium borohydride (>500 mg) led to substantial degredation and loss of porous and particulate character, consistent with reduction of the polysulfide.

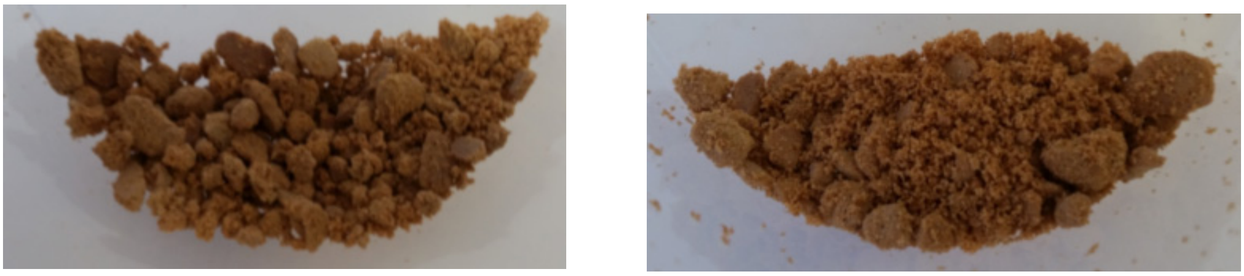

Fig. S65 | The porous canola oil polysulfide (left) and a partially reduced canola oil polysulfide (right)

Experiments on mercury bound to natural organic matter (NOM)

Materials and Methods

Mercury speciation can significantly affect reactivity of mercury and its interaction with sorbent materials. The speciation of mercury in aquatic ecosystems is typically dominated by association with natural organic matter (NOM). Suwannee River aquatic natural organic matter (SR-NOM), reference material 2R101N (International Humic Substance Society) and a 1 ppm $Hg(NO_3)_2$ standard (Brooks Rand Instruments, Seattle, WA, USA) were used to prepare Hg-NOM complexes containing 40 µg/L Hg and 2400 µg/L total carbon (C_{NOM}) equivalent to a molar Hg:C_{NOM} ratio of $1.8 \cdot 10^{-5}$. SR-NOM was dissolved in 10 mM sodium phosphate buffer (pH 7.8) and filtered through a 0.2 µm syringe filter to remove residual particulates. $Hg(NO_3)_2$ was added and the pH was re-adjusted to 7.8 and allowed to age at 4 ºC for at least 5 days. The Hg-NOM stock solution was diluted with 10 mM sodium phosphate buffer to obtain working solutions with Hg concentrations from 0.2 to 7.7 µg/L. A dilution series of the 1 ppm $Hg(NO_3)_2$ standard in 10 mM sodium phosphate buffer was prepared as an NOM-free control.

Sorption isotherms were determined in triplicate batch experiments by adding 30 mL Hg-NOM complex at Hg concentrations of 0.2, 0.4, 0.7, 1.5, 3.6 and 7.7 µg/L or $Hg(NO_3)_2$ in phosphate buffer at concentrations of 0.2, 0.5, 0.8, 1.6, 4.0 and 16 µg/L to 40 mL amber borosilicate glass vials containing approximately 100 mg of canola oil polysulfide (COP), porous COP or partially reduced porous COP after equilibration for 48 hours on a rotary shaker. The suspensions were filtered through a 0.2 µm polyethersulfone (Supor®) syringe filter for total mercury and sulfate analyses by ion chromatography. To determine Hg equilibrium concentrations, 5 mL of the filtered samples were oxidized by addition of 150 µL BrCl. An aliquot of this solution was added to an excess of 20% (w/v) stannous chloride and purged with ultrahigh purity N_2. The amount of emerging Hg^0 was determined by a cold vapor atomic absorption spectroscopy (CV-AAS) Zeeman effect mercury analyzer (Lumex RA-915+, Ohio Lumex Company, Inc., Twinsburg, OH, USA). The concentration of sorbed Hg was determined by difference between the known initial amount of Hg added and the equilibrium aqueous Hg concentrations, which also included Hg sorbed to the wall of the amber glass vials

Results & Discussion

Within the tested concentration ranges, a linear correlation was obtained for the sorption to all COP variants when Hg was added as $Hg(NO_3)_2$. The sorption isotherms with Hg added as Hg-NOM show a nonlinear characteristic, which was approximated by the Langmuir isotherm model. The Langmuir adsorption isotherm assumes monolayer adsorption onto a surface containing a finite number of uniform adsorption sites. The surface reaches a saturation point, where maximum sorption of adsorbate on a monolayer is reached. The relationship between adsorbed and solution concentrations for the Langmuir isotherm is as follows.

$$Y = \frac{Y_{\max} \cdot K_L \cdot C_{eq}}{1 + K_L \cdot C_{eq}}$$

Where Y is the concentration of the adsorbate on the sorbent, Y_{max} is the sorption capacity, C_{eq} is the solution concentration at equilibrium and K_L is the Langmuir adsorption equilibrium constant. The isotherm fits for all COP variants are shown in Fig. S66. The results show that all tested COP samples removed >90% of Hg when added as $Hg(NO_3)_2$. The strong complexation of mercury with functional groups on NOM competes with the sorption of Hg to any sorbent, thus presenting a unique challenge for the removal of Hg from contaminated ecosystems. Under the conditions of the isotherm experiments, a dilution series was prepared from a concentrated Hg-NOM stock solution. Thus, the concentration of Hg is coupled the concentration of NOM. In a freshwater creek ecosystem, the level of NOM can span a wide range of concentrations, while the level of Hg typically corresponds to the low end of the experimental range, even in contaminated systems.[3] Efficient removal of Hg from solutions containing strong Hg-NOM complexes is achievable as it is determined by the sorbent to solution ratio and the concentration of Hg-NOM. A measure of how efficient the sorbent can remove the contaminant at a specific concentration can be obtained as follows:

$$R[\%] = \frac{C_0 - C_{eq}}{C_0} \cdot 100$$

Where R is the removal efficiency, C_0 is the initial Hg concentration and C_{eq} is the Hg concentration after equilibration with the sorbent. Surface modification of canola polysulfide had a significant impact on Hg removal, with the higher surface area of the porous versions significantly improving removal efficiency. At the lowest initial Hg-NOM concentrations (0.2 µg/L Hg) and a sorbent to solution ratio of 1/300, R was 36% for non-porous canola oil polysulfide, 79% for porous canola oil polysulfide, and 81% for the partially reduced porous polysulfide. The results show that the surface modification of COP, particularly the increased surface area in porous COP, results in a highly effective sorbent which can sorb Hg in the presence of competing ligands such as NOM.

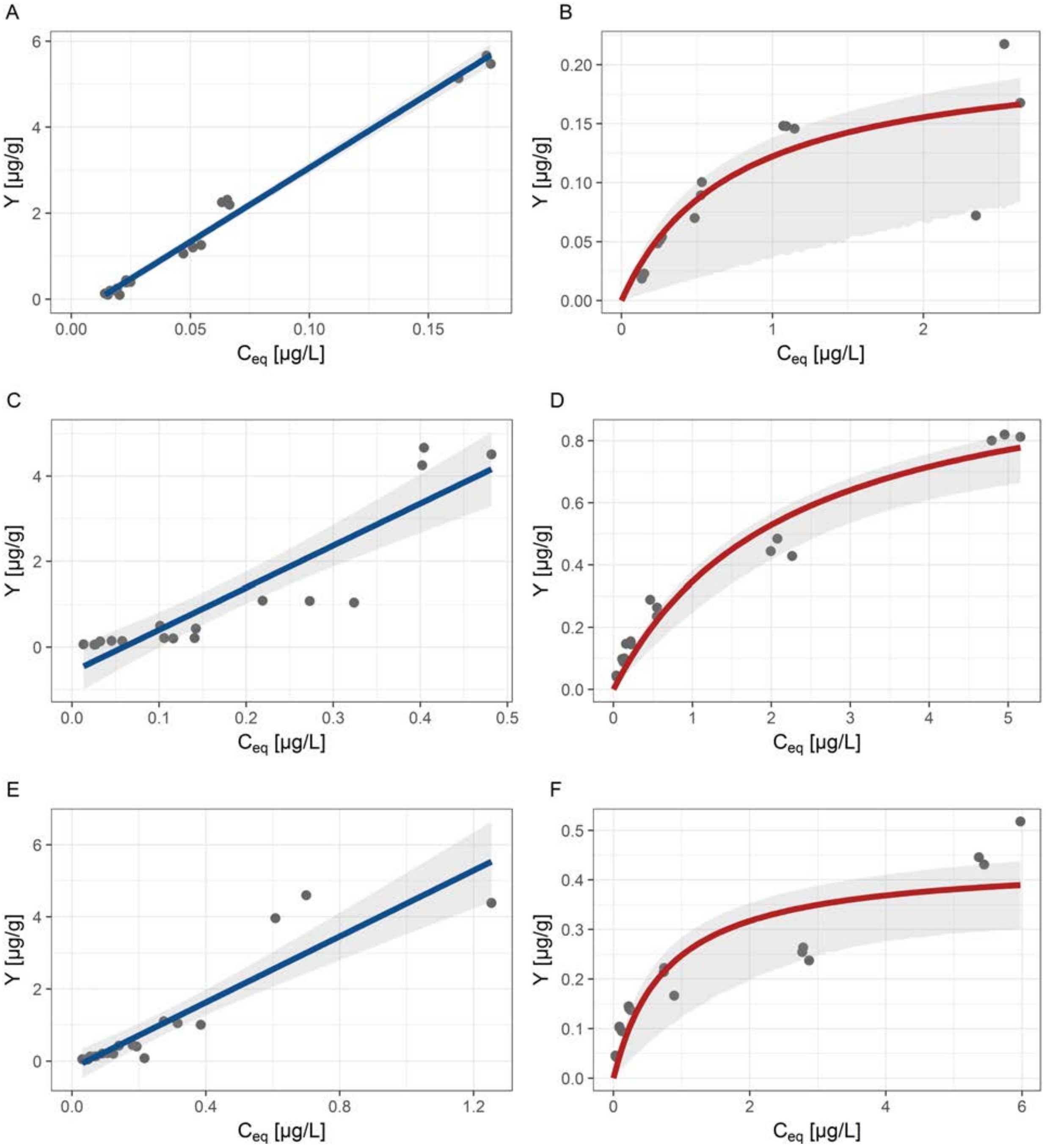

Fig. S66 | Equilibrium sorption data (dots) and fits to isotherm models for the sorption of Hg at low mercury concentrations. 95% confidence bands are shown in gray. A. Unmodified COP with Hg added as $Hg(NO_3)_2$ and linear fit (blue), residual standard error of the fit: 0.21 µg/g; B. Unmodified COP with Hg added as Hg-NOM complex and model fit to the Langmuir isotherm model (red). Langmuir fit parameters: K_L = 1.35 L/µg,

Y_{max} = 0.21 µg/g, residual standard error of the fit: 0.032 µg/g; C. Porous COP with Hg added as $Hg(NO_3)_2$ and linear fit (blue), residual standard error of the fit: 0.71 µg/g; D. Porous COP with Hg added as Hg-NOM complex and model fit to the Langmuir isotherm model (red). Langmuir fit parameters: K_L = 0.46 L/µg, Y_{max} = 1.11 µg/g, residual standard error of the fit: 0.061 µg/g; E. Partially reduced porous COP with Hg added as $Hg(NO_3)_2$ and linear fit (blue), residual standard error of the fit: 0.65 µg/g; F. Partially reduced porous COP with Hg added as Hg-NOM complex and model fit to the Langmuir isotherm model (red). Langmuir fit parameters: K_L = 1.29 L/µg, Y_{max} = 0.44 µg/g, residual standard error of the fit: 0.065 µg/g

Sulfate release from the porous canola oil polysulfide

High sulfate concentrations in low oxygen subsurface environments can result in increased production of methylmercury. Sulfate-reducing bacteria have been associated with mercury methylation and are considered the primary methylators in marine and estuarine environments.[4,5] We therefore determined sulfate concentrations in solutions obtained from batch sorption studies (see Fig. S67). Briefly, 30 mL Hg-NOM complex dissolved in 10 mM sodium phosphate buffer (pH 7.8) at various concentrations were added to amber glass vials containing approximately 100 mg of canola oil polysulfide and equilibrated for 48 hours on a rotary shaker. The solid to solution ratio was constant for all samples. Sulfate concentrations were determined by ion chromatography with a Dionex ICS 2100 AS9HC9 (Dionex Instruments Corporation, Sunnyvale, CA, USA) from filtered sample solutions using 9 mM K_2CO_3 as the eluent. The amount of sulfate released was normalized to the mass of the polysulfide for each sample. The amount of sulfate released correlated with the concentration of Hg-NOM initially added to the sample (Fig. S67). In the absence NOM, sulfate concentrations were typically <100 µg per g of sorbent. For samples containing NOM, the sulfate concentration was proportional to the NOM concentration.

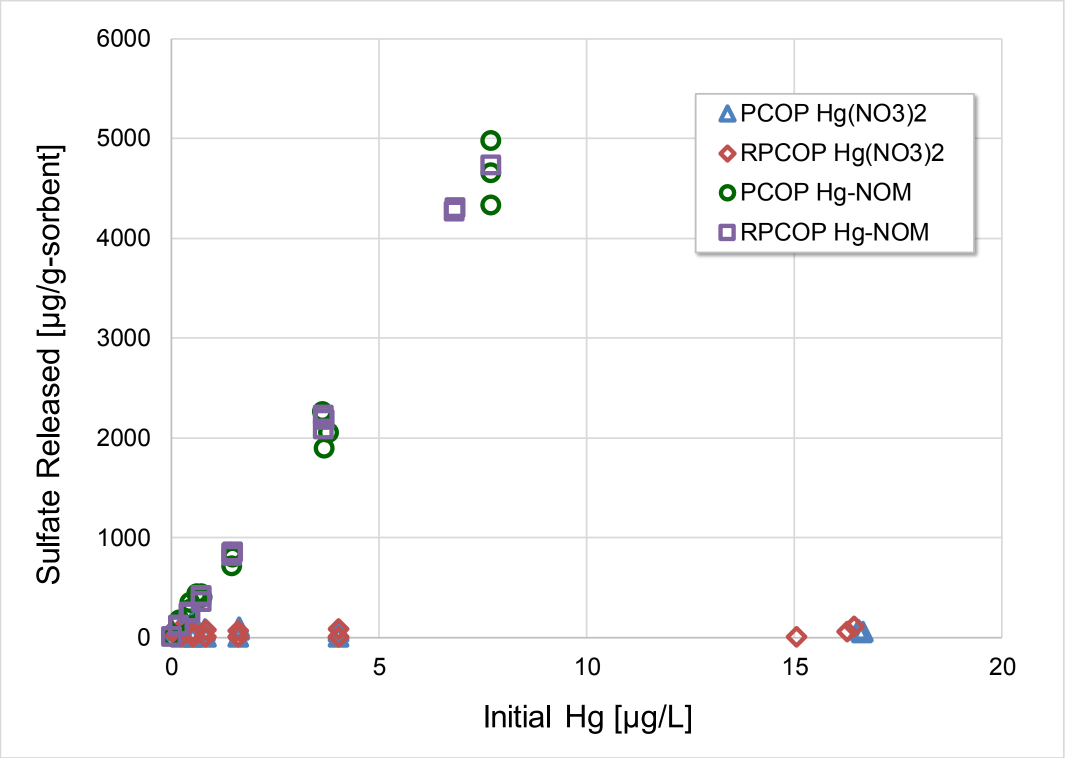

Fig. S67 | Sulfate concentrations normalized to mass of sorbent in 48 h batch equilibrium experiments for porous canola oil polysulfide (PCOP) and the partially reduced porous canola oil polysulfide (RPCOP).

Experiments using an organomercury fungicide [2-methoxyethylmercury chloride (MEMC)]

Batch remediation

A solution of agriculture grade 2-methoxyethylmercury chloride (MEMC) was prepared at a concentration of 0.15 g/L mercury—a concentration typically employed when applying this material as a fungicide. A 10 mL aliquot of this solution was added to each of six 50 mL centrifuge tubes. To three of these tubes was also added 2.00 g of the porous canola oil polysulfide. The three remaining samples were used as negative controls in which no polymer was added. The samples were incubated without agitation for 24 hours. After this time, 100 μl aliquots were sampled from each tube and then diluted 100,000-fold into a 2% HNO_3 matrix and analysed for Hg content on a Perkin Elmer NexION ICP-MS in KED mode (Flinders Analytical, South Australia). Calibration standards were prepared from a stock solution of 1,000 ppm Hg in 2% HNO_3 (Chem-Supply, South Australia). No change in mercury concentration was observed for the untreated solution of MEMC (14.7 ± 0.3 ppb mercury). The solutions of MEMC that were treated with polymer, in contrast, had a 98% reduction in dissolved mercury (0.96 ± 0.01 ppb mercury). The concentrations reported refer to the diluted sample analysed directly in the ICP-MS runs. A control in which the porous polysulfide was added to water only was found to have 0.08 ± 0.02 ppb mercury.

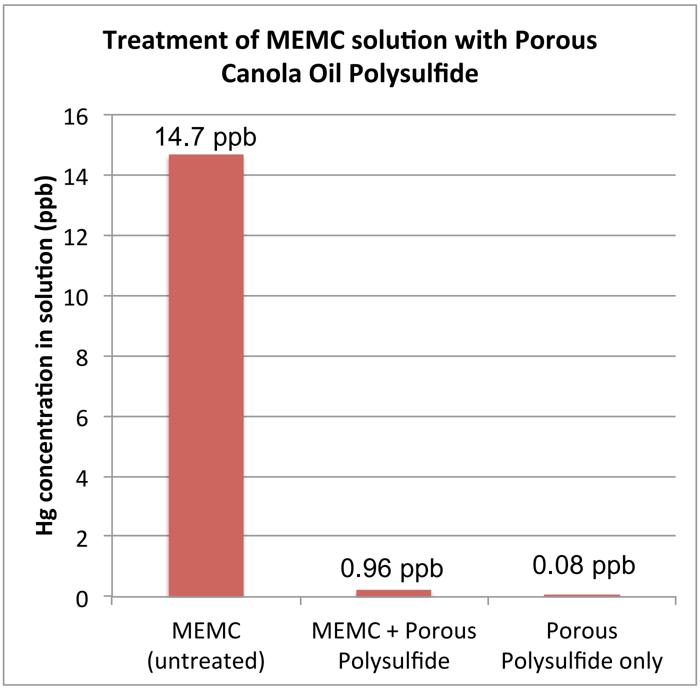

Fig. S68 | The porous canola oil polysulfide removes 98% of mercury from an aqueous solution of MEMC

Mercury removal from a MEMC solution using columns prepared from soil and the porous canol oil polysulfide

Four types of columns were prepared (in triplicate) in the barrel of a 10 mL syringe. The plunger was first removed and cotton wool was used to plug the outlet. The column was then packed with one of the following: soil (3.0 g); soil (1.5 g) and porous polysulfide (1.5 g) mixed together; soil (1.5 g) layered on top of a layer of porous polysulfide (1.5 g), separated by cotton; or porous polysulfide (3.0 g). A solution of MEMC was prepared at 0.15 g/L and then 3 mL of this solution was added to the column by pipette. The plunger of the syringe was carefully re-inserted and the solution was eluted slowly by applying gentle pressure. The total elution time was approximately 2.5 minutes for each type of column. The flowthrough was collected and sample of each was diluted 100,000-fold in a 2% HNO_3 matrix and Hg content was measured by ICP-MS as described in the previous experiment. The mercury concentration for the MEMC solution before passing through the column was also measured in triplicate. The columns and data are shown below. Soil alone (3.0 g) retained 46% of the mercury; soil and polymer (1.5 g each) mixed randomly together retained 66% of the mercury; soil (1.5 g) layered on top of the polymer (1.5 g) retained 75% of the mercury; and polymer alone (3.0 g) retained 73% of the mercury.

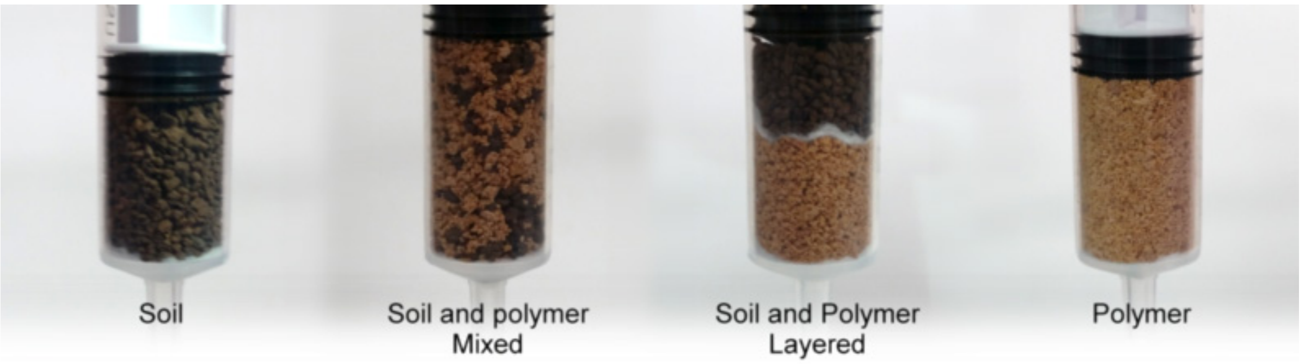

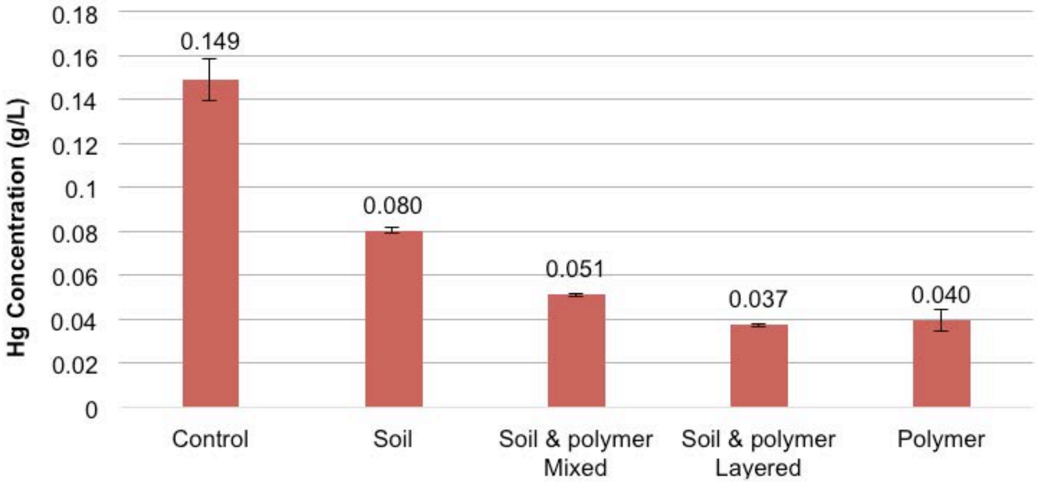

Fig. S69 | Mercury from the MEMC fungicide could be removed from an aqueous solution using a simple syringe filter constructed from the porous canola oil polysulfide and/or soil.

References

1. Hoefling, A., Lee, Y.J. & Theato, P. Sulfur-Based Polymer Composites from Vegetable Oils and Elemental Sulfur: A Sustainable Active Material for Li–S Batteries. *Macromol. Chem. Phys.*, **218,** 1600303 (2017).

2. López, F.A., López-Delgado, A., Padilla, I., Tayibi, H. & Alguacil, F.J. Formation of metacinnabar by milling of liquid mercury and elemental sulfur for long term mercury storage. *Sci. Total Environ.* **408**, 4341-4345 (2010).

3. Southworth, G. *et al.* Sources of mercury in a contaminated stream-implications for the timescale of recovery. *Environ Toxicol Chem* **32**, 764-772 (2013).

4. Gilmour, C.C., Henry, E.A. & Mitchell, R. Sulfate Stimulation of Mercury Methylation in Freshwater Sediments. *Environ. Sci. Technol.* **26**, 2281-2287 (1992).

5. Fitzgerald, W.F., Lamborg, C.H. & Hammerschmidt, C.R. Marine biogeochemical cycling of mercury. *Chem. Rev.* **107**, 641-662 (2007).
